# Global, regional, and national epidemiology and risk factors of geriatric digestive system cancers from 1990 to 2021

**DOI:** 10.3389/fpubh.2025.1629101

**Published:** 2025-10-21

**Authors:** Congcong Xu, Jing Chen, Qingliang Wang, Ziyu Zheng, Chunju Fang, Hanyu Yu, Qingna Liang, Xin Wang

**Affiliations:** ^1^Department of Oncology, Qingdao Central Hospital, University of Health and Rehabilitation Sciences (Qingdao Central Hospital), Qingdao, China; ^2^Department of Hepatobiliary-Pancreatic-Splenic Surgery, The Third Affiliated Hospital, Sun Yat-sen University, Guangzhou, China; ^3^Department of Radiation Oncology, National Cancer Center/National Clinical Research Center for Cancer/Cancer Hospital, Chinese Academy of Medical Sciences and Peking Union Medical College, Beijing, China; ^4^Department of Oncology, Guizhou Provincial People’s Hospital, Guiyang, China

**Keywords:** global burden of disease, age-standardized rate, geriatric digestive system cancers, socio-demographic index, risk factors

## Abstract

**Background:**

Using Global Burden of Disease (GBD) 2021 data, we analyzed incidence, mortality, disability-adjusted life years (DALYs), and risk factors for six geriatric digestive system cancers (GDSC) in adults aged ≥60 years from 1990 to 2021; assessed Socio-demographic Index (SDI) impacts; and projected trends to 2050.

**Methods:**

The joinpoint regression model was used to analyze the age-standardized data for temporal trends; the decomposition method was used to assess the contributions of population growth, aging, and epidemiological changes; and frontier analysis was used to evaluate opportunities for burden reduction across countries.

**Results:**

Globally, the incidence of GDSC increased from 1,876,969 [age-standardized incidence rate (ASIR) 405.48/100,000] in 1990 to 3,854,209 (ASIR 360.89/100,000) in 2021, with a slight decrease in ASIR [−0.57% average annual percentage change (AAPC)]. From 1990 to 2021, global GDSC deaths rose to 2,856,742, but age-standardized mortality rate (ASMR) declined to 270.14, with an AAPC of −1.72%. Gastric cancer showed the largest decline in incidence (−2.21% AAPC), whereas liver cancer increased (0.74% AAPC). Gender disparities were evident, with higher rates in males. High-income Asia-Pacific and East Asia had the highest ASIR and ASMR. Decomposition analysis showed that population growth was the major factor in GDSC burden changes, especially in high-SDI regions. By 2050, ASIR and ASMR are expected to continue declining, particularly for esophageal and gastric cancers. Major risk factors include smoking, alcohol, diet, and metabolic factors such as high body mass index (BMI) and elevated fasting glucose.

**Conclusion:**

Despite an overall decline in GDSC burden, health disparities persist, particularly between high- and low-SDI regions. The study provides valuable insights into risk factors and projections for disease burden and offers guidance for targeted prevention efforts.

## 1 Background

With the accelerating global aging process, the health burden among the older adults is increasingly severe, particularly as the incidence and mortality rates of cancer continue to rise annually (1). Among all types of cancer, geriatric digestive system cancers (GDSC), including esophageal, gastric, colorectal, liver, pancreatic, and bile duct cancers, hold a significant position in the population aged ≥60 years. According to the Global Cancer Epidemiology Report (GLOBOCAN) and other related studies, the disease burden of GDSC is progressively intensifying (2), which has garnered widespread attention in recent years concerning their epidemiology and etiology. The latest study from GBD 2021 included 371 diseases and injuries in 204 countries and regions and 88 risk factors to offer insights for developing public health strategies and effectively allocating resources worldwide (3). Our research, based on GBD, indicated that from 1990 to 2021, the proportion of digestive system cancers among all cancer types decreased in both the all-age group (27.96 to 22.30%) and the older adults group (30.22 to 23.70%), with a smaller decline observed in the older adults. This article conducts an epidemiological and risk factor analysis of GDSC, further elucidating the characteristics of gastrointestinal tumors in the older adults.

## 2 Methods

### 2.1 Data download and processing

Data for incidence, prevalence, mortality, disability-adjusted life years (DALYs), and risk factors were downloaded from the Global Health Data Exchange (GHDx),^1^ an online health data resource provided by the Institute for Health Metrics and Evaluation at the University of Washington. DALYs are defined as the sum of years of life lost (YLLs) due to premature death and years lived with disability (YLDs) (details in Supplementary material), from which a comprehensive assessment of disease burden can be derived. This study population included patients aged ≥60 years with six GDSC across 21 regions and 204 countries from 1990 to 2021. The socio-demographic index (SDI) was utilized as a composite measure to evaluate country-level factors, including the education level (aged >15 years), per capita income, and total fertility rate. With a scale ranging from 0 to 1, higher SDI values indicate more advanced socio-economic conditions. A total of 204 countries and territories were categorized based on the SDI quintile into the following five groups: low-SDI, low-middle-SDI, middle-SDI, high-middle-SDI, and high-SDI regions.

### 2.2 Case definition

Our study population comprised patients aged ≥60 years diagnosed with esophageal cancer (ICD-10 codes C15–C15.9), stomach cancer (ICD-10 codes C16–C16.9), colon and rectum cancers (ICD-10 codes C18, C19.0, C20, C21–C21.8), liver cancer (ICD-10 codes C22–C22.4, C22.7–C22.8), gallbladder and biliary tract cancer (GBTC) (ICD-10 codes C23, C24–C24.9), or pancreatic cancer (ICD-10 codes C25–C25.9). These diseases are classified under the International Classification of Diseases version 10 (ICD-10) system and represent major cancers of the gastrointestinal and hepato-pancreato-biliary systems.

### 2.3 Statistical analysis

To ensure comparability across regions with different age structures, the study data were age standardized using the GBD standard population and calculated by employing the direct age-standardization formula to obtain the age-standardized rate (ASR) for each age group, as follows: ASR = (*Σ* (*aᵢ* × *wᵢ*)/Σ *wᵢ*) × 100,000 (*aᵢ* is the disease-specific rate in the *i*-th age group, and *wᵢ* is the population weight for the i-th age group in the reference standard population) (4). Annual percentage change (APC) and its 95% confidence intervals (CIs) were computed using the joinpoint regression model to assess temporal trends over each independent time period (5). Temporal trends were analyzed by calculating the estimated annual percentage change (EAPC) for each ASR from 1990 to 2021 (6).

This study utilized the slope index of inequality (SII) and concentration index, as defined by the WHO, to assess absolute and relative inequality in disease burden (7). The SII was calculated by regressing the DALYs rate against the SDI, using the midpoint of the cumulative population distribution sorted by SDI. To examine changes in health inequality, we compared data from 204 countries and territories from 1990 to 2021, employing a robust regression model (rlm) to mitigate the effect of outliers and enhance accuracy. Compared to traditional linear regression analysis, robust regression reduces the effect of outliers on the regression results, making the regression coefficients more reliable. The concentration index was calculated by matching the cumulative DALYs proportion with the cumulative population distribution by SDI and numerically integrating the area under the Lorenz curve (8).

The Das Gupta decomposition method was employed to partition the changes in GDSC burden from 1990 to 2021 into contributions from population aging, growth, and epidemiological shifts (9). This approach enabled a detailed assessment of the independent contributions of each factor to the overall change in disease burden. The detailed statistical methods can be found in the Supplementary material.

To evaluate the relationship between GDSC burden and sociodemographic development, frontier analysis was applied to develop an age-standardized DALYs rate (ASDR)-based frontier model using the SDI. This approach identifies the theoretical minimum ASDR achievable by each country or territory based on its current level of development and serves serving as a benchmark for optimal performance (10). It quantifies the disparity between the current burden and the potential minimum, highlighting areas for improvement. The absolute distance (effective difference) between each country’s or territory’s 2021 ASDR and the frontier line was used to assess its improvement potential.

Furthermore, by utilizing the Bayesian age-period-cohort (BAPC) model, we predicted the future trends in incidence and mortality in the near future (11). The BAPC model is a powerful tool for understanding and forecasting disease trends by analyzing age, period, and cohort effects using Bayesian methods.

The population attributable fraction (PAF) was applied to estimate each risk factor’s contribution to the disease, which was calculated as follows: PAF = P * (RR − 1) / (1 + P * (RR − 1)), where, P is the exposure rate to the risk factor and RR is the relative risk (12). This analysis provides information about disease prevention strategies (GBD 2019 Risk Factors Collaborators, 2020).

In our research, the R program (version 4.1.3) was the main data statistical analysis software, with *p* < 0.05 indicating that the result had a statistical significance. The use of anonymized, publicly available epidemiological data did not require ethical approval, nor were patient-informed consent forms needed when accessing and downloading data from the database.

## 3 Results

### 3.1 Global epidemiology of GDSC

In 1990, the global incidence of GDSC was 1,876,969 (95% CI = 1,718,381–2,019,022), with an ASIR of 405.48/100,000 (69.19–436.45). In 2021, the incidence was 3,854,209 (3,376,252–4,272,039), with an ASIR of 360.89/100,000 (315.27–400.05), and the AAPC was −0.57 (95% CI = −0.80, −0.33). In 1990, the global mortality rate for GDSC was 1,619,349 (1,471,918–1,758,646), with an ASMR of 356.79/100,000 (322.52–387.52). In 2021, the mortality rate was 2,856,742 (2,491,799–3,175,587), with an ASMR of 270.14/100,000 (234.96, 300.30), and the AAPC was −1.72 (−1.81, −1.63). Globally, the burden of DALYs for GDSC showed a declining trend (AAPC = −1.42), with a more gradual decline from 1990 to 2004 (APC = −0.74), accelerated declines from 2004 to 2006 (APC = −2.51) and 2006 to 2015 (APC = −1.22), followed by a more gradual decline again from 2015 to 2021 (APC = −0.87) (Supplementary Figure S1A). Among the cancer types, gastric cancer exhibited the largest decrease in incidence (AAPC = −2.21 [−2.36, −2.05]), whereas liver cancer showed the most significant increase (AAPC = 0.74 [0.62, 0.87]). In terms of DALYs, the largest reduction was observed in gastric cancer (AAPC = −3.93 [−5.23, −2.62]), while pancreatic cancer demonstrated the largest increase (AAPC = 0.33 [−0.54, 1.20]). Regarding mortality, gastric cancer declined (AAPC = −3.64 [−3.71, −3.57]), and liver cancer increased the most (AAPC = 0.47 [0.34, 0.59]) (Table 1; Supplementary Figure S1).

**Table 1:** Global incident, prevalent, DALYs and death cases for Geriatric Digestive System Cancers in 1990 and 2021 and percentage change of age-standardized rates.

Global burden	Digestive system cancer	Number of cases, 1990 (95%UI)	Age-standardized rate per 100,000 population, 1990 (95%UI)	Number of cases, 2021 (95%UI)	Age-standardized rate per 100,000 population, 2021 (95%UI)	EAPC, 1990–2021 (95%CI)	AAPC (95%CI)
Incidence	Total	1,876,969 (1,718,381, 2,019,022)	405.48 (369.19, 436.45)	3,854,209 (3,376,252, 4,272,039)	360.89 (315.27, 400.05)	−0.42 (−0.46, −0.38)	−0.57 (−0.80, −0.33)
	Esophageal cancer	226,411 (198,851, 249,655)	47.16 (41.39, 51.94)	421,336 (365,896, 478,329)	39.08 (33.91, 44.36)	−0.75 (−0.86, −0.63)	−0.95 (−1.07, −0.84)
	Stomach cancer	630,551 (572,742, 691,697)	134.56 (121.86, 147.56)	896,397 (757,409, 1,025,419)	83.82 (70.74, 95.79)	−1.55 (−1.61, −1.49)	−2.21 (−2.36, −2.05)
	Colon and rectum cancer	655,024 (611,558, 683,892)	144.74 (133.81, 151.59)	1,617,011 (1,447,212, 1,754,348)	151.61 (135.19, 164.60)	0.09 (0.05, 0.14)	0.22 (−0.08, 0.53)
	Liver cancer	130,055 (118,733, 143,308)	27.05 (24.62, 29.85)	341,395 (304,410, 378,506)	31.78 (28.26, 35.23)	0.40 (0.27, 0.54)	0.74 (0.62, 0.87)
	Pancreatic cancer	152,251 (142,395, 159,736)	33.50 (31.05, 35.25)	403,853 (358,257, 437,883)	38.05 (33.61, 41.30)	0.49 (0.46, 0.53)	0.63 (0.57, 0.68)
	GBTC	82,677 (74,102, 90,734)	18.47 (16.47, 20.26)	174,216 (143,068, 197,555)	16.55 (13.57, 18.78)	−0.41 (−0.44, −0.37)	−0.58 (−0.60, −0.57)
Prevalence	Total	4,449,183 (4,128,103, 4,763,134)	939.55 (867.41, 1009.98)	11,282,396 (10,092,703, 12,349,565)	1043.24 (931.55, 1142.11)	0.36 (0.30, 0.43)	0.54 (0.12, 0.95)
	Esophageal cancer	299,849 (263,952, 329,805)	60.65 (53.37, 66.65)	655,313 (573,097, 740,831)	59.97 (52.42, 67.79)	−0.09 (−0.22, 0.04)	−0.06 (−0.20, 0.07)
	Stomach cancer	939,500 (862,455, 1,015,822)	193.97 (177.66, 209.72)	1,568,205 (1,330,519, 1,797,897)	144.51 (122.53, 165.54)	−0.91 (−1.00, −0.82)	−1.30 (−1.53, −1.07)
	Colon and rectum cancer	2,866,157 (2,684,899, 3,047,785)	612.23 (569.71, 655.33)	8,079,970 (7,332,761, 8,727,386)	747.36 (676.91, 807.55)	0.67 (0.62, 0.73)	1.02 (0.85, 1.20)
	Liver cancer	136,181 (125,309, 149,340)	27.74 (25.46, 30.44)	417,259 (372,090, 463,352)	38.63 (34.35, 42.92)	0.98 (0.78, 1.19)	1.48 (1.36, 1.60)
	Pancreatic cancer	112,307 (105,377, 117,626)	24.18 (22.51, 25.39)	319,701 (283,635, 346,991)	29.96 (26.46, 32.56)	0.84 (0.79, 0.90)	1.34 (1.33, 1.36)
	GBTC	95,187 (86,111, 102,756)	20.79 (18.71, 22.45)	241,947 (200,600, 273,107)	22.80 (18.88, 25.74)	0.30 (0.25, 0.35)	0.42 (0.39, 0.46)
DALYs	Total	32,932,702 (30,031,872, 35,795,486)	6838.74 (6219.84, 7433.08)	53,939,966 (47,584,531, 59,978,277)	4980.95 (4386.30, 5538.14)	−1.10 (−1.15, −1.05)	−1.42 (−12.79, 9.95)
	Esophageal cancer	5,123,634 (4,497,405, 5,665,726)	1036.26 (909.30, 1144.96)	8,059,156 (7,029,414, 9,163,785)	738.20 (643.58, 839.21)	−1.25 (−1.37, −1.13)	−1.64 (−4.22, 0.94)
	Stomach cancer	11,928,996 (10,784,197, 13,214,086)	2456.62 (2218.72, 2720.79)	13,797,102 (11,753,648, 15,790,412)	1272.06 (1082.71, 1454.91)	−2.19 (−2.27, −2.11)	−3.93 (−5.23, −2.62)
	Colon and rectum cancer	8,303,284 (7,773,030, 8,709,793)	1777.10 (1652.18, 1867.51)	15,347,705 (13,913,323, 16,592,781)	1427.86 (1290.41, 1544.63)	−0.79 (−0.82, −0.75)	−0.77 (−12.00, 10.47)
	Liver cancer	2,925,891 (2,672,345, 3,229,636)	590.70 (538.50, 652.61)	6,538,211 (5,884,148, 7,257,231)	599.59 (538.71, 665.49)	−0.06 (−0.19, 0.07)	0.21 (−2.95, 3.36)
	Pancreatic cancer	3,157,368 (2,975,127, 3,308,708)	661.37 (619.76, 694.28)	7,643,080 (6,912,849, 8,256,300)	705.73 (636.52, 762.75)	0.27 (0.23, 0.30)	0.33 (−0.54, 1.20)
	GBTC	1,493,528 (1,329,769, 1,667,537)	316.69 (281.38, 352.94)	2,554,711 (2,091,149, 2,917,769)	237.51 (194.37, 271.15)	−1.02 (−1.05, −0.98)	−1.72 (−1.81, −1.63)
Deaths	Total	1,619,349 (1,471,918, 1,758,646)	356.79 (322.52, 387.52)	2,856,742 (2,491,799, 3,175,587)	270.14 (234.96, 300.30)	−0.96 (−1.01, −0.91)	−1.26 (−1.75, −0.76)
	Esophageal cancer	238,854 (209,539, 263,324)	50.43 (44.21, 55.53)	410,902 (356,634, 466,427)	38.37 (33.28, 43.55)	−1.06 (−1.19, −0.92)	−1.35 (−1.49, −1.21)
	Stomach cancer	580,146 (524,548, 642,458)	126.09 (113.66, 139.54)	732,426 (620,849, 834,822)	69.10 (58.48, 78.69)	−2.00 (−2.08, −1.92)	−3.64 (−3.71, −3.57)
	Colon and rectum cancer	426,624 (395,396, 448,106)	98.31 (90.04, 103.60)	827,471 (736,233, 897,443)	79.10 (70.05, 85.87)	−0.77 (−0.81, −0.74)	−0.76 (−1.34, −0.18)
	Liver cancer	135,915 (123,735, 150,355)	28.68 (26.01, 31.77)	332,455 (296,105, 368,772)	31.12 (27.64, 34.52)	0.18 (0.06, 0.31)	0.47 (0.34, 0.59)
	Pancreatic cancer	160,121 (149,585, 168,193)	35.70 (33.03, 37.62)	411,533 (365,890, 446,353)	38.91 (34.44, 42.24)	0.34 (0.31, 0.37)	0.43 (0.38, 0.47)
	GBTC	77,689 (69,116, 86,208)	17.58 (15.57, 19.48)	141,955 (116,087, 161,769)	13.55 (11.07, 15.44)	−0.92 (−0.95, −0.89)	−1.56 (−1.56, −1.55)

DALYs, disability-adjusted life year; ASR, age standardized rate; ASIR, age-standardized incidence rate; ASDR, age-standardized DALYs rate; ASMR, age-standardized death rate; ASPR, age-standardized prevalence rate; EAPC, estimated annual percentage change; CI, confidence interval; UI, uncertainty interval; AAPC, Average Annual Percent Change; GBTC, Gallbladder and biliary tract cancer.

**Figure 1:**
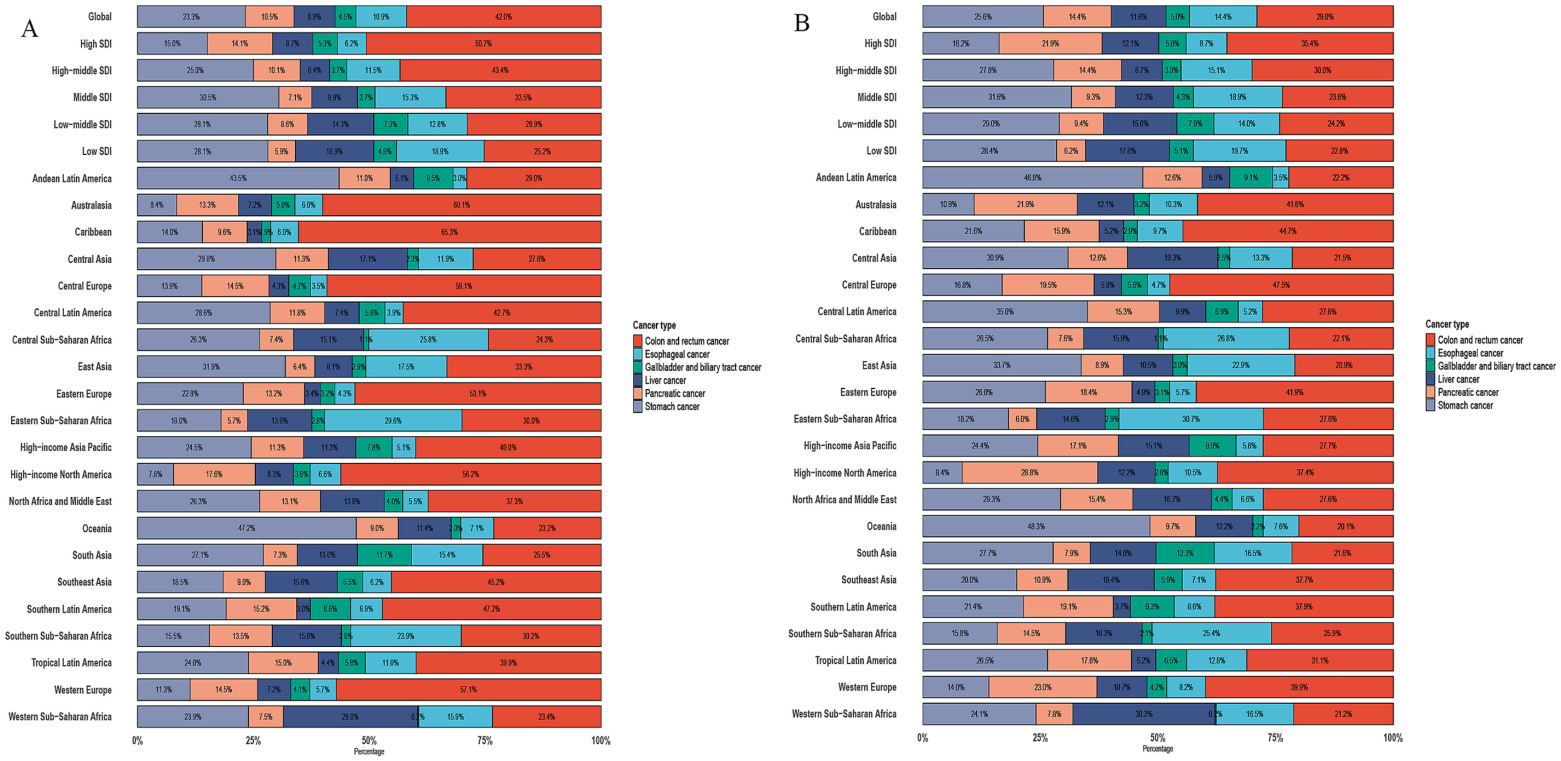
Proportion of incidence and death due to six GDSC in 2021. **(A)** Proportion of Incidence due to six GDSC in different SDI locations and GBD regions in 2021. **(B)** Proportion of Death due to six GDSC in different SDI locations and GBD regions in 2021.

We conducted an analysis of cancer types among patients aged ≥60 years and found that GDSC ranks prominently in both incidence and mortality. Globally, esophageal cancer ranks 9th in incidence and 6th in mortality; stomach cancer, 7th in incidence and 3rd in mortality; colorectal cancer, 4th in incidence and 2nd in mortality; liver cancer, 12th in incidence and 8th in mortality; pancreatic cancer, 10th in incidence and 5th in mortality; and GBTC, 20th in incidence and 13th in mortality (Supplementary Figure S2). We further analyzed the proportions of GDSC, which indicates that colorectal cancer has the highest proportion in both incidence and mortality (42, 29%), followed by gastric cancer (23.3, 25.6%), esophageal cancer (10.9, 14.4%), pancreatic cancer (10.5, 14.4%), liver cancer (8.9, 11.6%), and GBTC (4.5, 5.0%). The disease proportion varies greatly across different regions and SDI levels (Figure 1).

**Figure 2:**
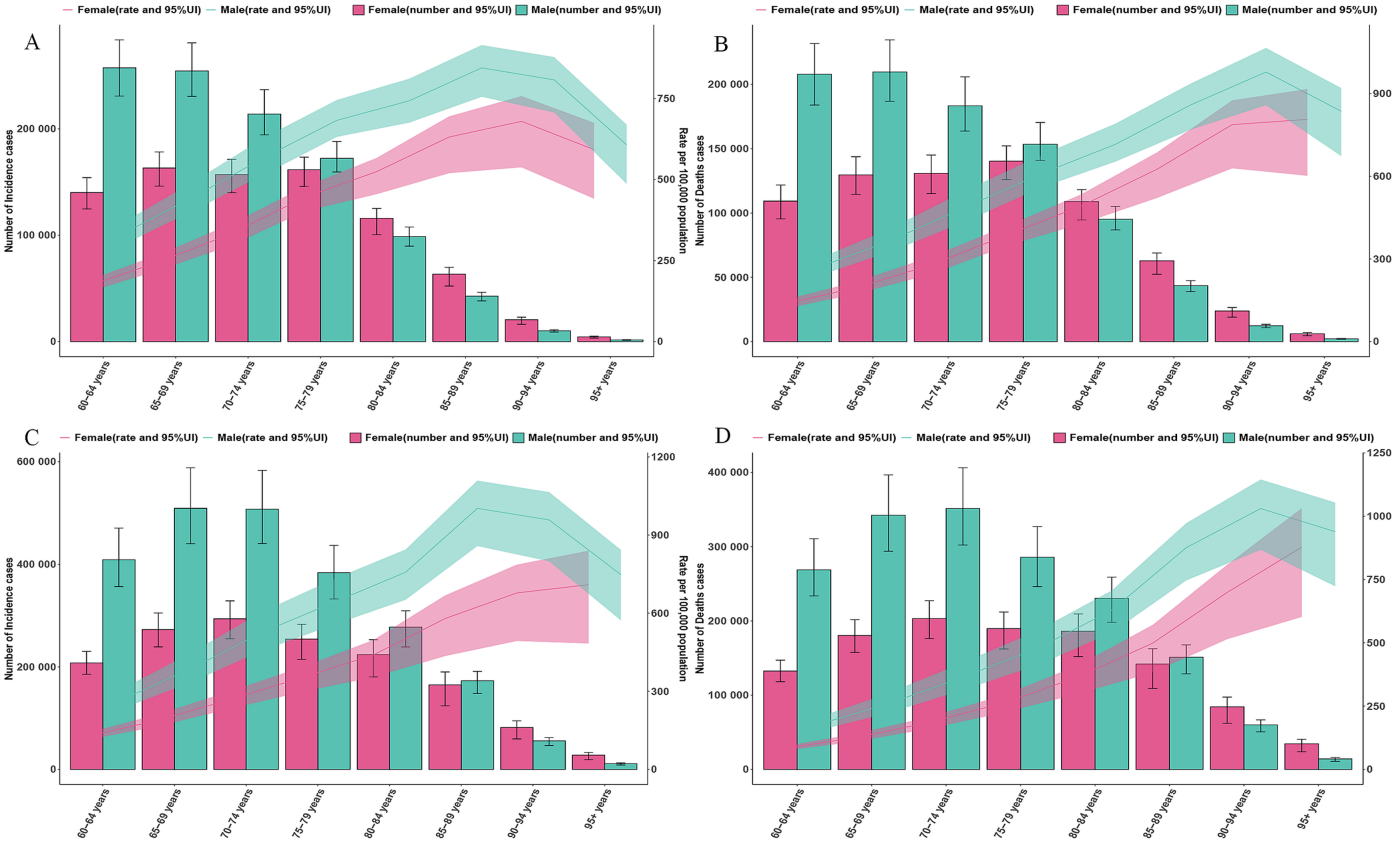
Global counts and incidence, mortality rates of GDSC by age and sex, 1990 and 2021. **(A)** Global counts and incidence rates of GDSC by age and sex in 1990. **(B)** Global counts and mortality rates of GDSC by age and sex in 1990. **(C)** Global counts and incidence rates of GDSC by age and sex in 2021. **(D)** Global counts and mortality rates of GDSC by age and sex in 2021. Error bars indicate the 95% uncertainty intervals (95% UI) for incidence, death. Shading indicates the upper and lower limits of the 95% UI.

As shown in Figure 2, gender and age analysis reveals that the incidence and mortality rates for males are higher than those for females in GDSC. The male incidence and death peak shifted from 60–69 years in 1990 to 65–74 years in 2021. The female incidence and death peak shifted from 65–79 years in 1990 to 70–74 years in 2021. Comparing the changes in ASR, the ASIR and ASMR for those aged 80–84 years in 2021 are lower than the levels in 1990, while the ASIR and ASMR for those aged ≥90 years are higher than the levels in 1990 (Figure 2).

We analyzed the age–gender distribution of six types of GDSC and found that the highest number of incidences occurred in the 60–79 age group, while the highest number of deaths occurred in the 65–74 age group. In terms of gender distribution, the disease burden was significantly higher in males for esophageal, gastric, and liver cancers, whereas the gender distribution was more balanced for colorectal and pancreatic cancers. Among individuals aged ≥80 years, the incidence and mortality rates for pancreatic cancer were higher in females than in males, and for GBTC, the disease burden in females was also slightly higher than in males (Supplementary Figure S3).

### 3.2 GDSC by regions and countries

**Figure 3:**
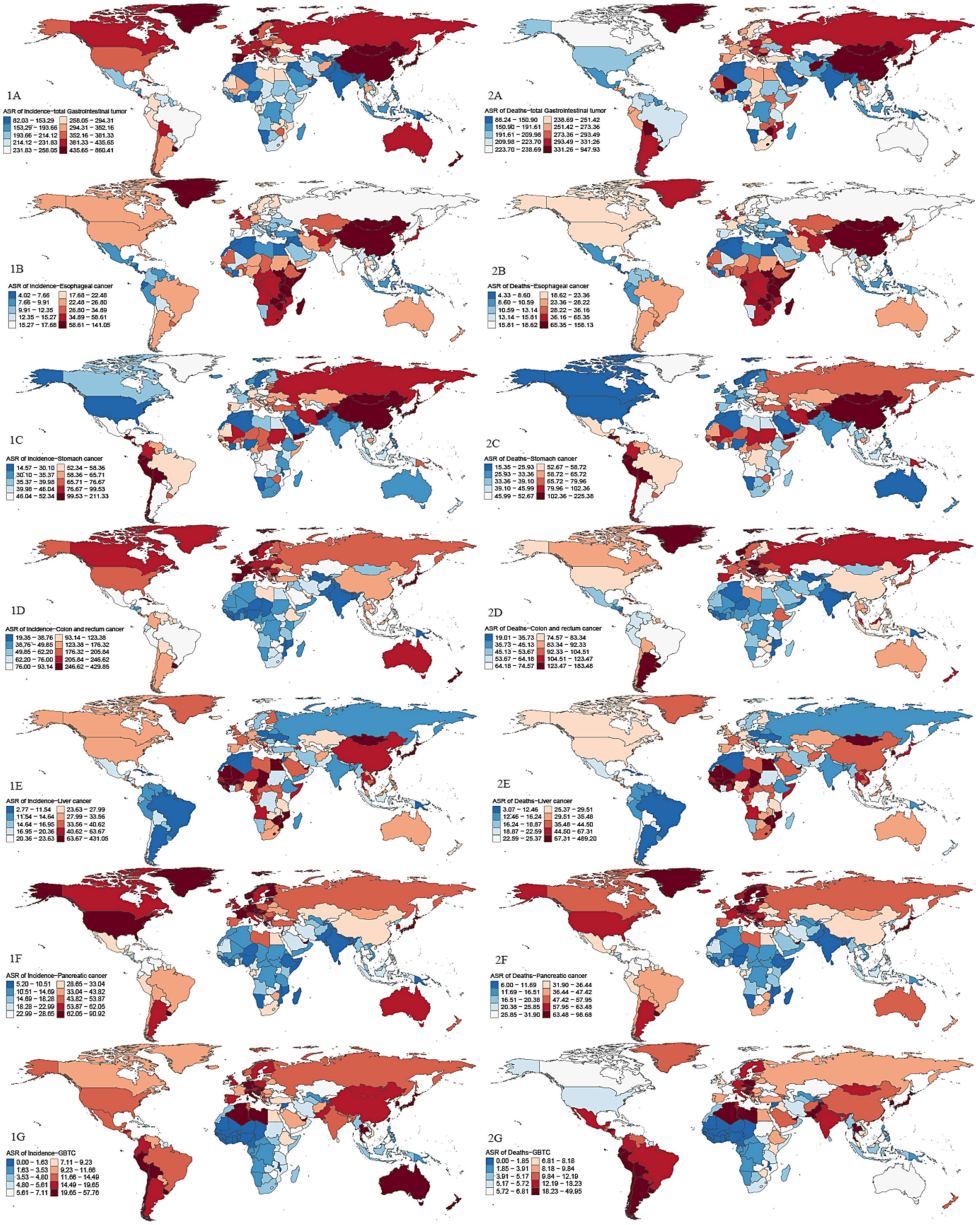
Global maps of ASIR and ASMR in 2021 for GDSC. Darker red shades indicate higher ASRs, while blue shades represent lower ASRs. 1A: ASIR for GDSC; 2A: ASMR for GDSC; 1B: ASIR for Geriatric Esophageal Cancer; 2B: ASMR for Geriatric Esophageal Cancer; 1C: ASIR for Geriatric Gastric Cancer; 2C:ASMR for Geriatric Gastric Cancer; 1D:ASIR for Geriatric Colorectal Cancer; 2D:ASMR for Geriatric Colorectal Cancer; 1E:ASIR for Geriatric Liver Cancer; 2E:ASMR for Geriatric Liver Cancer; 1F:ASIR for Geriatric Pancreatic Cancer; 2F:ASMR for Geriatric Pancreatic Cancer; 1G:ASIR for Geriatric GBTC; 2G:ASMR for Geriatric GBTC. Top 3 regions and countries with the highest age-standardized incidence rates (ASIR) and mortality rates (ASMR) for six cancer types based on GDSC data. Esophageal cancer: ASIR: East Asia, Eastern Sub-Saharan Africa, Southern Sub-Saharan Africa; Top countries: Malawi, Mongolia, China. ASMR: East Asia, Eastern Sub-Saharan Africa, Southern Sub-Saharan Africa; Top countries: Malawi, Mongolia, Zambia. Gastric cancer: ASIR: East Asia, High-income Asia Pacific, Andean Latin America; Top countries: Mongolia, Bolivia, Afghanistan. ASMR: High-income Asia Pacific, Western Sub-Saharan Africa, Southern Sub-Saharan Africa; Top countries: Mongolia, Bolivia, Afghanistan. Colorectal cancer: ASIR: High-income Asia Pacific, Australasia, Western Europe; Top countries: Netherlands, Monaco, Bermuda. ASMR: Central Europe, Southern Latin America, Eastern Europe; Top countries: Uruguay, Monaco, Hungary. Liver cancer: ASIR: High-income Asia Pacific, Western Sub-Saharan Africa, East Asia; Top countries: Mongolia, Gambia, Mali. ASMR: High-income Asia Pacific, Western Sub-Saharan Africa, Southern Sub-Saharan Africa; Top countries: Mongolia, Gambia, Mali. Pancreatic cancer: ASIR: High-income Asia Pacific, North America, Western Europe; Top countries: Greenland, Monaco, Finland. ASMR: High-income Asia Pacific, Western Europe, Central Europe; Top countries: Greenland, Monaco, Uruguay. GBTC: ASIR: High-income Asia Pacific, Southern Latin America, Andean Latin America; Top countries: Republic of Korea, Chile, Japan. ASMR: High-income Asia Pacific, Southern Latin America, Andean Latin America; Top countries: Chile, Republic of Korea, Thailand.

In terms of regional analysis, the top three ASIR regions of GDSC in 2021 were high-income Asia-Pacific (656.57/100,000 [558.67–732.23]), East Asia (515.88/100,000 [406.80–630.19]), and Western Europe (427.21/100,000 [378.83, −462.98]). The top three ASMR regions were East Asia (393.98/100,000 [310.53–481.13]), high-income Asia-Pacific (368.79/100,000 [311.83–408.66]), and Central Europe (314.74/100,000 [284.91–341.82]). The top three AAPC regions for incidence were Southern Sub-Saharan Africa (1 [0.20, 1.80]), Southeast Asia (0.49 [−0.18, 1.16]), and the Caribbean (0.46 [−2.74, 3.66]). The top three AAPC regions for mortality were Southern Sub-Saharan Africa (0.87 [−0.10, 1.84]), Western Sub-Saharan Africa (0.14 [0.04, 0.25]), and South Asia (0.04 [−0.09, 0.18]). The countries with the highest ASIR were Mongolia, Japan, and Monaco, and the countries with the highest ASMR were Mongolia, Cabo Verde, and Bolivia (Plurinational State of) (Supplementary Table S1; Figure 3).

In 2021, the regions with the highest ASIR for geriatric esophageal cancer were East Asia, Eastern Sub-Saharan Africa, and Southern Sub-Saharan Africa. For geriatric gastric cancer, East Asia had the highest ASIR at 164.30/100,000 (125.74–203.94), followed by high-income Asia-Pacific and Andean Latin America. The regions with the highest ASIR for geriatric colorectal cancer were high-income Asia-Pacific, Australasia, and Western Europe. The regions with the highest ASIR for geriatric liver cancer were high-income Asia-Pacific, Western Sub-Saharan Africa, and East Asia. For geriatric pancreatic cancer, high-income Asia-Pacific had the highest ASIR, followed by high-income North America and Western Europe. Finally, for geriatric GBTC, the highest ASIR was seen in high-income Asia-Pacific, Southern Latin America, and Andean Latin America.

### 3.3 Decomposition and frontier analysis of incidence, mortality, and DALYs rates

**Figure 4:**
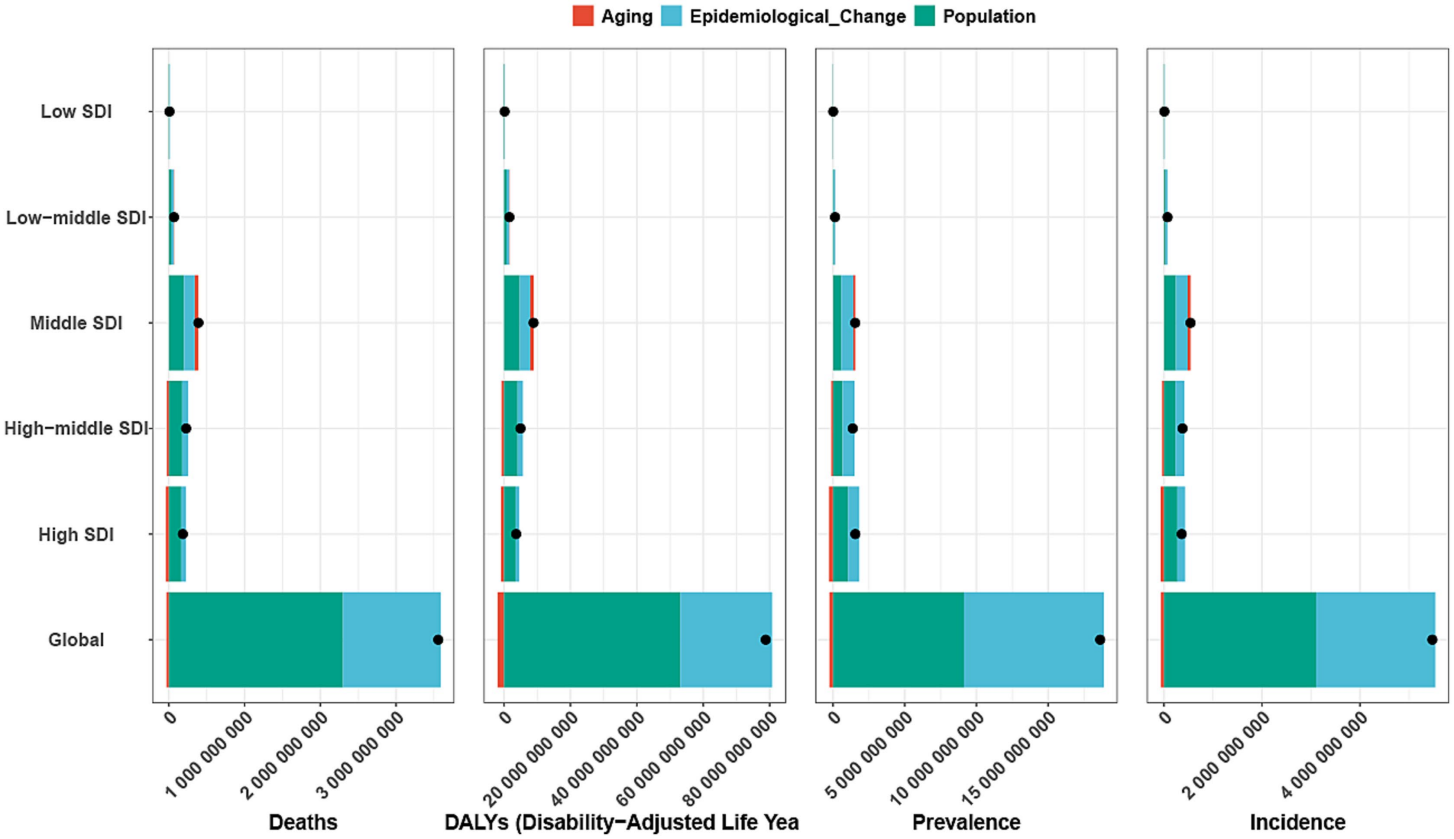
Decomposition analysis of incidence, mortality, prevalence and DALYs. A black dot indicates the overall change induced by the three components (population growth, aging, and epidemiological alteration). For each component, an increase in the incidence, mortality, and DALYs of Geriatric Digestive System Cancer associated with that component is represented by a positive value. A decrease in the incidence, mortality, and DALYs of associated with that component is represented by a negative value. ASR, age-standardized rate; DALYs, disability adjusted life-years; SDI, sociodemographic index.

Decomposition analysis indicates that over the past 30 years, the global burden of GDSC has undergone significant changes, with the most notable shifts observed in the high-middle- and high-SDI quintile regions (Figure 4). On a global scale, population growth was the primary factor driving changes in deaths (64.65%), DALYs (67.32%), prevalence (49.01%), and incidence (56.69%). This trend was evident across all SDI quintile regions, with particularly pronounced changes in incidence and prevalence in the high-middle- and high-SDI regions. In contrast to population growth, aging contributed less to the changes in deaths and DALYs globally, accounting for −0.89 and −2.48%, respectively. Unlike deaths and DALYs, changes in prevalence and incidence were more influenced by epidemiological changes, which were the dominant factor in all regions, particularly in the high-SDI and high-middle-SDI regions. Epidemiological change accounted for 50.81 and 59.53% of the changes in prevalence, and 43.22 and 47.56% of the changes in incidence, respectively (Figure 4; Supplementary Table S2).

**Figure 5:**
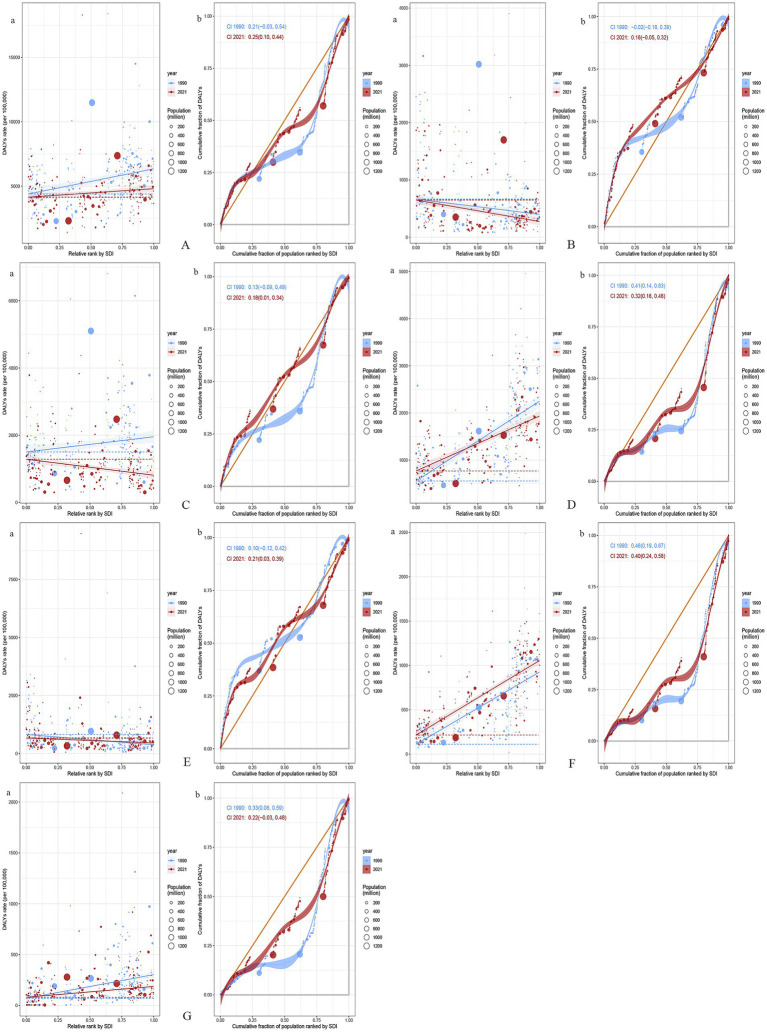
Health inequality regression curves and concentration curves for the DALYs of GDSC **(A)**, Geriatric Esophageal Cancer **(B)**, Geriatric Gastric Cancer **(C)**, Geriatric Colorectal Cancer **(D)**, Geriatric Liver Cancer **(E)**, Geriatric Pancreatic Cancer **(F)** and Geriatric GBTC **(G)** worldwide, 1990 and 2021. Panel **(a)** illustrate the slope index of inequality, depicting the relationship between SDI and age-standardized DALYs rates for each condition, with points representing individual countries and territories sized by population. Panel **(b)** present the concentration index, which quantifies relative inequalities by integrating the area under the Lorenz curve, aligning DALYs distribution with population distribution by SDI. Blue represents data from 1990, and red represents data from 2021. DALYs, disability-adjusted life-years; GDSC, Geriatric Digestive System Cancers; GBTC, gallbladder and biliary tract cancer; SDI, sociodemographic index.

We observed significant absolute and relative inequalities for GDSC associated with the SDI, with countries and territories having higher SDI disproportionately bearing a greater burden. As shown by the SII, the gap in the DALYs rate between the highest and lowest SDI countries and territories decreased from 1956.10/100,000 (1247.89–2664.32) in 1990 to 658.41/100,000 (136.08–1180.74) in 2021. The concentration index was 0.21 (−0.03 to 0.54) in 1990 but increased to 0.25 (0.10 to 0.44) in 2021. These results suggest that while absolute health inequalities in GDSC burden decreased from 1990 to 2021, relative inequalities have increased (Figure 5).

From 1990 to 2021, global GDSC burden trends showed significant changes in absolute and relative inequalities. For esophageal cancer, the DALYs gap between high- and low-SDI regions increased from −266.24/100,000 (−425.79, −106.69) to −374.34/100,000 (−498.35, −250.34) as well as relative inequality (concentration index from −0.02 to 0.16). Gastric cancer showed a narrowing absolute gap from 30.10/100,000 (12.26, 47.94) to −22.18/100,000 (−32.87, −11.48) but an increase in relative inequality (concentration index from 0.13 to 0.18). Colorectal cancer showed a decrease in both absolute disparity from 1692.24/100,000 (1426.65, 1957.82) to 1164.77/100,000 (969.39, 1360.14) and relative inequality from 0.41 to 0.32. Liver cancer had a reduced absolute gap from −442.80/100,000 (−610.05, −275.55) to −208.23/100,000 (−337.51, −78.95), but its relative inequality worsened (from 0.10 to 0.21). Pancreatic cancer exhibited an increase in the absolute gap from 815.48/100,000 (708.17, 922.80) to 839.84/100,000 (740.40, 939.27), although its relative inequality decreased (from 0.46 to 0.40). For GBTC, the absolute gap shrank from 233.50/100,000 (178.26, 288.75) to 106.30/100,000 (68.63, 143.97) as well as the relative inequality (from 0.33 to 0.22). Overall, while absolute disparities in cancer burden narrowed in some areas, relative inequalities persisted or increased, particularly in low-SDI countries (Figure 5).

**Figure 6:**
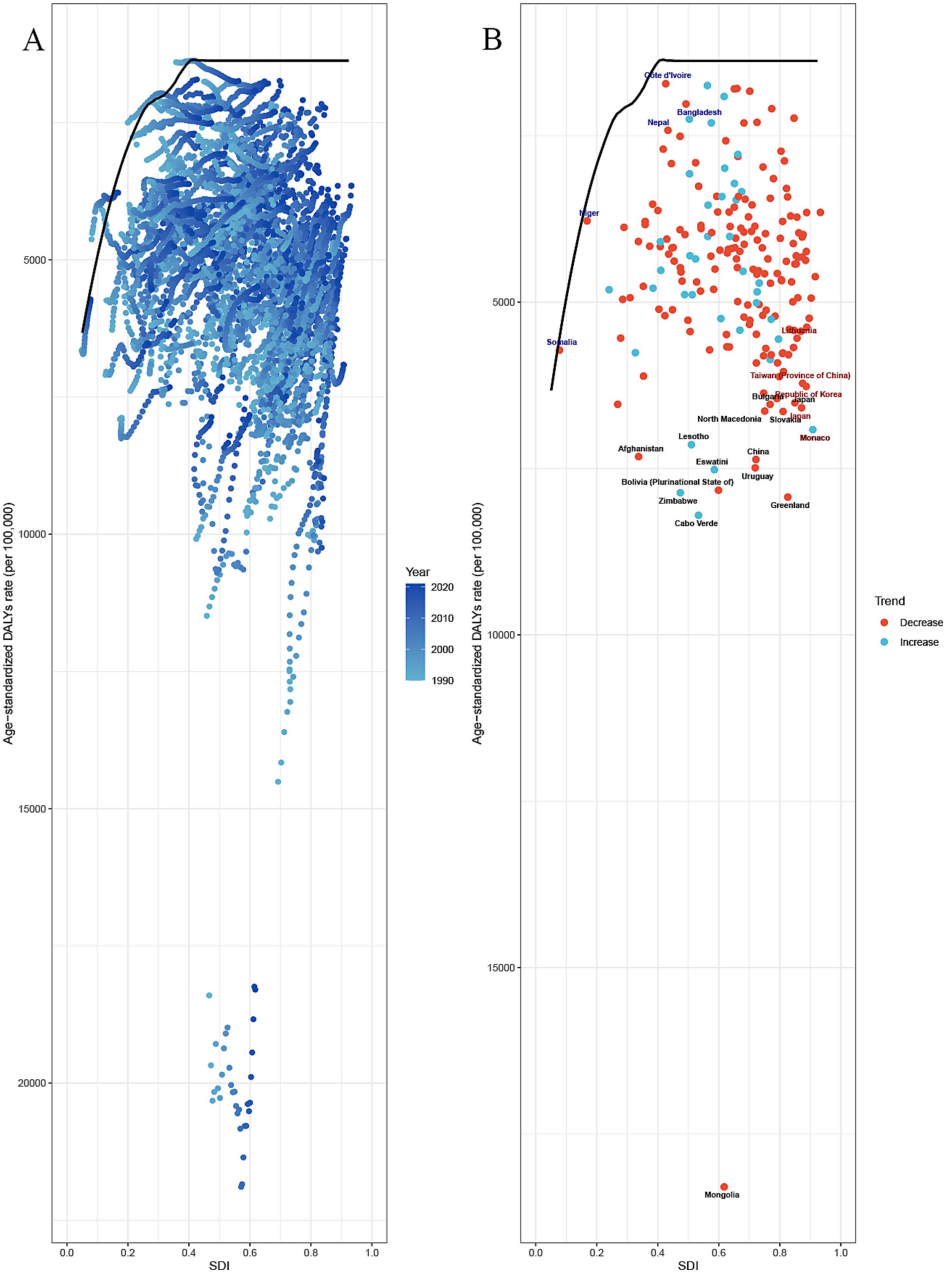
Frontier analysis of SDI and the GDSC burden in 2021. **(A,B)** Frontier analysis for age–standardized rate of DALYs. Solid black delineates the frontier, and dots represent countries and territories. Black marks the top 15 countries with the largest effective difference (the highest ASDR of the elder with an DSC gap from the frontier). Blue indicates frontier countries with a low SDI (<0.5) and a low effective difference (e.g., Niger, Côte d’Ivoire, Bangladesh, Nepal, Bhutan). Red indicates countries and territories with a high SDI (>0.85) and a relatively high effective difference for their development level (e.g., Monaco, Japan). An enhanced age-standardized rate between 1990 and 2021 is indicated using a red dot. A reduced age-standardized rate between 1990 and 2021 is indicated using a blue dot. GDSC, Geriatric Digestive System Cancers; DALYs, disability adjusted life-years; SD, sociodemographic index.

The frontier analysis provides an insight into which countries are closest to or furthest from the ideal situation in managing the burden of GDSC (specifically DALYs). The 15 countries and territories with the largest actual differences in potential improvement (effective difference range: 16924.82–5162.23) include Mongolia, Cabo Verde, Greenland, Zimbabwe, Bolivia, Eswatini, Uruguay, China, Lesotho, Monaco, Afghanistan, Slovakia, North Macedonia, Japan, and Bulgaria. Considering their development level, high-SDI countries with relatively high improvement potential include Greenland, Monaco, Slovak, and Japan. The analysis indicates that low-SDI countries, such as Niger, Côte d’Ivoire, Bangladesh, Nepal, and Bhutan, have managed to control disease burden relatively well despite their limited resources (Figure 6). Using the same analysis, we identified the high-SDI countries that have the highest improvement potential for DALYs in different GDSC: colorectal cancer (Slovakia, Monaco, Poland, Greenland, Netherlands, Czechia, and Taiwan [Province of China]), liver cancer (Qatar), pancreatic cancer (Greenland, United Arab Emirates, Czechia, Finland, Germany, Denmark, Latvia, Estonia, and Austria), and GBTC (Republic of Korea, United Arab Emirates, Japan, Slovakia, and Czechia) (Supplementary Figure S4; Supplementary Table S3).

### 3.4 GDSC projection to 2050

**Figure 7:**
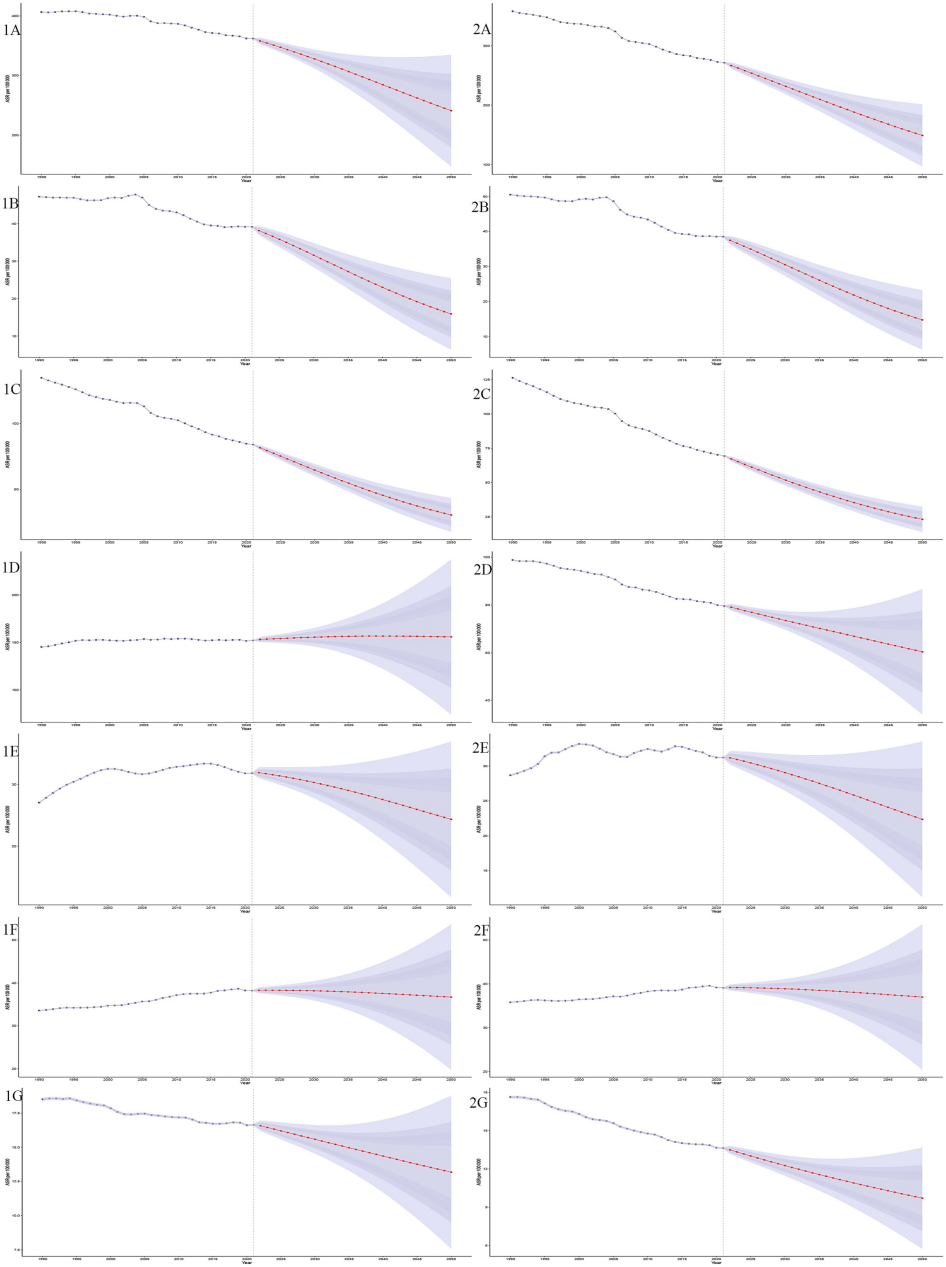
Projections of age-standardized incidence and mortality rates (ASIR and ASMR) for GDSC from 1990 to 2050. This figure illustrates the trends and future projections of ASIR and age-standardized mortality rates ASMR per 100,000 population for GDSC globally, from 1990 to 2050. The shaded ribbons represent the 95% confidence intervals (CIs) of the projections, providing an estimate of uncertainty. The projections demonstrate distinct trends for each disease, ASIR projections (1A–1G): GDSC (1A), Geriatric Esophageal Cancer (1B), Geriatric Gastric Cancer (1C), Geriatric Colorectal Cancer (1D), Geriatric Liver Cancer (1E), Geriatric Pancreatic Cancer (1F) and Geriatric GBTC (1G); ASMR projections (2A–2G): GDSC (2A), Geriatric Esophageal Cancer (2B), Geriatric Gastric Cancer (2C), Geriatric Colorectal Cancer (2D), Geriatric Liver Cancer (2E), Geriatric Pancreatic Cancer (2F), and Geriatric GBTC (2G).

Bayesian age-period-cohort models were used to predict ASIR and ASMR from 2020 to 2050. Both ASIR and ASMR are projected to decline, with ASMR decreasing faster than ASIR for GDSC. By 2050, ASIR will be (241.02 ± 47.79)/100,000, and ASMR will be (148.97 ± 26.71)/100,000. The predicted incidence is 5,183,288 ± 1,026,932, and mortality is 3,203,769 ± 574,377. The incidence for pancreatic and colorectal cancers will remain stable, but ASMR for colorectal cancer will decline faster than its incidence. Mortality and ASMR for pancreatic cancer will show minimal change. Both ASIR and ASMR of liver cancer and GBTC will decline slowly, while esophageal and gastric cancers will continue to decrease (Figure 7).

### 3.5 Risk factors

**Figure 8:**
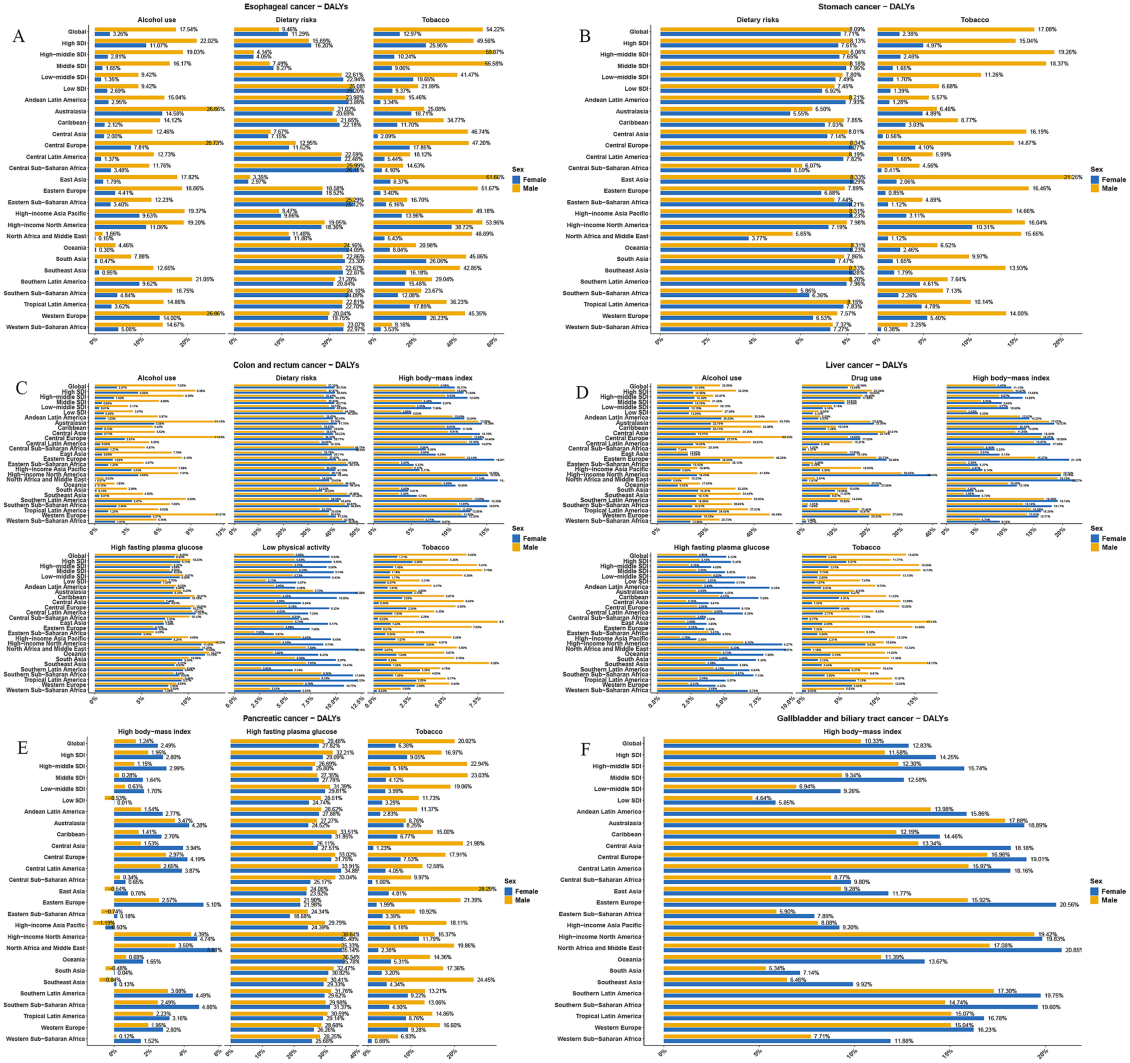
Percentage contribution of risk factors to DALYs for GDSC in 2021 by global and regional distribution. This figure illustrates the percentage contributions of key risk factors to the DALYs for GDSC, stratified by 21 global regions and both sexes in 2021. Each bar represents the proportion of DALYs attributable to a specific risk factor in the respective region. Regions are listed along the y-axis, with values presented as percentages on the x-axis. **(A)** Geriatric Esophageal Cancer, **(B)** Geriatric Gastric Cancer, **(C)** Geriatric Colorectal Cancer, **(D)** Geriatric Liver Cancer, **(E)** Geriatric Pancreatic Cancer, and **(F)** Geriatric GBTC.

Based on analysis of the global burden of disease (DALYs) attributable to risk factors in individuals aged over 60, key contributors to GDSC have been identified. Significant risk factors for esophageal cancer include tobacco use (42.9%), alcohol use (13.7%), and dietary risks (low vegetable intake, 9.92%). In stomach cancer, tobacco use accounts for 12.1% and high sodium intake, 7.96%. Liver cancer shows a strong association with alcohol use (21.7%), drug use (15.1%), metabolic risks (high fasting plasma glucose, 3.75%; high BMI, 9.43%), low physical activity (7.38%), and tobacco use (9.96%). For colon and rectum cancer, tobacco use contributes 4.58% and alcohol use, 5.24%, and includes metabolic risks (high fasting plasma glucose, 8.74%, and high BMI, 9.56%) and the dietary risks (38.8%) of low whole-grain intake (17.9%), low milk consumption (14.8%), high red meat (14.8%) and processed meat intake (5.58%), and low fiber (1.14%) and inadequate calcium intake (8.15%), all of which highlight the multifaceted nature of their etiology. For GBTC, metabolic risks (high BMI) contribute 11.7%. Pancreatic cancer is associated with metabolic risks (high fasting plasma glucose, 28.7%, and high BMI, 1.83%) and tobacco use (13.6%). This comprehensive assessment underscores the complex interplay between behavioral, metabolic, and dietary factors in driving the disease burden of GDSC globally. Additionally, we analyzed the effect of risk factors on GDSC across different regions and genders, which provides valuable insights into how regional and gender-based variations influence GDSC, thereby informing targeted prevention and treatment strategies (Figure 8).

## 4 Discussion

Older adults tend to be more affected by risk factors for developing cancer compared to younger individuals. The causes include long-term inflammation at a severe level, aging cells, genetic predisposition, weakened immune system function, and alterations in the environment where cancers develop, which, collectively, contribute to the development and progression of GDSC.

Our research indicated that the rates of incidences and deaths related to GDSC have decreased from 1990 to 2021, along with a reduction in the burden of DALYs. However, analysis of the data revealed variations in trends among six types of GDSC with differences in how they are changing over time. Specifically, ASIR and ASMR of stomach cancer have seen the most significant drop, whereas liver cancer has notably increased. This information is consistent with previous research findings, where the reduction in gastric cancer burden may be related to improvements in dietary habits, a decrease in *Helicobacter pylori* infection, and the enhancement of early screening methods that increase early diagnosis rates. Contrarily, the number of liver cancer cases has notably increased, with an annual percentage change (AAPC) of 0. 74. This increase is likely tied to hepatitis B and C infections, high levels of alcohol consumption and a rising occurrence of non-alcoholic fatty liver disease due to metabolic syndrome and obesity. The rise of pancreatic cancer stands out with the highest increase in DALYs rates at 0.33, indicating the challenges in detecting and treating this aggressive form of cancer. It continues to pose significant obstacles with a grim outlook underscoring the necessity, for improved therapies and early detection techniques. Colorectal cancer accounts for a significant share of GDSC cases and deaths at 42 and 29%, respectively, underscoring its importance among older individuals. Its substantial effect is likely associated with dietary patterns, changes in gut bacteria composition, genetic factors and delayed detection in the older adults community.

The research also discovered that the highest incidence of GDSC was seen in individuals aged 60–79 years old and the highest mortality was noted in the 65 to 74 age group. Data from GLOBOCAN indicates a rising trend in the occurrence and mortality rates of gastric cancers among individuals aged ≥80 years compared to their incidence and mortality in 1990, where it was shown that both the occurrence and death rates have decreased. Such a decrease could be attributed to longer life expectancies in the population and better access to early medical care along with improvements in living standards and medical advancements that are easing the effect of gastrointestinal cancers to some degree. In this study, we also identified gender differences in the disease burden. The incidence in gastric and liver cancers in males is generally higher than that in females, a fact that is often associated with consistent exposure to high-risk factors among males. Additionally, it is noted that in the age group ≥80 years, pancreatic cancer tends to affect women more than men, which could be attributed to biological traits unique to older women along with hormonal shifts and the hidden progression of pancreatic cancer (13). GBTC is often associated with chronic biliary diseases and lifestyle factors such as high-fat diets, and females may face a greater disease burden due to physiological factors, such as a higher incidence of cholelithiasis.

We conducted a decomposition analysis of the changes in the global burden of GDSC over the past 30 years. The results indicate that the changes in burden in high-middle and high SDI regions were particularly significant. In these regions, incidence, prevalence, mortality, and DALYs experienced substantial fluctuations. The changes in global GDSC mortality, prevalence, incidence, and DALYs were primarily driven by population growth, with contributing proportions of 64.65, 67.32, 49.01, and 56.69%, respectively. This phenomenon was observed across all SDI regions, especially in high-middle and high SDI regions.

Compared to population growth, epidemiological changes had a more significant effect on prevalence and incidence. In all regions, particularly in high-middle and high SDI regions, changes in epidemiological factors dominated the changes in prevalence and incidence. In high SDI regions, the incidence and prevalence of gastrointestinal cancers significantly increased due to the higher consumption of meat and high-fat foods in the diet, as well as a lack of sufficient physical activity. The results of this study indicate that, although the absolute health inequality of GDSC decreased between 1990 and 2021, relative health inequality showed an upward trend, which suggests that in low SDI countries, despite improvements in public health policies, cancer prevention and control have advanced, but the extent of disease burden reduction remains limited due to resource constraints and medical capabilities. This indicates that, despite some progress in global gastrointestinal cancer prevention and control, socioeconomic disparities remain a key factor contributing to health inequality. We further analyzed different types of GDSC and found that, although, globally, absolute health inequality in some cancer types has narrowed, relative health inequality has worsened in certain cancers (such as esophageal cancer, gastric cancer and liver cancer).

Frontier analysis reveals the gaps and potential for improvement in GDSC burden management across countries. Low-SDI countries can still achieve certain control effects through effective public health measures and rational resource allocation. In contrast, high-SDI countries, despite having abundant medical resources, still have room for improvement, particularly in early screening, treatment equity, and the management of high-risk populations. Future public health strategies should focus on improving early diagnosis in low-SDI regions as well as further optimizing prevention and control measures in high-SDI countries to reduce cancer burden inequality.

This study explored the risk factors contributing to various types of GDSC using the GBD database. Key contributors to esophageal cancer include smoking, alcohol consumption, and insufficient vegetable intake, which may reduce antioxidant levels and increase cancer risk. For stomach cancer, tobacco use and high sodium consumption, particularly in regions with high salt intake like Asia, are significant risk factors. Prolonged salt consumption can damage the stomach lining and promote *Helicobacter pylori* growth, increasing stomach cancer risk (14). Liver cancer is influenced by alcohol consumption, substance abuse, metabolic issues (e.g., high fasting blood sugar and BMI) (15), and lack of physical activity (16). Persistent hepatitis B or C infections and heavy drinking are strongly linked to liver cancer, while rising obesity rates contribute to its increasing prevalence (17). Colorectal cancer is associated with smoking, alcohol consumption, and poor dietary habits, including low intake of whole grains and dairy, excessive consumption of red and processed meats, and insufficient fiber and calcium. The global dietary shift over the past 30 years, especially increased red and processed meat consumption, has contributed to a rise in colorectal cancer (18). For pancreatic cancer, metabolic risk factors such as high fasting blood sugar and elevated BMI are important, while obesity and diabetes also exacerbate the malignancy. The activation of the Insulin/IGF-1 pathway promotes pancreatic cancer cell growth (19). Smoking remains a significant risk factor for this disease as well. For GBTC, the main risk factor is metabolic related (high BMI), which is associated with the global obesity epidemic. Obesity increases GBTC risk, likely linked to gallstones and chronic bile duct inflammation. Studies suggest that the relationship between obesity and GBTC involves mechanisms such as chronic inflammation and abnormal immune responses (20). Emerging risks such as obesity and high fasting glucose have become increasingly important in the development of GDSC, highlighting the need for targeted prevention and treatment strategies.

Over the past three decades, early screening technologies have continuously evolved, with gastroscopy, colonoscopy, and liver ultrasonography becoming routine screening tools. Molecular biomarkers and genetic testing have also provided new perspectives for early diagnosis (21). Personalized and non-invasive screening methods have been widely adopted among the older adults. Future research should focus on early diagnostic markers, personalized screening, and novel preventive strategies to enhance the survival rates and quality of life for older patients.

Over the past 30 years, significant progress has been made in the treatment of gastrointestinal cancers in older patients. The innovation and application of various treatment methods have significantly improved treatment outcomes and survival rates for patients. Advances in surgical techniques, particularly the widespread use of minimally invasive techniques such as laparoscopy and robot-assisted surgery, have greatly improved patient prognosis. For example, laparoscopic surgery for gastric cancer has reduced surgical trauma and recovery time, thus improving postoperative quality of life. Robotic surgery for colorectal cancer has enhanced the accuracy of tumor resection and reduced postoperative complications. Significant advancements have also been made in the field of radiotherapy for digestive system and related cancers (GDSC). For older patients who cannot tolerate surgery or those with systemic comorbidities, radical radiotherapy provides an important alternative treatment option. Preoperative or postoperative radiotherapy for esophageal cancer has become a standard treatment, significantly improving local control rates (22). Radiotherapy for gastric cancer, colorectal cancer, and liver cancer has also played a crucial role in conversion therapy, transforming inoperable into operable cases, with some patients achieving organ preservation and improved quality of life. Furthermore, advancements in radiotherapy technology, such as intensity-modulated radiotherapy (IMRT) and stereotactic body radiotherapy (SBRT), have increased treatment precision while reducing side effects. Immunotherapy have been demonstrated significant efficacy in gastrointestinal cancers in multiple clinical trials, including the KEYNOTE-177 (23), CheckMate-648 (24), and GEMSTONE-303 (25) trials. Finally, the progress in comprehensive treatment and the adoption of the multidisciplinary team (MDT) approach have enabled more effective combinations of different treatment modalities.

In this study, we predicted the epidemiological trends of GDSC over the next 30 years based on BAPC. It is important to note that these forecasts are based on current trends and assumptions. However, they do not account for potential future changes in healthcare systems and policies, or unexpected shifts in environmental or behavioral factors that could influence cancer incidence and mortality rates. Therefore, while these projections offer valuable insights, they should be interpreted with caution, and regular updates to models may be necessary as new data becomes available.

In the future, to reduce the burden of GDSC, targeted preventive measures should be focused on controlling major risk factors such as tobacco use, alcohol consumption, unhealthy diets, and metabolic conditions such as obesity and diabetes. As indicated in our study, for the older adults in the high-income Asia Pacific region, there is a continued need to strengthen prevention and screening efforts for colorectal cancer, liver cancer, pancreatic cancer and GBTC. In contrast, esophageal cancer and gastric cancer remain key targets for prevention and control in East Asia. In high-SDI regions, efforts should be concentrated on lifestyle interventions and improving access to early diagnosis and treatment, while low-SDI regions require strengthening healthcare infrastructure and increasing cancer awareness. Additionally, public health campaigns promoting healthier diets and physical activity can significantly reduce the incidence of major GDSC types.

## Data Availability

Publicly available datasets were analyzed in this study. This data can be found here: https://ghdx.healthdata.org/gbd-2021, GBD 2021.

## Footnotes

^1^ http://ghdx.healthdata.org/
