# Supplementary material for: Global, regional, and national epidemiology and risk factors of geriatric digestive system cancers from 1990 to 2021
Source: Front Public Health. 2025 Oct 21;13:1629101. doi: 10.3389/fpubh.2025.1629101 (PMC12583048; doi:10.3389/fpubh.2025.1629101)
Supplement: Supplementary file 1 [file Table_1.DOCX]

**TableS1 Incident, prevalent, DALYs and death cases for GDSC in 1990 and 2021 and percentage change of age-standardized rates by Global Burden of Disease (GBD) region**

|  |  | Death |  |  |  |  |  | DALYs |  |  |  |  |  | Incidence |  |  |  |  |  | Prevalence |  |  |  |  |  |
| --- | --- | --- | --- | --- | --- | --- | --- | --- | --- | --- | --- | --- | --- | --- | --- | --- | --- | --- | --- | --- | --- | --- | --- | --- | --- |
|  | Location | Number of cases,1990(95%UI) | Age-standardized  rate per 100 000  population, 1990(95%UI) | Number of cases,2021(95%UI) | Age-standardized  rate per 100 000  population, 2021(95%UI) | Estimated annual  percentage change,  1990–2021(95%UI) | AAPCs_(95%CI) | Number of cases,1990(95%UI) | Age-standardized  rate per 100 000  population, 1990(95%UI) | Number of cases,2021(95%UI) | Age-standardized  rate per 100 000  population, 2021(95%UI) | Estimated annual  percentage change,  1990–2021(95%CI) | AAPCs_(95%CI) | Number of cases,1990(95%UI) | Age-standardized  rate per 100 000  population, 1990(95%UI) | Number of cases,2021(95%UI) | Age-standardized  rate per 100 000  population, 2021(95%UI) | Estimated annual  percentage change,  1990–2021(95%CI) | AAPCs_(95%CI) | Number of cases,1990(95%UI) | Age-standardized  rate per 100 000  population, 1990(95%UI) | Number of cases,2021(95%UI) | Age-standardized  rate per 100 000  population, 2021(95%UI) | Estimated annual  percentage change,  1990–2021(95%CI) | AAPCs_(95%CI) |
| Digestive | System Cancer |  |  |  |  |  |  |  |  |  |  |  |  |  |  |  |  |  |  |  |  |  |  |  |  |
| 1 | Global | 1619349 (1471918, 1758646) | 356.79 (322.52, 387.52) | 2856742 (2491799, 3175587) | 270.14 (234.96, 300.30) | -0.96 (-1.01, -0.91) | -1.26 (-1.75, -0.76) | 32932702 (30031872, 35795486) | 6838.74 (6219.84, 7433.08) | 53939966 (47584531, 59978277) | 4980.95 (4386.30, 5538.14) | -1.10 (-1.15, -1.05) | -1.42 (-12.79, 9.95) | 1876969 (1718381, 2019022) | 405.48 (369.19, 436.45) | 3854209 (3376252, 4272039) | 360.89 (315.27, 400.05) | -0.42 (-0.46, -0.38) | -0.57 (-0.80, -0.33) | 4449183 (4128103, 4763134) | 939.55 (867.41, 1009.98) | 11282396 (10092703, 12349565) | 1043.24 (931.55, 1142.11) | 0.36 (0.30, 0.43) | 0.54 (0.12, 0.95) |
| 2 | Male | 907595 (813812, 1011088) | 458.28 (410.94, 509.39) | 1703950 (1464367, 1950097) | 362.16 (310.73, 413.37) | -0.80 (-0.86, -0.74) | -1.08 (-1.63, -0.52) | 19296771 (17261691, 21535065) | 8912.68 (7980.72, 9934.02) | 33436363 (28810127, 38408310) | 6730.44 (5794.72, 7717.10) | -0.96 (-1.01, -0.90) | -1.26 (-13.15, 10.62) | 1050847 (953009, 1155953) | 516.11 (467.55, 566.95) | 2327140 (2011731, 2654944) | 483.50 (417.30, 550.34) | -0.23 (-0.28, -0.17) | -0.30 (-0.61, 0.01) | 2390002 (2211708, 2586378) | 1136.23 (1047.69, 1234.90) | 6729163 (5955840, 7565773) | 1359.52 (1202.72, 1526.03) | 0.65 (0.57, 0.72) | 0.96 (0.46, 1.46) |
| 3 | Female | 711754 (621766, 783415) | 278.56 (242.14, 306.74) | 1152792 (961077, 1298051) | 195.53 (163.15, 220.11) | -1.25 (-1.29, -1.20) | -2.15 (-2.27, -2.03) | 13635930 (11967884, 15042085) | 5146.69 (4505.12, 5678.10) | 20503603 (17466644, 22994274) | 3488.21 (2973.13, 3911.28) | -1.38 (-1.43, -1.32) | -2.34 (-4.38, -0.31) | 826121 (728839, 899969) | 318.63 (279.77, 347.45) | 1527069 (1276941, 1718204) | 259.40 (217.03, 291.80) | -0.76 (-0.79, -0.72) | -0.76 (-2.05, 0.53) | 2059181 (1862730, 2231063) | 780.78 (703.73, 848.68) | 4553232 (3915279, 5062335) | 774.81 (666.41, 861.31) | -0.06 (-0.11, -0.01) | -0.06 (-0.54, 0.42) |
| 4 | High SDI | 538943 (498317, 565039) | 373.01 (343.83, 391.58) | 837039 (727152, 907008) | 280.29 (246.42, 302.43) | -1.01 (-1.05, -0.97) | -1.07 (-2.00, -0.14) | 10085543 (9464142, 10531820) | 6966.85 (6530.17, 7278.91) | 14246972 (12756308, 15300229) | 5042.04 (4554.19, 5398.12) | -1.15 (-1.20, -1.10) | -1.21 (-19.37, 16.96) | 765031 (709667, 800595) | 526.66 (487.75, 551.57) | 1344396 (1181447, 1450561) | 463.32 (411.28, 498.04) | -0.51 (-0.60, -0.42) | -0.44 (-1.63, 0.76) | 2472527 (2309357, 2618450) | 1697.75 (1584.93, 1799.88) | 4896018 (4410213, 5234038) | 1736.90 (1574.43, 1851.77) | 0.04 (-0.10, 0.18) | 0.07 (-1.49, 1.63) |
| 5 | High-middle SDI | 516683 (466050, 561699) | 436.38 (391.90, 474.62) | 837024 (709719, 962339) | 332.05 (281.20, 381.55) | -0.93 (-1.01, -0.85) | -1.25 (-2.06, -0.43) | 10680619 (9658044, 11624954) | 8509.98 (7678.39, 9263.22) | 15958503 (13607839, 18424794) | 6226.79 (5306.01, 7185.66) | -1.07 (-1.15, -0.99) | -1.45 (-16.89, 13.98) | 558697 (506303, 605064) | 461.02 (416.19, 499.56) | 1140981 (968269, 1315379) | 448.47 (380.22, 516.73) | -0.08 (-0.13, -0.02) | -0.10 (-0.57, 0.38) | 1131044 (1036069, 1226794) | 906.48 (827.28, 986.96) | 3356916 (2910154, 3832007) | 1305.75 (1131.50, 1489.90) | 1.33 (1.25, 1.41) | 2.09 (0.96, 3.22) |
| 6 | Middle SDI | 414609 (357305, 482086) | 373.26 (322.07, 433.39) | 842083 (708176, 986105) | 269.71 (226.26, 315.46) | -1.12 (-1.19, -1.04) | -1.47 (-2.24, -0.71) | 8918704 (7666873, 10374394) | 7375.71 (6349.95, 8573.38) | 16641875 (14040781, 19508434) | 5067.04 (4269.36, 5934.86) | -1.27 (-1.33, -1.20) | -1.70 (-15.98, 12.57) | 411950 (354761, 478523) | 359.18 (309.73, 416.84) | 1033274 (864796, 1216974) | 322.73 (269.61, 379.69) | -0.36 (-0.41, -0.32) | -0.65 (-0.86, -0.45) | 642630 (556192, 741132) | 531.91 (460.68, 613.97) | 2469528 (2087782, 2884302) | 742.75 (627.87, 866.63) | 1.20 (1.12, 1.28) | 1.45 (-0.44, 3.34) |
| 7 | Low-middle SDI | 99613 (85533, 121685) | 154.59 (132.41, 188.78) | 247475 (217588, 281568) | 154.04 (134.97, 175.29) | 0.02 (-0.01, 0.05) | 0.02 (-0.08, 0.13) | 2159804 (1856902, 2637788) | 3076.87 (2642.32, 3758.09) | 5142313 (4530811, 5853524) | 3003.30 (2641.93, 3418.50) | -0.07 (-0.10, -0.04) | -0.11 (-2.28, 2.06) | 94558 (81246, 115203) | 142.43 (122.12, 173.54) | 246204 (216749, 279583) | 149.49 (131.19, 169.78) | 0.18 (0.15, 0.21) | 0.28 (0.19, 0.38) | 137575 (119153, 164113) | 196.89 (170.30, 235.17) | 422104 (376715, 473969) | 245.61 (218.93, 275.74) | 0.74 (0.71, 0.78) | 1.02 (0.76, 1.27) |
| 8 | Low SDI | 47765 (37720, 57713) | 202.04 (159.56, 245.06) | 90546 (74602, 106087) | 176.64 (145.38, 206.85) | -0.48 (-0.54, -0.42) | -0.73 (-0.91, -0.56) | 1053929 (831237, 1270856) | 4053.56 (3199.28, 4898.80) | 1901852 (1565921, 2234593) | 3393.38 (2793.90, 3982.60) | -0.65 (-0.70, -0.60) | -1.02 (-4.03, 1.98) | 44833 (35377, 54046) | 183.56 (144.88, 222.03) | 85998 (70932, 100508) | 162.46 (133.81, 189.78) | -0.45 (-0.51, -0.39) | -0.69 (-0.83, -0.55) | 61130 (48786, 72477) | 235.81 (188.50, 280.32) | 127514 (107723, 147048) | 227.15 (191.93, 261.62) | -0.18 (-0.25, -0.11) | -0.24 (-0.37, -0.11) |
| 9 | Andean Latin America | 8008 (6700, 9491) | 354.98 (296.81, 420.96) | 20401 (15759, 25919) | 289.39 (223.64, 367.41) | -0.78 (-0.89, -0.67) | -1.33 (-1.74, -0.91) | 154563 (129316, 183109) | 6603.88 (5524.87, 7824.93) | 376499 (289911, 480622) | 5265.76 (4056.44, 6718.37) | -0.87 (-0.98, -0.76) | -1.41 (-8.12, 5.31) | 7416 (6207, 8796) | 324.64 (271.53, 385.13) | 20996 (16181, 26729) | 296.19 (228.34, 376.82) | -0.41 (-0.52, -0.30) | -0.58 (-0.98, -0.19) | 10796 (9062, 12799) | 462.88 (388.48, 548.91) | 46644 (35879, 59608) | 653.43 (502.69, 834.70) | 1.18 (1.07, 1.28) | 2.34 (1.68, 3.01) |
| 10 | Australasia | 9283 (8239, 10294) | 306.93 (271.03, 341.12) | 17431 (14574, 20125) | 232.24 (195.02, 267.67) | -1.01 (-1.09, -0.94) | -1.64 (-1.85, -1.43) | 177757 (159382, 196422) | 5751.08 (5143.17, 6362.10) | 299889 (255008, 344623) | 4165.66 (3553.92, 4781.42) | -1.20 (-1.27, -1.13) | -1.97 (-5.72, 1.79) | 14381 (12740, 15976) | 467.83 (413.12, 520.57) | 31137 (25918, 36314) | 423.83 (353.83, 493.70) | -0.42 (-0.55, -0.30) | -0.29 (-1.88, 1.29) | 53883 (47836, 60219) | 1731.38 (1534.46, 1938.54) | 129377 (109038, 150043) | 1791.77 (1511.52, 2077.04) | 0.05 (-0.20, 0.30) | 0.10 (-3.01, 3.21) |
| 11 | Caribbean | 7543 (6780, 8362) | 245.42 (219.98, 272.33) | 14209 (12073, 16435) | 210.17 (178.72, 243.05) | -0.36 (-0.42, -0.31) | -0.53 (-0.87, -0.19) | 143498 (128981, 159531) | 4509.99 (4047.99, 5016.18) | 266173 (226442, 308483) | 3961.55 (3371.37, 4591.13) | -0.29 (-0.35, -0.23) | -0.43 (-5.31, 4.45) | 8951 (8041, 9932) | 285.06 (255.52, 316.57) | 21165 (17857, 24555) | 314.63 (265.56, 365.00) | 0.46 (0.42, 0.50) | 0.46 (-2.74, 3.66) | 21486 (19266, 23867) | 667.39 (597.79, 742.15) | 71466 (59983, 82890) | 1065.89 (894.54, 1236.26) | 1.74 (1.64, 1.84) | 2.75 (1.87, 3.64) |
| 12 | Central Asia | 18885 (17333, 20553) | 345.83 (316.58, 377.03) | 19874 (17493, 22442) | 219.39 (193.01, 247.41) | -1.24 (-1.37, -1.12) | -1.77 (-2.93, -0.61) | 408843 (377308, 443569) | 7175.30 (6609.70, 7795.00) | 430217 (379049, 486614) | 4400.33 (3878.36, 4969.17) | -1.40 (-1.50, -1.30) | -1.95 (-21.01, 17.11) | 18401 (16912, 19995) | 332.50 (304.92, 361.84) | 20422 (17989, 23018) | 219.76 (193.54, 247.38) | -1.08 (-1.21, -0.94) | -1.58 (-2.68, -0.49) | 28798 (26510, 31303) | 507.06 (466.23, 551.95) | 37288 (33109, 41704) | 382.24 (339.76, 426.80) | -0.58 (-0.78, -0.38) | -1.03 (-2.58, 0.52) |
| 13 | Central Europe | 68199 (64215, 71697) | 368.36 (345.17, 388.05) | 96458 (87305, 104744) | 314.74 (284.91, 341.82) | -0.62 (-0.67, -0.56) | -0.63 (-1.60, 0.33) | 1358751 (1284277, 1427167) | 6999.25 (6598.85, 7359.45) | 1816486 (1654999, 1971145) | 6002.46 (5469.69, 6513.93) | -0.59 (-0.64, -0.54) | -0.61 (-16.61, 15.40) | 71273 (67050, 75033) | 376.77 (353.16, 397.23) | 119908 (108645, 130723) | 393.03 (356.14, 428.52) | 0.07 (0.00, 0.14) | 0.16 (-0.22, 0.55) | 147819 (138067, 158332) | 758.75 (706.59, 815.42) | 354466 (322120, 386744) | 1170.28 (1063.55, 1276.85) | 1.49 (1.36, 1.63) | 2.19 (1.55, 2.83) |
| 14 | Central Latin America | 24319 (22842, 25607) | 274.73 (256.73, 289.72) | 59537 (52337, 66754) | 199.33 (175.15, 223.41) | -1.21 (-1.29, -1.13) | -1.94 (-2.09, -1.78) | 468150 (442368, 492203) | 4994.42 (4705.71, 5255.32) | 1140417 (1008010, 1280125) | 3725.64 (3291.90, 4180.70) | -1.14 (-1.23, -1.06) | -1.82 (-4.64, 1.00) | 23633 (22203, 24869) | 261.04 (244.23, 275.03) | 70401 (61889, 79129) | 233.11 (204.82, 261.91) | -0.54 (-0.63, -0.46) | -0.79 (-0.93, -0.65) | 35920 (33699, 38022) | 382.20 (357.67, 405.11) | 172131 (151169, 193893) | 560.88 (492.39, 631.74) | 1.18 (1.10, 1.25) | 2.13 (1.85, 2.41) |
| 15 | Central Sub-Saharan Africa | 5275 (3608, 7620) | 238.77 (162.82, 348.35) | 10084 (6805, 14993) | 199.19 (133.55, 301.29) | -0.68 (-0.77, -0.59) | -1.04 (-1.21, -0.87) | 119704 (81852, 172831) | 4750.14 (3245.59, 6887.96) | 222326 (150203, 328585) | 3877.66 (2614.00, 5781.43) | -0.75 (-0.84, -0.67) | -1.15 (-4.80, 2.50) | 4971 (3408, 7160) | 215.30 (147.18, 312.42) | 9621 (6543, 14115) | 182.22 (123.16, 271.22) | -0.64 (-0.74, -0.54) | -0.96 (-1.12, -0.81) | 6692 (4751, 9320) | 267.53 (189.99, 373.53) | 14458 (10216, 20387) | 252.22 (178.23, 358.50) | -0.25 (-0.38, -0.12) | -0.36 (-0.51, -0.21) |
| 16 | East Asia | 542950 (453736, 641766) | 579.86 (484.37, 686.00) | 1043919 (823414, 1278012) | 393.98 (310.53, 481.13) | -1.31 (-1.47, -1.14) | -1.71 (-3.32, -0.09) | 11748612 (9789052, 13889681) | 11311.54 (9432.73, 13379.54) | 20296689 (16000814, 24985101) | 7319.18 (5769.34, 8996.19) | -1.46 (-1.61, -1.32) | -1.93 (-31.16, 27.31) | 547623 (457724, 646429) | 561.17 (468.99, 662.95) | 1403921 (1107983, 1718214) | 515.88 (406.80, 630.19) | -0.25 (-0.36, -0.14) | -0.30 (-1.63, 1.02) | 864882 (726151, 1014929) | 829.27 (696.03, 975.11) | 3717507 (2977440, 4513436) | 1318.00 (1055.51, 1598.27) | 1.69 (1.58, 1.79) | 1.86 (-11.48, 15.21) |
| 17 | Eastern Europe | 125151 (118990, 130812) | 350.39 (332.05, 366.72) | 129871 (117739, 141356) | 274.46 (248.81, 298.66) | -0.93 (-1.00, -0.85) | -1.53 (-1.89, -1.17) | 2666167 (2544888, 2784396) | 7179.97 (6841.56, 7503.16) | 2595355 (2362962, 2827858) | 5395.94 (4913.63, 5877.41) | -1.11 (-1.20, -1.03) | -1.86 (-10.10, 6.39) | 139982 (132932, 146724) | 386.13 (365.80, 405.10) | 169634 (154271, 184466) | 356.62 (324.30, 387.73) | -0.36 (-0.48, -0.24) | -0.53 (-1.40, 0.34) | 298776 (280869, 318849) | 809.47 (758.86, 866.51) | 478770 (438515, 520064) | 996.71 (913.01, 1082.15) | 0.67 (0.49, 0.85) | 0.93 (-1.39, 3.26) |
| 18 | Eastern Sub-Saharan Africa | 20545 (16217, 24624) | 264.52 (209.06, 317.72) | 38366 (31490, 46603) | 233.46 (191.35, 283.17) | -0.57 (-0.62, -0.51) | -0.51 (-2.07, 1.04) | 453369 (356964, 542933) | 5306.29 (4185.20, 6361.06) | 806207 (660804, 981989) | 4455.69 (3652.92, 5416.70) | -0.75 (-0.81, -0.69) | -0.71 (-32.96, 31.54) | 19274 (15190, 23071) | 240.12 (189.45, 287.89) | 36377 (29876, 44109) | 214.13 (175.64, 259.25) | -0.55 (-0.60, -0.49) | -0.90 (-1.05, -0.75) | 27177 (21645, 32025) | 318.71 (254.49, 376.15) | 56548 (47370, 67479) | 312.52 (261.95, 372.13) | -0.21 (-0.27, -0.14) | -0.22 (-0.34, -0.10) |
| 19 | High-income Asia Pacific | 136384 (124368, 145532) | 564.61 (510.97, 603.96) | 267664 (220375, 298899) | 368.79 (311.83, 408.66) | -1.52 (-1.59, -1.46) | -1.60 (-4.23, 1.04) | 2665485 (2455009, 2839067) | 10595.02 (9717.81, 11299.51) | 4155413 (3543054, 4599614) | 6458.90 (5617.77, 7114.88) | -1.77 (-1.85, -1.69) | -1.83 (-54.83, 51.17) | 206880 (189475, 220524) | 838.07 (763.12, 895.22) | 445392 (370886, 500254) | 656.57 (558.67, 732.23) | -0.91 (-0.99, -0.84) | -0.79 (-7.06, 5.48) | 620738 (572048, 666123) | 2458.76 (2257.91, 2645.69) | 1552273 (1324736, 1735619) | 2454.85 (2124.07, 2727.56) | -0.05 (-0.14, 0.04) | -0.31 (-2.14, 1.52) |
| 20 | High-income North America | 126935 (115657, 133671) | 268.09 (244.14, 282.40) | 190594 (168999, 203529) | 211.26 (187.99, 225.29) | -0.87 (-0.91, -0.83) | -1.49 (-1.64, -1.35) | 2365601 (2202141, 2478889) | 5052.24 (4706.01, 5293.08) | 3524885 (3215941, 3733701) | 3967.20 (3627.31, 4198.47) | -0.89 (-0.93, -0.84) | -1.48 (-4.39, 1.43) | 210413 (192535, 221659) | 444.40 (406.71, 468.16) | 331012 (295639, 353383) | 370.21 (331.37, 394.87) | -0.76 (-0.87, -0.66) | -0.79 (-1.59, 0.01) | 832759 (770023, 889790) | 1762.45 (1630.90, 1883.23) | 1301840 (1185874, 1385149) | 1465.82 (1336.31, 1558.96) | -0.75 (-0.90, -0.60) | -0.81 (-3.45, 1.83) |
| 21 | North Africa and Middle East | 37511 (29255, 46040) | 215.82 (168.24, 265.83) | 93419 (74177, 109466) | 199.03 (157.90, 233.04) | -0.09 (-0.21, 0.03) | -0.18 (-0.63, 0.26) | 790477 (615919, 966848) | 4131.70 (3221.23, 5064.54) | 1880998 (1494160, 2209865) | 3701.39 (2939.57, 4343.20) | -0.23 (-0.33, -0.13) | -0.37 (-7.78, 7.05) | 36463 (28493, 44487) | 202.66 (158.29, 248.14) | 104241 (83228, 122239) | 214.69 (171.08, 251.62) | 0.37 (0.24, 0.50) | 0.45 (-0.02, 0.92) | 64025 (52017, 75778) | 336.89 (273.82, 399.98) | 268309 (222283, 313043) | 527.75 (437.03, 615.50) | 1.66 (1.53, 1.78) | 2.25 (1.20, 3.30) |
| 22 | Oceania | 523 (378, 723) | 192.37 (141.45, 261.77) | 1093 (830, 1477) | 159.46 (121.72, 213.07) | -0.62 (-0.66, -0.58) | -0.97 (-1.02, -0.91) | 11625 (8311, 16191) | 3679.79 (2669.20, 5063.93) | 23429 (17690, 31861) | 3022.45 (2294.11, 4076.93) | -0.65 (-0.70, -0.61) | -1.00 (-2.10, 0.10) | 505 (365, 697) | 175.87 (128.98, 239.29) | 1077 (823, 1442) | 150.05 (115.13, 199.16) | -0.54 (-0.58, -0.50) | -0.84 (-0.88, -0.79) | 732 (550, 969) | 232.39 (176.39, 304.89) | 1683 (1334, 2155) | 215.74 (171.75, 274.73) | -0.27 (-0.33, -0.21) | -0.39 (-0.50, -0.28) |
| 23 | South Asia | 70989 (59429, 89025) | 117.36 (97.71, 147.71) | 199317 (169373, 233072) | 118.79 (100.70, 139.03) | -0.01 (-0.08, 0.07) | 0.04 (-0.09, 0.18) | 1583649 (1330834, 1979404) | 2395.80 (2006.15, 3002.63) | 4177127 (3559923, 4883055) | 2335.59 (1987.72, 2731.58) | -0.16 (-0.24, -0.08) | -0.18 (-2.41, 2.04) | 67490 (56627, 84403) | 108.22 (90.33, 135.82) | 195712 (166716, 228349) | 113.78 (96.68, 132.86) | 0.10 (0.02, 0.18) | 0.27 (0.15, 0.38) | 96530 (81928, 118207) | 146.24 (123.71, 179.71) | 312081 (270947, 360290) | 173.56 (150.48, 200.41) | 0.45 (0.35, 0.55) | 1.01 (0.87, 1.15) |
| 24 | Southeast Asia | 53193 (43675, 63049) | 197.86 (162.42, 234.92) | 144266 (118996, 175064) | 199.71 (164.22, 242.12) | -0.07 (-0.11, -0.03) | 0.03 (-0.61, 0.67) | 1142897 (936824, 1353512) | 3919.92 (3215.74, 4644.85) | 3006316 (2485672, 3655731) | 3837.25 (3168.90, 4660.42) | -0.17 (-0.21, -0.13) | -0.09 (-11.83, 11.65) | 51590 (42374, 61076) | 186.21 (152.90, 220.77) | 159524 (131739, 192817) | 213.09 (175.47, 257.44) | 0.34 (0.30, 0.37) | 0.49 (-0.18, 1.16) | 85557 (71503, 100029) | 294.09 (245.77, 344.56) | 368023 (309135, 433577) | 465.16 (390.60, 547.63) | 1.46 (1.43, 1.49) | 2.72 (2.59, 2.86) |
| 25 | Southern Latin America | 23605 (21016, 26293) | 419.35 (371.82, 468.01) | 35493 (30518, 40385) | 309.49 (266.31, 352.10) | -0.82 (-0.92, -0.71) | -1.00 (-1.81, -0.18) | 452066 (404185, 502728) | 7724.30 (6890.90, 8598.51) | 645018 (560173, 732245) | 5699.18 (4951.87, 6470.02) | -0.84 (-0.94, -0.74) | -1.06 (-12.34, 10.22) | 23651 (21054, 26388) | 412.99 (366.44, 461.42) | 40497 (34745, 46270) | 354.90 (304.65, 405.50) | -0.34 (-0.45, -0.23) | -0.33 (-1.25, 0.59) | 42827 (37894, 48061) | 730.00 (644.79, 820.67) | 98563 (84685, 113152) | 870.62 (747.93, 999.83) | 0.79 (0.66, 0.91) | 1.17 (-0.41, 2.74) |
| 26 | Southern Sub-Saharan Africa | 6292 (4986, 8037) | 217.20 (171.91, 277.38) | 16107 (13824, 18420) | 261.29 (223.50, 298.78) | 0.39 (0.00, 0.78) | 0.87 (-0.10, 1.84) | 125340 (99372, 160195) | 4030.69 (3193.64, 5150.33) | 330652 (284228, 378446) | 4917.64 (4219.97, 5626.32) | 0.45 (0.04, 0.85) | 0.94 (-18.46, 20.34) | 5872 (4662, 7478) | 197.82 (156.83, 251.94) | 15678 (13458, 17916) | 246.42 (210.92, 281.52) | 0.52 (0.15, 0.88) | 1.00 (0.20, 1.80) | 8671 (7080, 10958) | 280.28 (228.48, 354.24) | 24944 (21708, 28296) | 369.47 (321.24, 418.67) | 0.80 (0.56, 1.04) | 1.31 (-0.02, 2.64) |
| 27 | Tropical Latin America | 27392 (25356, 29074) | 280.30 (256.73, 298.74) | 68929 (61567, 74518) | 219.69 (195.63, 237.75) | -0.75 (-0.79, -0.70) | -1.22 (-1.42, -1.02) | 552541 (515899, 584497) | 5238.70 (4860.70, 5554.93) | 1341248 (1219106, 1441074) | 4177.71 (3788.50, 4492.07) | -0.72 (-0.77, -0.68) | -1.21 (-4.25, 1.83) | 26486 (24544, 28111) | 263.35 (241.86, 280.44) | 73860 (66215, 79871) | 233.27 (208.52, 252.49) | -0.38 (-0.42, -0.33) | -0.62 (-0.78, -0.45) | 40965 (38106, 43670) | 387.18 (358.23, 413.81) | 162889 (147123, 176837) | 506.55 (456.66, 550.22) | 0.89 (0.84, 0.94) | 1.55 (1.21, 1.90) |
| 28 | Western Europe | 290184 (267318, 306968) | 372.11 (341.97, 394.03) | 356038 (307741, 388258) | 262.48 (230.52, 284.62) | -1.12 (-1.18, -1.07) | -1.73 (-1.91, -1.55) | 5203091 (4858598, 5474680) | 6742.49 (6295.13, 7095.52) | 5910704 (5250286, 6391071) | 4749.62 (4273.72, 5111.50) | -1.13 (-1.17, -1.08) | -1.73 (-5.25, 1.79) | 376758 (347650, 398741) | 482.25 (444.77, 510.58) | 552055 (482891, 601184) | 427.21 (378.83, 462.98) | -0.37 (-0.44, -0.30) | -0.27 (-2.15, 1.61) | 1141062 (1057730, 1221092) | 1467.74 (1361.43, 1571.48) | 2069320 (1856005, 2237997) | 1675.20 (1514.22, 1805.94) | 0.52 (0.35, 0.69) | 0.72 (-0.70, 2.14) |
| 29 | Western Sub-Saharan Africa | 16182 (12456, 21254) | 171.74 (132.54, 225.48) | 33671 (27186, 39868) | 175.07 (142.30, 206.51) | 0.16 (0.11, 0.21) | 0.14 (0.04, 0.25) | 340513 (261423, 447365) | 3352.72 (2579.30, 4403.07) | 693916 (555752, 825830) | 3309.09 (2665.42, 3924.93) | 0.04 (-0.01, 0.09) | -0.07 (-1.99, 1.85) | 14956 (11510, 19593) | 154.74 (119.32, 202.66) | 31579 (25449, 37386) | 159.49 (129.33, 188.11) | 0.19 (0.14, 0.24) | 0.21 (0.11, 0.31) | 19088 (15129, 24276) | 189.22 (150.19, 240.68) | 43814 (35477, 51776) | 209.46 (170.72, 246.49) | 0.45 (0.38, 0.52) | 0.56 (0.03, 1.09) |
| Esophageal cancer |  |  |  |  |  |  |  |  |  |  |  |  |  |  |  |  |  |  |  |  |  |  |  |  |  |
| 1 | Global | 238854 (209539, 263324) | 50.43 (44.21, 55.53) | 410902 (356634, 466427) | 38.37 (33.28, 43.55) | -1.06 (-1.19, -0.92) | -1.35 (-1.49, -1.21) | 5123634 (4497405, 5665726) | 1036.26 (909.30, 1144.96) | 8059156 (7029414, 9163785) | 738.20 (643.58, 839.21) | -1.25 (-1.37, -1.13) | -1.64 (-4.22, 0.94) | 226411 (198851, 249655) | 47.16 (41.39, 51.94) | 421336 (365896, 478329) | 39.08 (33.91, 44.36) | -0.75 (-0.86, -0.63) | -0.95 (-1.07, -0.84) | 299849 (263952, 329805) | 60.65 (53.37, 66.65) | 655313 (573097, 740831) | 59.97 (52.42, 67.79) | -0.09 (-0.22, 0.04) | -0.06 (-0.20, 0.07) |
| 2 | Male | 156838 (139147, 176224) | 75.01 (66.66, 83.89) | 298565 (254521, 347324) | 62.04 (52.94, 72.00) | -0.74 (-0.87, -0.61) | -0.93 (-1.12, -0.73) | 3464005 (3066391, 3911913) | 1544.74 (1369.39, 1739.48) | 5989518 (5099073, 6986011) | 1188.59 (1012.55, 1384.39) | -0.95 (-1.07, -0.84) | -1.24 (-4.38, 1.90) | 149360 (132706, 168129) | 69.88 (62.19, 78.34) | 306157 (260714, 356663) | 62.60 (53.35, 72.76) | -0.45 (-0.57, -0.33) | -0.56 (-0.70, -0.42) | 198761 (177593, 223155) | 88.56 (79.22, 99.15) | 468861 (403244, 542993) | 92.96 (79.99, 107.48) | 0.16 (0.02, 0.29) | 0.28 (0.11, 0.45) |
| 3 | Female | 82016 (57410, 95541) | 31.28 (22.03, 36.38) | 112338 (82884, 133651) | 19.08 (14.09, 22.70) | -1.89 (-2.04, -1.73) | -2.30 (-2.45, -2.15) | 1659629 (1134681, 1946553) | 617.42 (423.68, 723.37) | 2069638 (1545343, 2450516) | 352.25 (263.19, 416.92) | -2.11 (-2.26, -1.95) | -2.56 (-5.96, 0.83) | 77051 (53996, 89880) | 29.13 (20.52, 33.93) | 115179 (83759, 137244) | 19.58 (14.24, 23.32) | -1.55 (-1.69, -1.41) | -1.91 (-2.04, -1.78) | 101088 (70663, 117650) | 37.58 (26.37, 43.69) | 186452 (133418, 224180) | 31.70 (22.70, 38.11) | -0.75 (-0.89, -0.61) | -0.88 (-1.03, -0.74) |
| 4 | High SDI | 41107 (38688, 42845) | 28.44 (26.72, 29.66) | 72577 (65283, 77228) | 25.17 (22.83, 26.72) | -0.53 (-0.63, -0.43) | -0.59 (-0.64, -0.55) | 820138 (780368, 852702) | 569.13 (541.28, 591.90) | 1328693 (1220730, 1406530) | 483.41 (446.67, 510.98) | -0.66 (-0.76, -0.56) | -0.78 (-1.63, 0.07) | 42489 (39979, 44223) | 29.35 (27.60, 30.57) | 83972 (75807, 89144) | 29.58 (26.91, 31.33) | -0.06 (-0.21, 0.08) | -0.01 (-0.05, 0.02) | 67681 (63989, 70605) | 46.81 (44.25, 48.84) | 169429 (154335, 179995) | 61.31 (56.15, 65.01) | 0.92 (0.63, 1.21) | 1.29 (1.21, 1.38) |
| 5 | High-middle SDI | 76839 (66259, 86492) | 63.13 (54.35, 71.01) | 126638 (103190, 154105) | 49.91 (40.65, 60.71) | -0.93 (-1.11, -0.75) | -1.17 (-1.39, -0.95) | 1645949 (1420990, 1855744) | 1289.91 (1112.30, 1453.72) | 2469810 (2013621, 3023581) | 959.86 (782.34, 1174.83) | -1.10 (-1.26, -0.94) | -1.45 (-5.30, 2.41) | 71618 (61801, 80701) | 57.91 (49.90, 65.22) | 130867 (106061, 160396) | 51.27 (41.54, 62.80) | -0.52 (-0.67, -0.37) | -0.66 (-0.84, -0.47) | 90468 (77954, 101924) | 70.71 (60.86, 79.65) | 199293 (161108, 244887) | 77.20 (62.39, 94.88) | 0.25 (0.12, 0.38) | 0.43 (0.20, 0.66) |
| 6 | Middle SDI | 95333 (78011, 110607) | 82.85 (67.98, 95.74) | 159123 (131195, 191057) | 50.49 (41.59, 60.49) | -1.79 (-1.94, -1.63) | -2.38 (-2.65, -2.10) | 2093222 (1707875, 2438566) | 1695.58 (1386.12, 1969.58) | 3145295 (2596487, 3782554) | 952.78 (786.14, 1144.06) | -2.02 (-2.15, -1.88) | -2.73 (-7.85, 2.40) | 88893 (72513, 103348) | 75.45 (61.70, 87.40) | 158455 (130554, 191410) | 49.39 (40.65, 59.54) | -1.54 (-1.67, -1.40) | -2.05 (-2.30, -1.81) | 113238 (91471, 131902) | 91.23 (73.85, 106.01) | 227268 (186081, 275725) | 68.34 (55.91, 82.79) | -1.06 (-1.18, -0.95) | -1.42 (-1.72, -1.12) |
| 7 | Low-middle SDI | 15757 (13923, 18310) | 24.11 (21.20, 28.12) | 34541 (30781, 40299) | 21.27 (18.89, 24.88) | -0.48 (-0.54, -0.42) | -0.40 (-0.52, -0.28) | 344653 (305729, 399229) | 486.77 (430.42, 565.35) | 726007 (648729, 843470) | 421.06 (375.58, 490.09) | -0.56 (-0.62, -0.50) | -0.49 (-3.09, 2.10) | 14399 (12722, 16691) | 21.47 (18.88, 24.96) | 31592 (28174, 36705) | 19.07 (16.95, 22.21) | -0.46 (-0.52, -0.41) | -0.38 (-0.49, -0.27) | 17551 (15551, 20288) | 24.71 (21.83, 28.62) | 39098 (34919, 45327) | 22.61 (20.16, 26.27) | -0.36 (-0.42, -0.31) | -0.28 (-0.41, -0.15) |
| 8 | Low SDI | 9713 (8064, 11036) | 40.20 (33.30, 45.83) | 17850 (15051, 20802) | 33.86 (28.48, 39.56) | -0.67 (-0.74, -0.61) | -1.07 (-1.09, -1.04) | 217513 (180379, 246962) | 823.98 (683.19, 937.04) | 385860 (324991, 449795) | 674.22 (567.53, 786.44) | -0.79 (-0.85, -0.72) | -1.28 (-1.79, -0.76) | 8915 (7401, 10130) | 35.84 (29.70, 40.84) | 16285 (13738, 18945) | 30.05 (25.29, 35.04) | -0.70 (-0.76, -0.63) | -1.10 (-1.13, -1.08) | 10791 (8942, 12256) | 40.73 (33.73, 46.33) | 19995 (16837, 23296) | 34.79 (29.27, 40.58) | -0.62 (-0.68, -0.56) | -0.98 (-1.00, -0.96) |
| 9 | Andean Latin America | 317 (264, 379) | 14.09 (11.72, 16.86) | 706 (557, 896) | 10.04 (7.93, 12.73) | -1.13 (-1.24, -1.02) | -1.81 (-1.83, -1.80) | 6056 (5037, 7260) | 259.60 (215.95, 311.08) | 12712 (9979, 16190) | 178.23 (139.99, 226.91) | -1.27 (-1.39, -1.15) | -2.01 (-2.23, -1.79) | 278 (231, 332) | 12.23 (10.16, 14.58) | 626 (492, 795) | 8.86 (6.97, 11.26) | -1.08 (-1.20, -0.96) | -1.70 (-1.71, -1.69) | 318 (264, 379) | 13.61 (11.32, 16.23) | 728 (568, 931) | 10.20 (7.97, 13.05) | -0.98 (-1.11, -0.85) | -1.48 (-1.49, -1.47) |
| 10 | Australasia | 857 (755, 962) | 28.05 (24.64, 31.52) | 1791 (1488, 2087) | 24.11 (20.09, 28.08) | -0.65 (-0.73, -0.57) | -0.46 (-0.83, -0.10) | 16727 (14803, 18738) | 539.05 (476.33, 604.09) | 31089 (26203, 36135) | 435.92 (368.40, 506.51) | -0.82 (-0.89, -0.75) | -0.70 (-6.98, 5.59) | 861 (758, 966) | 27.98 (24.57, 31.41) | 1867 (1553, 2172) | 25.49 (21.27, 29.64) | -0.44 (-0.53, -0.35) | -0.22 (-0.49, 0.05) | 1304 (1147, 1473) | 41.87 (36.77, 47.33) | 3116 (2607, 3652) | 43.64 (36.61, 51.13) | 0.13 (-0.02, 0.28) | 0.26 (0.16, 0.36) |
| 11 | Caribbean | 780 (707, 861) | 25.18 (22.77, 27.78) | 1380 (1178, 1595) | 20.48 (17.49, 23.68) | -0.44 (-0.58, -0.30) | -0.68 (-0.74, -0.62) | 15267 (13821, 16868) | 477.79 (432.25, 527.89) | 27424 (23369, 31751) | 409.09 (348.68, 473.62) | -0.26 (-0.40, -0.11) | -0.41 (-1.49, 0.67) | 704 (637, 776) | 22.43 (20.28, 24.71) | 1275 (1089, 1474) | 18.98 (16.21, 21.93) | -0.30 (-0.44, -0.17) | -0.49 (-0.54, -0.43) | 844 (765, 929) | 26.32 (23.84, 28.96) | 1636 (1394, 1895) | 24.43 (20.81, 28.29) | 0.03 (-0.11, 0.16) | -0.00 (-0.08, 0.07) |
| 12 | Central Asia | 4376 (4043, 4697) | 80.76 (74.48, 86.74) | 2645 (2343, 2969) | 29.32 (25.97, 32.87) | -3.31 (-3.47, -3.14) | -4.83 (-5.08, -4.57) | 92956 (86331, 99535) | 1643.59 (1525.09, 1760.25) | 57040 (50509, 64098) | 585.28 (518.92, 656.60) | -3.38 (-3.53, -3.23) | -4.84 (-10.99, 1.32) | 3958 (3664, 4244) | 72.17 (66.70, 77.45) | 2421 (2145, 2719) | 26.26 (23.27, 29.45) | -3.30 (-3.46, -3.13) | -4.77 (-5.03, -4.51) | 4825 (4488, 5164) | 85.36 (79.32, 91.39) | 3014 (2673, 3383) | 30.85 (27.38, 34.57) | -3.29 (-3.46, -3.11) | -4.79 (-5.11, -4.48) |
| 13 | Central Europe | 2889 (2726, 3045) | 15.26 (14.34, 16.09) | 4541 (4113, 4933) | 15.05 (13.63, 16.35) | -0.26 (-0.35, -0.17) | -0.02 (-0.22, 0.17) | 60826 (57506, 64076) | 306.53 (289.27, 323.02) | 94004 (85464, 102124) | 316.54 (287.68, 343.90) | -0.10 (-0.19, -0.01) | 0.15 (-2.55, 2.85) | 2634 (2486, 2776) | 13.69 (12.88, 14.43) | 4253 (3853, 4623) | 14.15 (12.81, 15.38) | -0.10 (-0.19, -0.01) | 0.15 (-0.01, 0.32) | 3224 (3046, 3396) | 16.21 (15.29, 17.08) | 5693 (5167, 6201) | 19.12 (17.35, 20.82) | 0.39 (0.31, 0.47) | 0.68 (0.55, 0.81) |
| 14 | Central Latin America | 1603 (1500, 1699) | 18.17 (16.92, 19.29) | 3083 (2694, 3492) | 10.35 (9.04, 11.71) | -1.96 (-2.03, -1.88) | -3.25 (-3.27, -3.24) | 30770 (28952, 32586) | 328.99 (308.63, 348.64) | 57960 (50775, 65880) | 189.77 (166.27, 215.52) | -1.94 (-2.02, -1.85) | -3.27 (-3.56, -2.98) | 1418 (1328, 1504) | 15.76 (14.70, 16.73) | 2771 (2424, 3144) | 9.23 (8.08, 10.47) | -1.88 (-1.95, -1.80) | -3.13 (-3.14, -3.11) | 1649 (1551, 1748) | 17.57 (16.48, 18.63) | 3349 (2934, 3806) | 10.96 (9.61, 12.45) | -1.67 (-1.74, -1.59) | -2.85 (-2.86, -2.83) |
| 15 | Central Sub-Saharan Africa | 1465 (1064, 1927) | 64.19 (46.66, 84.99) | 2699 (1916, 3642) | 51.84 (36.59, 71.28) | -0.81 (-0.90, -0.72) | -1.25 (-1.31, -1.19) | 33734 (24467, 44409) | 1307.43 (949.71, 1723.54) | 60614 (43000, 81348) | 1033.14 (732.48, 1398.24) | -0.89 (-0.98, -0.80) | -1.38 (-2.74, -0.01) | 1354 (984, 1779) | 57.01 (41.43, 75.18) | 2486 (1769, 3343) | 45.96 (32.55, 62.66) | -0.82 (-0.91, -0.73) | -1.26 (-1.32, -1.20) | 1661 (1199, 2189) | 64.03 (46.30, 84.45) | 3109 (2210, 4173) | 52.74 (37.47, 71.30) | -0.74 (-0.83, -0.64) | -1.12 (-1.18, -1.07) |
| 16 | East Asia | 142149 (115345, 165148) | 147.88 (119.83, 171.13) | 239026 (190370, 292221) | 89.40 (71.22, 109.09) | -1.83 (-2.07, -1.58) | -2.32 (-2.90, -1.74) | 3109240 (2520454, 3629227) | 2947.19 (2388.81, 3430.01) | 4651159 (3694864, 5717602) | 1668.26 (1326.14, 2049.12) | -2.03 (-2.25, -1.81) | -2.63 (-13.18, 7.92) | 132891 (107548, 154638) | 133.81 (108.27, 155.12) | 245951 (196279, 301256) | 90.30 (72.07, 110.45) | -1.44 (-1.66, -1.23) | -1.89 (-2.39, -1.39) | 169261 (136023, 197467) | 159.03 (127.84, 185.05) | 369610 (294859, 453915) | 131.21 (104.57, 161.03) | -0.76 (-0.94, -0.57) | -0.97 (-1.62, -0.32) |
| 17 | Eastern Europe | 8271 (7915, 8592) | 22.88 (21.82, 23.79) | 7384 (6673, 8055) | 15.29 (13.82, 16.67) | -1.50 (-1.68, -1.31) | -2.32 (-2.37, -2.26) | 181232 (174068, 188249) | 479.51 (459.79, 498.26) | 162814 (147090, 178190) | 332.14 (300.18, 363.31) | -1.41 (-1.59, -1.23) | -2.18 (-3.35, -1.00) | 7829 (7486, 8143) | 21.35 (20.36, 22.22) | 7348 (6624, 8024) | 15.15 (13.66, 16.54) | -1.29 (-1.49, -1.10) | -1.99 (-2.05, -1.94) | 10097 (9678, 10495) | 26.69 (25.54, 27.75) | 10466 (9450, 11447) | 21.33 (19.27, 23.32) | -0.87 (-1.12, -0.62) | -1.33 (-1.40, -1.26) |
| 18 | Eastern Sub-Saharan Africa | 6501 (5313, 7589) | 82.36 (67.12, 96.47) | 11790 (9741, 14282) | 69.04 (56.84, 83.72) | -0.71 (-0.77, -0.66) | -0.74 (-1.06, -0.42) | 145593 (118920, 169875) | 1681.97 (1372.94, 1965.16) | 256225 (211823, 310839) | 1377.12 (1137.08, 1669.36) | -0.81 (-0.87, -0.74) | -0.86 (-6.39, 4.68) | 5969 (4874, 6959) | 73.33 (59.74, 85.73) | 10773 (8886, 13068) | 61.35 (50.45, 74.42) | -0.72 (-0.78, -0.67) | -0.75 (-1.05, -0.46) | 7205 (5874, 8397) | 82.94 (67.57, 96.82) | 13271 (10934, 16163) | 71.04 (58.46, 86.45) | -0.62 (-0.67, -0.57) | -0.68 (-0.92, -0.44) |
| 19 | High-income Asia Pacific | 7673 (7124, 8144) | 31.15 (28.74, 33.10) | 15495 (13512, 16808) | 23.21 (20.62, 25.07) | -1.00 (-1.07, -0.93) | -1.44 (-1.47, -1.41) | 156341 (146274, 165807) | 613.70 (572.42, 651.33) | 265262 (236881, 286510) | 438.94 (397.23, 473.33) | -1.14 (-1.26, -1.01) | -1.64 (-2.18, -1.10) | 9539 (8857, 10123) | 38.18 (35.28, 40.58) | 22712 (19904, 24689) | 35.55 (31.62, 38.49) | -0.24 (-0.41, -0.07) | -0.45 (-0.49, -0.41) | 21451 (19587, 23297) | 83.98 (76.45, 91.32) | 64854 (57370, 70548) | 107.53 (96.08, 116.61) | 0.88 (0.54, 1.22) | 1.17 (1.00, 1.34) |
| 20 | High-income North America | 10141 (9476, 10582) | 21.63 (20.22, 22.57) | 19995 (18195, 21187) | 22.34 (20.38, 23.66) | -0.03 (-0.18, 0.11) | 0.08 (0.06, 0.10) | 204216 (193435, 212067) | 442.76 (419.70, 459.69) | 384691 (356926, 404739) | 435.21 (404.31, 457.64) | -0.20 (-0.33, -0.07) | -0.14 (-0.65, 0.37) | 10616 (9938, 11068) | 22.70 (21.26, 23.66) | 21977 (20034, 23253) | 24.67 (22.52, 26.08) | 0.12 (-0.05, 0.29) | 0.31 (0.29, 0.34) | 15972 (15073, 16609) | 34.48 (32.58, 35.84) | 36302 (33469, 38374) | 41.07 (37.89, 43.40) | 0.46 (0.24, 0.69) | 0.75 (0.70, 0.81) |
| 21 | North Africa and Middle East | 2916 (2361, 3392) | 16.46 (13.33, 19.20) | 6188 (5276, 7005) | 13.30 (11.30, 15.04) | -0.70 (-0.73, -0.66) | -0.71 (-0.79, -0.63) | 62397 (50394, 72452) | 321.76 (260.23, 374.11) | 122536 (104818, 139004) | 242.83 (207.41, 275.17) | -0.98 (-1.03, -0.93) | -0.98 (-2.73, 0.77) | 2661 (2153, 3089) | 14.59 (11.82, 16.98) | 5725 (4895, 6476) | 11.98 (10.21, 13.55) | -0.66 (-0.70, -0.62) | -0.65 (-0.73, -0.57) | 3416 (2766, 3950) | 17.56 (14.23, 20.33) | 8953 (7674, 10138) | 17.77 (15.19, 20.13) | -0.02 (-0.09, 0.05) | 0.03 (0.01, 0.04) |
| 22 | Oceania | 38 (28, 52) | 13.76 (10.27, 18.70) | 83 (64, 111) | 11.91 (9.12, 15.91) | -0.48 (-0.50, -0.45) | -0.80 (-0.80, -0.80) | 861 (632, 1181) | 266.85 (197.59, 363.82) | 1820 (1395, 2441) | 229.97 (176.34, 307.76) | -0.49 (-0.51, -0.46) | -0.70 (-0.87, -0.54) | 35 (26, 48) | 12.09 (8.94, 16.43) | 77 (59, 102) | 10.54 (8.05, 14.01) | -0.45 (-0.48, -0.43) | -0.76 (-0.76, -0.76) | 44 (32, 60) | 13.43 (9.89, 18.31) | 96 (73, 128) | 12.05 (9.21, 16.01) | -0.36 (-0.38, -0.33) | -0.52 (-0.53, -0.51) |
| 23 | South Asia | 13642 (11695, 16620) | 22.55 (19.18, 27.62) | 32915 (28721, 39897) | 19.57 (17.04, 23.77) | -0.72 (-0.83, -0.60) | -0.50 (-0.86, -0.14) | 302818 (261025, 367746) | 458.93 (393.47, 559.26) | 691098 (604679, 833171) | 385.83 (337.19, 466.14) | -0.84 (-0.96, -0.72) | -1.35 (-1.97, -0.72) | 12509 (10758, 15176) | 20.11 (17.17, 24.51) | 30125 (26280, 36431) | 17.53 (15.26, 21.24) | -0.71 (-0.83, -0.60) | -1.14 (-1.17, -1.11) | 15337 (13260, 18548) | 23.13 (19.89, 28.06) | 37247 (32573, 44909) | 20.71 (18.09, 25.02) | -0.63 (-0.74, -0.51) | -0.95 (-0.98, -0.92) |
| 24 | Southeast Asia | 4336 (3568, 5177) | 15.79 (13.00, 18.84) | 10279 (8666, 12109) | 13.90 (11.70, 16.37) | -0.52 (-0.56, -0.47) | -0.59 (-0.63, -0.55) | 94719 (77802, 113205) | 320.48 (263.44, 382.66) | 219077 (184954, 258619) | 274.84 (231.97, 324.07) | -0.60 (-0.65, -0.56) | -0.68 (-1.43, 0.07) | 3997 (3288, 4787) | 14.22 (11.71, 17.02) | 9862 (8298, 11618) | 13.03 (10.94, 15.33) | -0.38 (-0.43, -0.34) | -0.55 (-0.57, -0.53) | 4998 (4111, 5994) | 16.87 (13.89, 20.22) | 13439 (11244, 15894) | 16.85 (14.09, 19.90) | -0.09 (-0.13, -0.05) | -0.00 (-0.08, 0.08) |
| 25 | Southern Latin America | 2736 (2434, 3050) | 48.51 (42.99, 54.10) | 3068 (2640, 3489) | 26.74 (23.03, 30.42) | -1.91 (-2.13, -1.69) | -2.12 (-2.34, -1.90) | 52879 (47268, 58947) | 901.95 (804.66, 1005.74) | 55308 (47988, 62908) | 488.56 (424.05, 555.81) | -2.01 (-2.23, -1.78) | -2.21 (-6.32, 1.89) | 2458 (2188, 2738) | 42.96 (38.13, 47.87) | 2800 (2418, 3184) | 24.51 (21.17, 27.87) | -1.82 (-2.04, -1.59) | -1.99 (-2.19, -1.79) | 2893 (2588, 3217) | 49.19 (43.93, 54.72) | 3420 (2971, 3891) | 30.22 (26.26, 34.38) | -1.59 (-1.82, -1.37) | -1.71 (-1.90, -1.52) |
| 26 | Southern Sub-Saharan Africa | 1805 (1535, 2175) | 60.97 (51.72, 73.62) | 4095 (3609, 4603) | 64.71 (56.72, 72.87) | -0.14 (-0.62, 0.35) | 0.42 (0.08, 0.76) | 37336 (31824, 44832) | 1178.57 (1002.95, 1417.21) | 86714 (76684, 97379) | 1262.90 (1113.82, 1419.41) | -0.12 (-0.62, 0.38) | 0.46 (-6.68, 7.60) | 1625 (1382, 1953) | 53.67 (45.58, 64.65) | 3740 (3299, 4204) | 57.52 (50.49, 64.75) | -0.11 (-0.59, 0.38) | 0.45 (0.15, 0.76) | 1961 (1669, 2362) | 61.77 (52.50, 74.52) | 4514 (3996, 5070) | 65.51 (57.83, 73.62) | -0.15 (-0.60, 0.30) | 0.43 (0.05, 0.80) |
| 27 | Tropical Latin America | 3915 (3637, 4151) | 38.78 (35.71, 41.25) | 8822 (7954, 9486) | 27.79 (24.99, 29.90) | -1.00 (-1.06, -0.94) | -1.50 (-1.54, -1.46) | 82750 (77393, 87545) | 765.10 (712.13, 810.86) | 181661 (165818, 194361) | 560.03 (510.40, 599.49) | -0.98 (-1.04, -0.92) | -1.42 (-2.29, -0.54) | 3563 (3312, 3775) | 34.49 (31.82, 36.63) | 8115 (7344, 8713) | 25.40 (22.93, 27.29) | -0.93 (-0.99, -0.87) | -1.39 (-1.42, -1.35) | 4364 (4074, 4610) | 40.23 (37.39, 42.58) | 10350 (9454, 11079) | 31.91 (29.10, 34.17) | -0.72 (-0.78, -0.67) | -1.04 (-1.08, -0.99) |
| 28 | Western Europe | 20808 (19409, 21862) | 26.99 (25.15, 28.38) | 29373 (26109, 31573) | 22.81 (20.54, 24.43) | -0.59 (-0.65, -0.52) | -0.97 (-1.00, -0.95) | 401610 (378402, 420995) | 529.38 (498.81, 555.08) | 522556 (474249, 558557) | 439.84 (402.91, 468.92) | -0.63 (-0.69, -0.57) | -1.05 (-1.49, -0.61) | 20025 (18714, 21031) | 26.03 (24.32, 27.35) | 31402 (27967, 33840) | 25.13 (22.64, 26.99) | -0.10 (-0.16, -0.04) | 0.06 (-0.17, 0.28) | 27245 (25614, 28650) | 35.80 (33.67, 37.64) | 56051 (50236, 60766) | 47.52 (42.90, 51.39) | 1.11 (0.97, 1.25) | 1.64 (1.54, 1.74) |
| 29 | Western Sub-Saharan Africa | 1637 (1333, 1972) | 17.00 (13.89, 20.41) | 5545 (4110, 6625) | 28.08 (20.81, 33.42) | 2.22 (2.00, 2.45) | 2.55 (2.29, 2.82) | 35307 (28615, 42785) | 342.23 (278.06, 413.47) | 117394 (86998, 140860) | 548.81 (406.63, 656.37) | 2.10 (1.89, 2.32) | 2.43 (-3.95, 8.81) | 1487 (1207, 1792) | 15.09 (12.28, 18.12) | 5031 (3714, 6013) | 24.83 (18.34, 29.58) | 2.20 (1.98, 2.43) | 2.53 (2.30, 2.76) | 1782 (1438, 2155) | 17.21 (13.93, 20.76) | 6094 (4481, 7316) | 28.39 (20.88, 33.98) | 2.21 (1.98, 2.43) | 2.54 (2.29, 2.79) |
| Stomach cancer |  |  |  |  |  |  |  |  |  |  |  |  |  |  |  |  |  |  |  |  |  |  |  |  |  |
| 1 | Global | 580146 (524548, 642458) | 126.09 (113.66, 139.54) | 732426 (620849, 834822) | 69.10 (58.48, 78.69) | -2.00 (-2.08, -1.92) | -3.565790013 | 11928996 (10784197, 13214086) | 2456.62 (2218.72, 2720.79) | 13797102 (11753648, 15790412) | 1272.06 (1082.71, 1454.91) | -2.19 (-2.27, -2.11) | -3.93 (-5.23, -2.62) | 630551 (572742, 691697) | 134.56 (121.86, 147.56) | 896397 (757409, 1025419) | 83.82 (70.74, 95.79) | -1.55 (-1.61, -1.49) | -2.21 (-2.36, -2.05) | 939500 (862455, 1015822) | 193.97 (177.66, 209.72) | 1568205 (1330519, 1797897) | 144.51 (122.53, 165.54) | -0.91 (-1.00, -0.82) | -1.30 (-1.53, -1.07) |
| 2 | Male | 352829 (306593, 406853) | 175.83 (153.34, 202.63) | 472894 (383086, 561960) | 100.20 (81.18, 118.58) | -1.84 (-1.93, -1.74) | -2.55 (-2.83, -2.27) | 7533249 (6508564, 8677065) | 3451.67 (2991.49, 3978.60) | 9210170 (7469152, 11013181) | 1851.10 (1501.20, 2207.51) | -2.04 (-2.13, -1.94) | -2.80 (-8.10, 2.50) | 389696 (342313, 443858) | 188.86 (166.28, 215.08) | 599149 (486200, 712874) | 124.30 (100.89, 147.31) | -1.33 (-1.41, -1.24) | -1.93 (-2.14, -1.71) | 598850 (536535, 665799) | 275.78 (247.49, 306.42) | 1105192 (907480, 1315142) | 221.72 (182.27, 262.97) | -0.62 (-0.73, -0.51) | -1.08 (-1.35, -0.81) |
| 3 | Female | 227316 (202419, 249964) | 88.22 (78.15, 97.03) | 259532 (218157, 295675) | 44.03 (37.04, 50.15) | -2.39 (-2.47, -2.30) | -4.02 (-4.06, -3.98) | 4395747 (3944366, 4843098) | 1651.06 (1477.43, 1819.15) | 4586932 (3940654, 5216252) | 780.30 (670.68, 887.19) | -2.58 (-2.67, -2.50) | -4.31 (-5.13, -3.49) | 240855 (215290, 262947) | 92.35 (82.17, 100.88) | 297248 (248685, 339963) | 50.47 (42.25, 57.71) | -2.07 (-2.13, -2.00) | -3.53 (-3.56, -3.49) | 340650 (306981, 368881) | 127.90 (114.92, 138.60) | 463013 (389426, 530538) | 78.75 (66.26, 90.22) | -1.63 (-1.70, -1.55) | -2.98 (-3.07, -2.90) |
| 4 | High SDI | 138277 (128125, 144618) | 95.48 (88.22, 99.97) | 135711 (116751, 148458) | 44.66 (38.91, 48.70) | -2.55 (-2.59, -2.51) | -3.963670701 | 2562346 (2406241, 2668569) | 1767.85 (1658.36, 1842.01) | 2180274 (1939543, 2369441) | 759.70 (682.37, 824.03) | -2.84 (-2.88, -2.79) | -4.43 (-5.14, -3.73) | 186201 (173298, 194513) | 128.13 (119.07, 133.95) | 202310 (175838, 221159) | 68.37 (60.09, 74.51) | -2.10 (-2.18, -2.02) | -2.29 (-2.67, -1.90) | 384135 (359639, 402399) | 264.06 (247.22, 276.62) | 469114 (414162, 511337) | 164.55 (146.38, 178.98) | -1.55 (-1.70, -1.39) | -2.23 (-2.40, -2.06) |
| 5 | High-middle SDI | 209285 (187825, 228829) | 175.12 (156.63, 191.57) | 232650 (190630, 273106) | 92.15 (75.44, 108.05) | -2.13 (-2.24, -2.01) | -3.748885082 | 4350172 (3907326, 4759420) | 3447.63 (3091.79, 3773.12) | 4415933 (3631647, 5210847) | 1721.22 (1414.86, 2029.25) | -2.31 (-2.42, -2.20) | -4.19 (-7.03, -1.35) | 215527 (193874, 235480) | 176.71 (158.52, 193.10) | 285171 (231180, 338899) | 111.98 (90.74, 132.92) | -1.46 (-1.55, -1.37) | -2.08 (-2.45, -1.71) | 277697 (250509, 302645) | 219.64 (197.81, 239.46) | 484405 (389646, 585576) | 187.82 (150.99, 226.78) | -0.38 (-0.49, -0.26) | -0.52 (-1.08, 0.04) |
| 6 | Middle SDI | 178969 (154917, 212121) | 160.36 (139.05, 190.23) | 266052 (217519, 315275) | 85.48 (69.74, 101.09) | -2.10 (-2.23, -1.97) | -2.438926477 | 3848985 (3320611, 4549753) | 3176.27 (2745.70, 3759.85) | 5179817 (4247409, 6156483) | 1581.62 (1295.53, 1877.08) | -2.31 (-2.43, -2.19) | -3.15 (-10.78, 4.48) | 178357 (153980, 211227) | 154.73 (133.85, 183.48) | 315137 (253592, 378290) | 98.73 (79.35, 118.25) | -1.46 (-1.56, -1.36) | -2.04 (-2.33, -1.76) | 220529 (189961, 260432) | 180.73 (155.99, 213.72) | 503927 (400329, 614713) | 151.59 (120.42, 184.56) | -0.49 (-0.58, -0.41) | -0.66 (-1.04, -0.29) |
| 7 | Low-middle SDI | 37314 (32481, 46356) | 58.04 (50.46, 71.82) | 71793 (62093, 81916) | 45.03 (38.81, 51.28) | -0.73 (-0.80, -0.66) | -1.192139315 | 808620 (703749, 1006967) | 1152.11 (1002.62, 1431.99) | 1473332 (1277567, 1687604) | 865.05 (748.59, 989.42) | -0.87 (-0.93, -0.80) | -1.43 (-2.14, -0.71) | 35160 (30585, 43720) | 53.08 (46.14, 65.81) | 69096 (59705, 79130) | 42.31 (36.42, 48.35) | -0.66 (-0.72, -0.59) | -1.10 (-1.14, -1.07) | 40096 (34935, 49819) | 57.23 (49.86, 70.95) | 82697 (71633, 94961) | 48.50 (41.92, 55.59) | -0.47 (-0.54, -0.41) | -0.81 (-0.85, -0.77) |
| 8 | Low SDI | 15771 (12602, 18409) | 66.69 (53.28, 78.04) | 25739 (20339, 29593) | 50.13 (39.63, 57.63) | -0.85 (-0.91, -0.79) | -1.150790753 | 348495 (278157, 406363) | 1338.54 (1068.78, 1563.05) | 538693 (425128, 620078) | 961.24 (759.17, 1106.01) | -1.04 (-1.09, -1.00) | -1.55 (-3.00, -0.10) | 14790 (11811, 17263) | 60.53 (48.35, 70.84) | 24190 (19034, 27801) | 45.69 (35.96, 52.51) | -0.85 (-0.90, -0.80) | -1.26 (-1.34, -1.18) | 16420 (13080, 19129) | 63.11 (50.31, 73.66) | 27383 (21456, 31485) | 48.80 (38.27, 56.09) | -0.79 (-0.84, -0.74) | -1.17 (-1.24, -1.09) |
| 9 | Andean Latin America | 4603 (3947, 5378) | 204.21 (174.83, 238.80) | 9542 (7510, 11938) | 135.49 (106.68, 169.43) | -1.52 (-1.68, -1.37) | 0.4925125669866 | 88515 (76079, 103288) | 3784.13 (3250.41, 4417.54) | 173551 (136252, 217997) | 2429.62 (1908.24, 3050.48) | -1.65 (-1.79, -1.51) | -2.83 (-7.75, 2.08) | 4210 (3613, 4920) | 184.45 (158.08, 215.71) | 9132 (7177, 11433) | 129.05 (101.45, 161.49) | -1.36 (-1.51, -1.21) | -1.25 (-3.10, 0.59) | 4702 (4035, 5508) | 201.86 (173.03, 236.56) | 12000 (9352, 15222) | 168.84 (131.56, 214.23) | -0.73 (-0.87, -0.59) | -1.17 (-1.48, -0.86) |
| 10 | Australasia | 1503 (1348, 1654) | 49.85 (44.50, 55.08) | 1899 (1590, 2185) | 25.06 (21.10, 28.76) | -2.24 (-2.37, -2.11) | -3.420569752 | 27410 (24839, 30004) | 888.00 (802.35, 973.95) | 30794 (26366, 35092) | 424.60 (365.33, 482.71) | -2.44 (-2.55, -2.33) | -3.77 (-4.48, -3.07) | 1911 (1709, 2117) | 62.46 (55.67, 69.36) | 2626 (2203, 3065) | 35.41 (29.83, 41.26) | -1.91 (-2.00, -1.82) | -3.03 (-3.08, -2.98) | 3436 (3004, 3898) | 110.27 (96.27, 125.22) | 5600 (4570, 6693) | 77.74 (63.60, 92.82) | -1.24 (-1.35, -1.13) | -1.43 (-1.86, -1.00) |
| 11 | Caribbean | 2312 (2077, 2572) | 75.04 (67.28, 83.47) | 3065 (2596, 3570) | 45.42 (38.48, 52.89) | -1.55 (-1.61, -1.49) | -2.177127703 | 44240 (39572, 49575) | 1388.15 (1240.56, 1555.43) | 58055 (49130, 67927) | 864.93 (732.12, 1012.09) | -1.48 (-1.56, -1.41) | -2.29 (-3.40, -1.17) | 2186 (1966, 2427) | 69.91 (62.77, 77.65) | 2963 (2518, 3444) | 44.02 (37.43, 51.17) | -1.43 (-1.49, -1.37) | -2.20 (-2.26, -2.14) | 2550 (2301, 2827) | 79.78 (71.92, 88.47) | 3778 (3229, 4379) | 56.33 (48.16, 65.30) | -1.06 (-1.11, -1.01) | -1.71 (-1.77, -1.64) |
| 12 | Central Asia | 7718 (7207, 8263) | 140.75 (131.04, 150.90) | 6138 (5504, 6846) | 67.21 (60.27, 74.77) | -2.06 (-2.19, -1.93) | -2.000868242 | 168183 (158045, 179640) | 2938.12 (2754.79, 3141.83) | 133872 (120085, 149792) | 1360.21 (1221.55, 1518.15) | -2.21 (-2.32, -2.11) | -2.94 (-13.48, 7.60) | 7430 (6946, 7939) | 133.85 (124.79, 143.19) | 6090 (5460, 6801) | 65.22 (58.50, 72.65) | -1.99 (-2.12, -1.86) | -2.71 (-3.29, -2.13) | 8854 (8312, 9452) | 155.29 (145.50, 165.93) | 7610 (6812, 8503) | 77.55 (69.54, 86.46) | -1.90 (-2.05, -1.76) | -2.69 (-3.30, -2.08) |
| 13 | Central Europe | 20926 (19825, 21865) | 112.64 (106.22, 117.91) | 16250 (14810, 17613) | 53.01 (48.32, 57.46) | -2.57 (-2.66, -2.48) | -4.299049867 | 415450 (394825, 433956) | 2137.70 (2027.06, 2234.98) | 303754 (278351, 329369) | 1003.64 (919.90, 1088.34) | -2.57 (-2.66, -2.49) | -4.34 (-5.40, -3.29) | 20111 (19036, 21029) | 106.51 (100.46, 111.54) | 16614 (15120, 18036) | 54.35 (49.48, 59.01) | -2.28 (-2.37, -2.19) | -4.06 (-4.12, -4.00) | 23561 (22310, 24714) | 121.13 (114.47, 127.15) | 22346 (20318, 24478) | 73.61 (66.95, 80.63) | -1.64 (-1.76, -1.53) | -2.16 (-2.35, -1.98) |
| 14 | Central Latin America | 11712 (10997, 12328) | 133.18 (124.39, 140.34) | 20835 (18227, 23421) | 69.98 (61.20, 78.63) | -2.35 (-2.43, -2.27) | -3.83686626 | 222788 (210295, 234315) | 2387.43 (2247.02, 2512.05) | 389299 (342304, 438220) | 1275.42 (1121.18, 1435.11) | -2.33 (-2.42, -2.24) | -3.88 (-5.41, -2.34) | 10862 (10200, 11423) | 120.89 (113.01, 127.25) | 20139 (17665, 22675) | 67.12 (58.85, 75.53) | -2.20 (-2.28, -2.11) | -3.69 (-3.76, -3.61) | 12817 (12057, 13491) | 137.17 (128.67, 144.45) | 27302 (23933, 30947) | 89.63 (78.55, 101.58) | -1.67 (-1.77, -1.57) | -2.91 (-3.02, -2.80) |
| 15 | Central Sub-Saharan Africa | 1538 (1112, 1965) | 70.58 (50.81, 90.14) | 2675 (1945, 3457) | 53.22 (38.72, 69.06) | -0.96 (-0.99, -0.92) | -1.437186085 | 34611 (25054, 44373) | 1387.87 (1001.78, 1775.75) | 58520 (42491, 75689) | 1028.10 (747.35, 1330.55) | -1.01 (-1.05, -0.98) | -1.57 (-2.64, -0.51) | 1451 (1048, 1857) | 63.63 (45.76, 81.39) | 2530 (1844, 3267) | 48.33 (35.14, 62.58) | -0.94 (-0.97, -0.90) | -1.45 (-1.49, -1.40) | 1616 (1171, 2079) | 64.94 (46.89, 83.35) | 2904 (2114, 3750) | 51.11 (37.16, 66.06) | -0.81 (-0.85, -0.77) | -1.24 (-1.28, -1.20) |
| 16 | East Asia | 241594 (203856, 290486) | 255.37 (215.59, 308.66) | 351490 (270199, 435861) | 132.47 (101.68, 163.69) | -2.15 (-2.38, -1.92) | -1.998072392 | 5228047 (4389918, 6265468) | 5008.54 (4213.08, 6024.76) | 6775821 (5213614, 8461438) | 2441.25 (1877.21, 3042.26) | -2.36 (-2.57, -2.14) | -3.20 (-19.51, 13.11) | 245112 (206529, 294178) | 248.82 (209.79, 300.05) | 447492 (343037, 557004) | 164.30 (125.74, 203.94) | -1.30 (-1.49, -1.10) | -1.74 (-2.49, -1.00) | 308694 (258661, 369408) | 292.06 (245.09, 350.59) | 777319 (590670, 978515) | 274.91 (208.63, 345.56) | -0.07 (-0.27, 0.13) | -0.05 (-1.11, 1.02) |
| 17 | Eastern Europe | 57564 (55197, 59514) | 159.88 (152.81, 165.54) | 33825 (30770, 36679) | 71.37 (64.92, 77.37) | -2.78 (-2.86, -2.70) | -4.385148961 | 1235086 (1189974, 1274663) | 3308.39 (3182.09, 3417.61) | 677540 (617320, 735007) | 1406.66 (1282.10, 1525.30) | -3.00 (-3.10, -2.90) | -4.89 (-8.42, -1.35) | 60993 (58457, 63196) | 167.43 (160.07, 173.71) | 38680 (35192, 41935) | 81.26 (73.93, 88.09) | -2.49 (-2.57, -2.41) | -3.85 (-4.12, -3.59) | 80610 (77320, 83805) | 216.10 (206.94, 224.89) | 57965 (52685, 63135) | 120.23 (109.28, 130.94) | -1.96 (-2.07, -1.85) | -2.91 (-3.39, -2.44) |
| 18 | Eastern Sub-Saharan Africa | 4822 (3831, 5664) | 61.82 (49.15, 72.85) | 6986 (5766, 8196) | 42.23 (34.85, 49.60) | -1.41 (-1.48, -1.34) | -0.960891726 | 106291 (84203, 124547) | 1241.55 (984.79, 1458.06) | 146937 (120819, 172244) | 809.58 (666.86, 949.52) | -1.58 (-1.65, -1.51) | -1.60 (-12.04, 8.84) | 4521 (3580, 5330) | 56.16 (44.51, 66.34) | 6550 (5386, 7693) | 38.37 (31.54, 45.13) | -1.42 (-1.49, -1.35) | -1.44 (-1.90, -0.97) | 4939 (3907, 5804) | 57.83 (45.81, 68.08) | 7388 (6068, 8662) | 40.71 (33.48, 47.79) | -1.31 (-1.37, -1.25) | -2.29 (-2.32, -2.26) |
| 19 | High-income Asia Pacific | 53346 (48790, 56464) | 221.80 (201.32, 235.44) | 65386 (54292, 72536) | 88.16 (75.13, 97.10) | -3.06 (-3.10, -3.01) | -4.327303665 | 1027015 (946902, 1084037) | 4094.79 (3759.80, 4328.88) | 979893 (842270, 1078705) | 1490.10 (1307.31, 1632.89) | -3.33 (-3.38, -3.28) | -4.82 (-8.33, -1.32) | 88835 (81917, 93953) | 359.43 (329.61, 381.00) | 109237 (91923, 121041) | 157.65 (135.75, 173.57) | -2.72 (-2.78, -2.65) | -3.86 (-4.33, -3.39) | 230067 (212975, 244004) | 904.01 (834.37, 960.22) | 282603 (242341, 313071) | 443.05 (386.01, 488.49) | -2.31 (-2.40, -2.22) | -2.79 (-4.89, -0.68) |
| 20 | High-income North America | 16376 (14938, 17252) | 34.57 (31.51, 36.43) | 15922 (14031, 17066) | 17.56 (15.54, 18.80) | -2.32 (-2.38, -2.26) | -3.691748256 | 295530 (275210, 309511) | 630.41 (587.30, 660.16) | 279364 (253255, 296601) | 313.36 (284.80, 332.35) | -2.40 (-2.46, -2.33) | -3.81 (-4.11, -3.51) | 23001 (21083, 24249) | 48.56 (44.49, 51.20) | 25730 (22894, 27541) | 28.64 (25.55, 30.63) | -1.85 (-1.92, -1.78) | -1.97 (-2.32, -1.63) | 46112 (42547, 48840) | 97.64 (90.13, 103.40) | 62704 (56600, 67133) | 70.59 (63.78, 75.55) | -1.13 (-1.27, -0.99) | -1.41 (-1.54, -1.29) |
| 21 | North Africa and Middle East | 15506 (11724, 18057) | 88.80 (67.37, 103.80) | 27346 (19186, 31508) | 59.09 (41.65, 68.09) | -1.16 (-1.24, -1.08) | -1.535151659 | 327140 (246249, 379927) | 1704.72 (1287.34, 1983.86) | 535951 (373708, 617926) | 1067.94 (747.05, 1231.09) | -1.42 (-1.48, -1.35) | -2.02 (-4.37, 0.33) | 14684 (11038, 17077) | 81.36 (61.33, 94.93) | 27449 (19054, 31710) | 57.51 (40.05, 66.43) | -0.98 (-1.06, -0.90) | -1.42 (-1.54, -1.29) | 17252 (12828, 20006) | 89.35 (66.72, 103.85) | 36821 (25277, 42872) | 72.05 (49.62, 83.81) | -0.61 (-0.69, -0.52) | -0.89 (-1.05, -0.73) |
| 22 | Oceania | 276 (197, 360) | 101.74 (73.89, 131.55) | 528 (398, 692) | 77.96 (59.06, 101.76) | -0.87 (-0.93, -0.81) | -1.27250487 | 6106 (4305, 8005) | 1938.19 (1387.06, 2522.97) | 11177 (8382, 14695) | 1457.77 (1098.56, 1910.38) | -0.92 (-1.00, -0.85) | -1.40 (-2.10, -0.69) | 263 (186, 344) | 91.76 (66.10, 119.12) | 508 (384, 667) | 71.78 (54.46, 93.73) | -0.82 (-0.87, -0.76) | -1.23 (-1.27, -1.20) | 300 (211, 395) | 95.07 (67.81, 124.25) | 597 (450, 783) | 77.59 (58.68, 101.59) | -0.69 (-0.75, -0.63) | -1.05 (-1.09, -1.00) |
| 23 | South Asia | 25617 (21548, 33638) | 41.96 (35.15, 55.24) | 55234 (46796, 66102) | 32.95 (27.80, 39.39) | -0.68 (-0.77, -0.58) | -0.763759516 | 576908 (486858, 754579) | 865.54 (728.17, 1135.26) | 1152981 (982361, 1383630) | 645.17 (548.23, 773.55) | -0.89 (-0.97, -0.81) | -1.23 (-3.51, 1.04) | 24322 (20504, 31991) | 38.68 (32.46, 51.02) | 53111 (45109, 63606) | 30.95 (26.18, 37.03) | -0.64 (-0.73, -0.55) | -0.88 (-1.00, -0.76) | 27879 (23573, 36572) | 41.82 (35.24, 55.01) | 62309 (53047, 74800) | 34.82 (29.56, 41.75) | -0.54 (-0.63, -0.45) | -0.75 (-0.86, -0.64) |
| 24 | Southeast Asia | 16628 (13405, 19613) | 61.66 (49.82, 73.08) | 28794 (24609, 35274) | 39.98 (34.10, 48.88) | -1.62 (-1.70, -1.54) | -2.725169317 | 356953 (286764, 419076) | 1223.14 (984.95, 1440.55) | 594352 (508383, 729695) | 761.44 (651.11, 933.79) | -1.76 (-1.84, -1.68) | -2.95 (-3.71, -2.19) | 15773 (12689, 18578) | 56.91 (45.87, 67.28) | 29476 (25138, 36170) | 39.68 (33.79, 48.59) | -1.40 (-1.48, -1.32) | -2.40 (-2.44, -2.36) | 18317 (14710, 21510) | 62.72 (50.48, 73.86) | 39077 (33119, 47586) | 49.80 (42.20, 60.61) | -1.00 (-1.08, -0.91) | -1.67 (-1.72, -1.61) |
| 25 | Southern Latin America | 6589 (6009, 7194) | 116.68 (105.91, 127.68) | 7590 (6575, 8522) | 66.22 (57.40, 74.33) | -1.66 (-1.76, -1.56) | -1.544103387 | 126351 (115879, 137515) | 2155.06 (1971.29, 2348.55) | 137503 (120558, 154043) | 1215.50 (1066.24, 1361.76) | -1.71 (-1.81, -1.61) | -2.03 (-8.63, 4.57) | 6363 (5811, 6950) | 110.96 (100.95, 121.44) | 7739 (6725, 8723) | 67.80 (58.95, 76.42) | -1.44 (-1.55, -1.33) | -1.67 (-2.06, -1.28) | 7404 (6759, 8095) | 125.92 (114.69, 137.83) | 9977 (8670, 11321) | 88.17 (76.64, 100.04) | -0.99 (-1.11, -0.87) | -1.16 (-1.49, -0.84) |
| 26 | Southern Sub-Saharan Africa | 1311 (1022, 1521) | 45.34 (35.25, 52.68) | 2548 (2115, 2912) | 41.21 (34.12, 47.02) | -0.40 (-0.75, -0.06) | 0.137306574596142 | 25949 (20279, 30067) | 836.39 (652.65, 970.10) | 52220 (43413, 59797) | 776.03 (644.09, 887.34) | -0.30 (-0.66, 0.06) | -0.02 (-4.14, 4.09) | 1218 (946, 1409) | 41.13 (31.83, 47.61) | 2430 (2014, 2779) | 38.16 (31.54, 43.56) | -0.33 (-0.67, 0.00) | -0.06 (-0.25, 0.12) | 1348 (1052, 1555) | 43.50 (33.87, 50.23) | 2746 (2275, 3138) | 40.78 (33.71, 46.54) | -0.29 (-0.57, 0.00) | 0.02 (-0.19, 0.23) |
| 27 | Tropical Latin America | 11593 (10777, 12252) | 118.95 (109.42, 126.25) | 18275 (16454, 19637) | 58.40 (52.42, 62.81) | -2.35 (-2.41, -2.29) | -3.769516087 | 232056 (217464, 244399) | 2206.14 (2054.44, 2329.21) | 349388 (320424, 373374) | 1090.96 (998.14, 1166.69) | -2.36 (-2.42, -2.30) | -3.88 (-5.10, -2.65) | 10931 (10171, 11550) | 109.13 (100.62, 115.72) | 17692 (15982, 18995) | 56.11 (50.53, 60.31) | -2.22 (-2.28, -2.16) | -3.65 (-3.71, -3.59) | 12621 (11799, 13323) | 119.96 (111.44, 126.92) | 22390 (20417, 23986) | 70.01 (63.68, 75.06) | -1.84 (-1.90, -1.78) | -3.10 (-3.17, -3.04) |
| 28 | Western Europe | 74104 (68416, 78032) | 94.62 (87.15, 99.73) | 49994 (42827, 54421) | 36.16 (31.51, 39.12) | -3.13 (-3.24, -3.03) | -4.714362138 | 1289944 (1208823, 1349797) | 1663.64 (1558.82, 1741.06) | 789692 (696391, 851447) | 624.36 (558.84, 669.52) | -3.18 (-3.28, -3.08) | -4.86 (-6.20, -3.51) | 82198 (76028, 86644) | 104.81 (96.87, 110.53) | 62654 (54221, 68476) | 47.28 (41.54, 51.40) | -2.55 (-2.60, -2.49) | -3.81 (-3.88, -3.74) | 121863 (112423, 129707) | 156.13 (144.16, 166.14) | 116351 (101620, 128315) | 93.70 (82.74, 102.93) | -1.47 (-1.55, -1.40) | -1.60 (-3.45, 0.25) |
| 29 | Western Sub-Saharan Africa | 4506 (3842, 5455) | 48.25 (41.12, 58.80) | 8105 (6379, 9627) | 42.31 (33.70, 50.27) | -0.19 (-0.27, -0.10) | 0.0212537322873694 | 94426 (80428, 113726) | 934.06 (795.88, 1129.81) | 166438 (129252, 197927) | 795.11 (623.56, 945.39) | -0.30 (-0.38, -0.22) | -0.36 (-5.24, 4.52) | 4177 (3542, 5069) | 43.53 (36.90, 53.12) | 7556 (5930, 8966) | 38.30 (30.39, 45.45) | -0.18 (-0.27, -0.10) | -0.24 (-0.47, -0.01) | 4557 (3861, 5514) | 45.10 (38.22, 54.78) | 8419 (6545, 9991) | 40.24 (31.58, 47.74) | -0.15 (-0.23, -0.06) | -0.21 (-0.43, 0.02) |
| Colon and rectum cancer |  |  |  |  |  |  |  |  |  |  |  |  |  |  |  |  |  |  |  |  |  |  |  |  |  |
| 1 | Global | 426624 (395396, 448106) | 98.31 (90.04, 103.60) | 827471 (736233, 897443) | 79.10 (70.05, 85.87) | -0.77 (-0.81, -0.74) | -0.76 (-1.34, -0.18) | 8303284 (7773030, 8709793) | 1777.10 (1652.18, 1867.51) | 15347705 (13913323, 16592781) | 1427.86 (1290.41, 1544.63) | -0.79 (-0.82, -0.75) | -0.77 (-12.00, 10.47) | 655024 (611558, 683892) | 144.74 (133.81, 151.59) | 1617011 (1447212, 1754348) | 151.61 (135.19, 164.60) | 0.09 (0.05, 0.14) | 0.22 (-0.08, 0.53) | 2866157 (2684899, 3047785) | 612.23 (569.71, 655.33) | 8079970 (7332761, 8727386) | 747.36 (676.91, 807.55) | 0.67 (0.62, 0.73) | 1.02 (0.85, 1.20) |
| 2 | Male | 208134 (194606, 220051) | 113.02 (104.83, 119.55) | 452066 (405405, 499298) | 98.65 (88.03, 108.85) | -0.47 (-0.50, -0.44) | -0.60 (-0.70, -0.50) | 4241808 (3980130, 4490616) | 2061.83 (1927.10, 2181.99) | 8752401 (7907684, 9678638) | 1792.97 (1615.44, 1980.95) | -0.50 (-0.53, -0.46) | -0.62 (-2.86, 1.62) | 325455 (307255, 340769) | 166.43 (155.94, 174.51) | 915928 (821925, 1015889) | 191.06 (170.86, 211.64) | 0.43 (0.38, 0.48) | 0.65 (0.56, 0.73) | 1413455 (1333102, 1502830) | 687.59 (643.62, 737.81) | 4593993 (4156112, 5073206) | 929.39 (840.09, 1025.13) | 1.05 (0.98, 1.12) | 1.64 (1.45, 1.83) |
| 3 | Female | 218490 (197946, 233571) | 87.48 (78.49, 93.76) | 375404 (319057, 414251) | 63.59 (54.11, 70.15) | -1.14 (-1.19, -1.10) | -1.98 (-2.01, -1.94) | 4061475 (3729075, 4334901) | 1554.01 (1418.59, 1661.06) | 6595305 (5758706, 7235123) | 1121.60 (979.94, 1230.13) | -1.18 (-1.23, -1.14) | -2.02 (-2.65, -1.40) | 329568 (300052, 349692) | 128.19 (115.82, 136.35) | 701084 (598430, 777282) | 119.12 (101.73, 132.03) | -0.35 (-0.39, -0.30) | -0.28 (-0.58, 0.03) | 1452702 (1337566, 1563315) | 552.57 (506.50, 597.33) | 3485977 (3043641, 3834593) | 593.31 (518.10, 652.56) | 0.20 (0.16, 0.25) | 0.31 (0.02, 0.60) |
| 4 | High SDI | 204700 (188610, 214311) | 142.27 (130.55, 149.21) | 295937 (256009, 319786) | 97.58 (85.56, 104.88) | -1.33 (-1.38, -1.29) | -2.31 (-2.38, -2.24) | 3759634 (3527815, 3919448) | 2596.82 (2432.74, 2709.08) | 5026828 (4497558, 5380177) | 1759.79 (1590.58, 1875.83) | -1.38 (-1.43, -1.33) | -1.37 (-19.43, 16.69) | 374967 (347497, 391824) | 258.01 (238.68, 269.82) | 681335 (602603, 729835) | 236.21 (210.98, 252.07) | -0.40 (-0.51, -0.29) | -0.26 (-0.88, 0.35) | 1855217 (1732651, 1970666) | 1272.96 (1188.18, 1354.04) | 3795170 (3440572, 4033031) | 1349.70 (1230.62, 1430.63) | 0.15 (0.01, 0.28) | 0.25 (-1.17, 1.68) |
| 5 | High-middle SDI | 126325 (117536, 133275) | 110.45 (101.83, 116.81) | 251252 (222177, 277814) | 100.44 (88.60, 111.08) | -0.34 (-0.43, -0.26) | -0.42 (-0.54, -0.30) | 2527645 (2371702, 2666917) | 2059.38 (1922.42, 2175.07) | 4716083 (4217492, 5227529) | 1849.41 (1651.52, 2050.11) | -0.41 (-0.49, -0.34) | -0.51 (-3.07, 2.05) | 173066 (161318, 182190) | 144.90 (134.14, 152.85) | 495047 (436107, 553210) | 194.69 (171.26, 217.55) | 0.99 (0.92, 1.05) | 1.53 (1.44, 1.62) | 677411 (630162, 729440) | 547.18 (506.31, 592.98) | 2448689 (2172047, 2741683) | 952.89 (844.87, 1066.63) | 1.97 (1.88, 2.06) | 3.18 (2.44, 3.92) |
| 6 | Middle SDI | 64952 (58064, 71967) | 62.05 (55.36, 68.57) | 198694 (173341, 223158) | 64.59 (56.12, 72.53) | 0.09 (0.05, 0.13) | 0.19 (-0.10, 0.47) | 1354661 (1210773, 1504574) | 1163.40 (1039.28, 1289.18) | 3925558 (3440949, 4411350) | 1206.41 (1055.03, 1355.39) | 0.10 (0.06, 0.13) | 0.21 (-0.27, 0.69) | 75385 (67371, 83508) | 68.18 (60.86, 75.38) | 345887 (299040, 393939) | 107.86 (93.05, 122.77) | 1.55 (1.48, 1.61) | 2.14 (2.01, 2.27) | 247688 (221160, 277449) | 208.51 (185.84, 234.25) | 1533091 (1328487, 1746997) | 460.19 (398.85, 523.95) | 2.83 (2.71, 2.94) | 3.72 (2.99, 4.45) |
| 7 | Low-middle SDI | 20253 (17321, 23094) | 32.10 (27.43, 36.59) | 59805 (54171, 65829) | 37.77 (34.03, 41.63) | 0.56 (0.53, 0.60) | 0.83 (0.80, 0.87) | 435727 (372719, 497341) | 628.77 (538.00, 717.06) | 1236501 (1123821, 1360228) | 728.21 (660.25, 801.45) | 0.48 (0.45, 0.52) | 0.65 (-2.15, 3.44) | 21028 (18056, 23925) | 32.01 (27.46, 36.41) | 71106 (64331, 78126) | 43.23 (38.97, 47.53) | 0.99 (0.96, 1.02) | 1.76 (1.74, 1.77) | 58828 (51133, 66791) | 84.53 (73.41, 96.22) | 235070 (213788, 258177) | 136.20 (123.81, 149.54) | 1.59 (1.54, 1.64) | 2.39 (2.30, 2.48) |
| 8 | Low SDI | 9759 (7718, 11201) | 42.36 (33.80, 48.60) | 20654 (18280, 23210) | 42.20 (37.16, 47.41) | -0.04 (-0.14, 0.06) | 0.02 (-0.01, 0.05) | 213295 (167669, 244926) | 834.06 (660.17, 956.94) | 421925 (374004, 476166) | 775.74 (686.11, 874.33) | -0.30 (-0.39, -0.21) | -0.40 (-0.85, 0.05) | 9720 (7668, 11154) | 40.48 (32.18, 46.41) | 21697 (19204, 24406) | 42.20 (37.18, 47.44) | 0.09 (-0.02, 0.20) | 0.24 (0.22, 0.27) | 23841 (19260, 27366) | 92.49 (75.15, 106.23) | 59220 (52836, 66418) | 105.98 (94.60, 118.57) | 0.40 (0.27, 0.52) | 0.72 (0.66, 0.77) |
| 9 | Andean Latin America | 1260 (1044, 1489) | 56.53 (46.86, 66.82) | 4528 (3506, 5722) | 64.42 (49.89, 81.35) | 0.53 (0.43, 0.63) | 0.81 (0.69, 0.92) | 23756 (19651, 28071) | 1023.87 (847.36, 1209.79) | 83005 (64125, 105328) | 1163.50 (899.14, 1475.44) | 0.49 (0.40, 0.59) | 0.77 (-1.55, 3.08) | 1294 (1073, 1537) | 57.02 (47.27, 67.71) | 6084 (4677, 7724) | 85.75 (65.93, 108.79) | 1.43 (1.34, 1.52) | 2.61 (2.52, 2.70) | 4420 (3694, 5246) | 189.38 (158.37, 224.82) | 29556 (22721, 37671) | 413.40 (317.85, 526.56) | 2.79 (2.67, 2.91) | 5.09 (4.61, 5.56) |
| 10 | Australasia | 4763 (4207, 5285) | 158.29 (139.02, 176.04) | 7256 (6068, 8383) | 95.38 (80.09, 109.98) | -1.85 (-1.93, -1.76) | -3.09 (-3.23, -2.95) | 92210 (82518, 101957) | 2991.34 (2669.34, 3311.03) | 123716 (105187, 142563) | 1699.06 (1448.94, 1955.50) | -2.10 (-2.20, -2.00) | -3.57 (-6.68, -0.47) | 9134 (8064, 10145) | 296.85 (261.29, 330.24) | 18709 (15561, 21851) | 254.81 (212.43, 297.29) | -0.65 (-0.84, -0.46) | -0.54 (-1.63, 0.55) | 46316 (41173, 51688) | 1488.24 (1320.75, 1664.13) | 109687 (92893, 126616) | 1518.62 (1286.85, 1752.40) | -0.02 (-0.28, 0.25) | 0.05 (-3.10, 3.20) |
| 11 | Caribbean | 2702 (2452, 2958) | 89.16 (80.49, 97.83) | 6356 (5417, 7300) | 93.75 (80.00, 107.65) | 0.28 (0.24, 0.32) | 0.40 (0.36, 0.44) | 50779 (46239, 55589) | 1607.89 (1460.30, 1762.50) | 116397 (99540, 133726) | 1729.74 (1480.06, 1987.00) | 0.36 (0.33, 0.40) | 0.54 (-0.09, 1.17) | 4482 (4044, 4949) | 142.59 (128.28, 157.66) | 13818 (11614, 16022) | 205.46 (172.75, 238.20) | 1.30 (1.23, 1.38) | 2.01 (1.86, 2.16) | 16855 (15110, 18714) | 522.50 (467.87, 580.85) | 63606 (53287, 73771) | 948.70 (794.71, 1100.31) | 2.16 (2.03, 2.29) | 3.34 (2.46, 4.22) |
| 12 | Central Asia | 3138 (2873, 3391) | 57.84 (52.86, 62.53) | 4269 (3794, 4722) | 48.04 (42.65, 53.08) | -0.09 (-0.27, 0.09) | -0.32 (-1.24, 0.60) | 67679 (62235, 73147) | 1195.46 (1098.05, 1291.97) | 91207 (81225, 101000) | 950.98 (846.79, 1051.45) | -0.30 (-0.45, -0.15) | -0.56 (-15.24, 14.11) | 3667 (3356, 3972) | 66.27 (60.59, 71.79) | 5635 (4994, 6236) | 60.83 (53.90, 67.25) | 0.27 (0.05, 0.49) | 0.10 (-0.40, 0.61) | 12167 (11102, 13323) | 214.35 (195.54, 235.06) | 21230 (18961, 23517) | 217.66 (194.66, 240.64) | 0.55 (0.29, 0.81) | 0.54 (-0.40, 1.47) |
| 13 | Central Europe | 25553 (24114, 26809) | 139.42 (130.84, 146.62) | 45827 (41571, 49474) | 149.14 (135.30, 161.03) | 0.09 (-0.02, 0.21) | 0.25 (0.02, 0.47) | 503740 (477534, 528272) | 2613.15 (2469.89, 2743.46) | 848536 (775595, 915129) | 2794.03 (2554.18, 3013.34) | 0.10 (-0.01, 0.21) | 0.25 (-4.00, 4.50) | 31199 (29382, 32839) | 164.91 (154.74, 173.81) | 70918 (64442, 77200) | 232.42 (211.18, 253.02) | 1.05 (0.90, 1.20) | 1.52 (1.27, 1.76) | 106844 (99498, 115124) | 547.82 (508.50, 592.78) | 302824 (275482, 330022) | 999.83 (909.57, 1089.65) | 2.06 (1.89, 2.24) | 3.02 (2.44, 3.59) |
| 14 | Central Latin America | 3954 (3693, 4178) | 45.41 (42.14, 48.07) | 16550 (14468, 18602) | 55.45 (48.45, 62.31) | 0.68 (0.60, 0.77) | 1.06 (1.01, 1.11) | 74930 (70551, 79043) | 808.24 (758.15, 853.67) | 321628 (283289, 361737) | 1050.95 (925.24, 1181.72) | 0.86 (0.79, 0.94) | 1.39 (0.59, 2.19) | 5007 (4683, 5283) | 55.29 (51.50, 58.44) | 30061 (26310, 33871) | 99.02 (86.62, 111.54) | 1.86 (1.81, 1.92) | 3.10 (3.04, 3.15) | 16143 (15066, 17215) | 170.88 (159.14, 182.63) | 126893 (111321, 142865) | 412.44 (361.69, 464.34) | 2.92 (2.86, 2.98) | 4.59 (4.33, 4.85) |
| 15 | Central Sub-Saharan Africa | 939 (718, 1211) | 45.17 (34.41, 59.16) | 2229 (1629, 3076) | 45.83 (33.15, 65.21) | 0.09 (-0.05, 0.24) | 0.11 (0.07, 0.14) | 20920 (16002, 26940) | 865.19 (661.17, 1121.84) | 48263 (35376, 65864) | 866.93 (632.94, 1203.25) | 0.05 (-0.09, 0.19) | 0.05 (-0.60, 0.69) | 939 (719, 1214) | 42.60 (32.51, 55.63) | 2341 (1720, 3180) | 45.46 (33.15, 63.31) | 0.25 (0.08, 0.42) | 0.34 (0.31, 0.37) | 2340 (1819, 2997) | 95.60 (74.59, 122.23) | 6482 (4862, 8643) | 113.63 (85.62, 152.57) | 0.61 (0.39, 0.82) | 0.89 (0.82, 0.95) |
| 16 | East Asia | 75740 (64848, 86768) | 88.11 (75.37, 100.83) | 218412 (177231, 260423) | 84.30 (68.34, 100.32) | -0.19 (-0.27, -0.12) | -0.21 (-0.39, -0.04) | 1588971 (1357443, 1824081) | 1613.87 (1379.40, 1849.84) | 4219903 (3426848, 5054020) | 1542.72 (1252.44, 1844.84) | -0.17 (-0.24, -0.11) | -0.21 (-3.39, 2.96) | 92021 (78919, 105215) | 98.99 (84.85, 113.06) | 466938 (378119, 558881) | 171.44 (138.76, 204.89) | 1.92 (1.85, 2.00) | 2.28 (1.70, 2.86) | 317823 (273989, 365568) | 310.94 (267.34, 359.23) | 2320067 (1899014, 2769249) | 821.31 (672.58, 979.08) | 3.53 (3.40, 3.66) | 3.98 (1.59, 6.37) |
| 17 | Eastern Europe | 38033 (35933, 39961) | 108.06 (101.74, 113.69) | 54399 (49146, 59371) | 115.54 (104.38, 126.07) | 0.08 (-0.02, 0.18) | 0.21 (-0.11, 0.54) | 795601 (753948, 836193) | 2169.43 (2052.19, 2280.93) | 1066622 (970079, 1165723) | 2229.42 (2027.82, 2435.80) | -0.10 (-0.22, 0.02) | 0.00 (-8.47, 8.47) | 50955 (48058, 53781) | 141.40 (133.06, 149.32) | 90072 (81979, 98078) | 189.74 (172.68, 206.56) | 0.88 (0.78, 0.98) | 1.38 (1.02, 1.75) | 191638 (178484, 206948) | 521.95 (484.56, 565.95) | 381310 (349988, 413925) | 794.59 (729.42, 862.06) | 1.39 (1.22, 1.56) | 1.93 (0.04, 3.83) |
| 18 | Eastern Sub-Saharan Africa | 5014 (3901, 5827) | 66.14 (52.04, 76.80) | 10591 (9083, 12266) | 68.57 (58.56, 79.36) | 0.03 (-0.05, 0.11) | 0.16 (0.14, 0.19) | 109511 (84504, 127463) | 1302.81 (1014.17, 1514.51) | 212586 (182426, 247031) | 1227.78 (1051.61, 1424.30) | -0.31 (-0.40, -0.23) | -0.41 (-0.97, 0.15) | 4954 (3851, 5752) | 62.76 (49.22, 72.79) | 10916 (9361, 12636) | 67.22 (57.46, 77.74) | 0.12 (0.01, 0.22) | 0.34 (0.31, 0.36) | 11709 (9362, 13451) | 138.16 (111.22, 158.85) | 28858 (24996, 33377) | 161.64 (140.13, 186.42) | 0.42 (0.29, 0.54) | 0.79 (0.68, 0.89) |
| 19 | High-income Asia Pacific | 27233 (25022, 28700) | 115.39 (104.90, 122.12) | 74039 (60100, 82418) | 99.35 (83.46, 109.20) | -0.54 (-0.59, -0.49) | -0.37 (-4.29, 3.54) | 525669 (490566, 552060) | 2118.77 (1965.02, 2230.52) | 1166435 (992391, 1280873) | 1785.63 (1559.22, 1942.69) | -0.63 (-0.69, -0.57) | -0.41 (-75.97, 75.14) | 56153 (51461, 59697) | 228.60 (208.16, 243.58) | 178201 (148614, 200209) | 269.34 (229.50, 299.93) | 0.46 (0.36, 0.56) | 0.51 (0.24, 0.77) | 307472 (283819, 331403) | 1224.58 (1126.13, 1324.87) | 1016533 (874304, 1129241) | 1623.65 (1413.23, 1792.26) | 0.86 (0.76, 0.97) | 1.19 (-0.03, 2.41) |
| 20 | High-income North America | 63481 (57364, 67111) | 133.97 (120.96, 141.69) | 71233 (62163, 76601) | 78.37 (68.72, 84.12) | -1.93 (-2.01, -1.84) | -3.39 (-3.49, -3.30) | 1177137 (1090031, 1240072) | 2507.41 (2322.88, 2640.94) | 1300938 (1177104, 1388300) | 1458.38 (1323.30, 1554.51) | -1.95 (-2.03, -1.87) | -3.37 (-5.30, -1.45) | 135963 (124103, 143494) | 286.91 (261.93, 302.81) | 185876 (165679, 198930) | 207.88 (185.70, 222.28) | -1.28 (-1.44, -1.12) | -1.36 (-2.04, -0.68) | 729157 (674118, 780704) | 1542.33 (1426.98, 1651.52) | 1090409 (994681, 1159680) | 1227.39 (1120.44, 1304.81) | -0.91 (-1.07, -0.75) | -0.99 (-3.48, 1.50) |
| 21 | North Africa and Middle East | 8906 (7525, 10369) | 53.15 (44.73, 62.16) | 25811 (21923, 29951) | 56.19 (47.52, 65.33) | 0.44 (0.29, 0.59) | 0.54 (0.37, 0.71) | 183806 (155390, 213152) | 985.03 (831.90, 1145.01) | 515104 (438897, 597733) | 1029.83 (875.40, 1196.08) | 0.34 (0.21, 0.47) | 0.43 (-2.70, 3.56) | 9806 (8294, 11383) | 55.59 (46.91, 64.78) | 38902 (33088, 45235) | 79.95 (67.74, 93.10) | 1.46 (1.30, 1.62) | 1.93 (1.72, 2.14) | 35346 (30401, 40637) | 187.85 (161.34, 216.67) | 194543 (166688, 226033) | 383.11 (327.89, 445.17) | 2.57 (2.44, 2.70) | 3.44 (2.51, 4.36) |
| 22 | Oceania | 94 (78, 113) | 37.08 (30.83, 44.05) | 220 (183, 260) | 33.14 (27.62, 38.99) | -0.33 (-0.44, -0.22) | -0.48 (-0.51, -0.45) | 2062 (1692, 2486) | 681.84 (563.97, 815.05) | 4656 (3875, 5530) | 613.43 (511.25, 725.29) | -0.31 (-0.41, -0.22) | -0.46 (-0.99, 0.07) | 101 (84, 121) | 36.70 (30.48, 43.55) | 250 (210, 295) | 35.13 (29.46, 41.35) | -0.12 (-0.22, -0.03) | -0.14 (-0.18, -0.11) | 295 (248, 348) | 94.77 (79.74, 111.44) | 782 (667, 911) | 99.55 (85.26, 115.39) | 0.15 (0.05, 0.25) | 0.29 (0.20, 0.39) |
| 23 | South Asia | 14217 (11872, 16455) | 24.00 (19.92, 27.91) | 42966 (38070, 48484) | 25.92 (22.91, 29.27) | 0.11 (0.01, 0.22) | 0.29 (0.26, 0.33) | 315841 (264707, 364309) | 483.51 (403.98, 559.27) | 904734 (803261, 1022443) | 508.33 (450.88, 574.54) | 0.00 (-0.10, 0.11) | 0.17 (-0.43, 0.76) | 14593 (12222, 16846) | 23.65 (19.71, 27.41) | 49912 (44196, 56247) | 28.98 (25.62, 32.67) | 0.50 (0.38, 0.62) | 1.08 (1.05, 1.11) | 39115 (33417, 45084) | 59.38 (50.63, 68.73) | 156578 (139857, 176005) | 86.42 (77.18, 97.11) | 1.05 (0.91, 1.19) | 2.23 (2.17, 2.29) |
| 24 | Southeast Asia | 15245 (12840, 17552) | 58.35 (49.11, 67.23) | 54456 (46506, 62502) | 76.63 (65.24, 88.10) | 0.87 (0.79, 0.94) | 1.30 (1.26, 1.34) | 321343 (269918, 370240) | 1123.45 (944.46, 1293.89) | 1123355 (961179, 1290235) | 1452.01 (1241.30, 1668.05) | 0.82 (0.75, 0.89) | 1.23 (0.54, 1.92) | 16255 (13725, 18722) | 59.53 (50.22, 68.61) | 72172 (61244, 83245) | 96.37 (81.58, 111.22) | 1.55 (1.49, 1.61) | 2.41 (2.35, 2.47) | 48218 (41300, 55386) | 166.26 (142.33, 191.48) | 270597 (230623, 311706) | 340.79 (290.59, 392.20) | 2.38 (2.34, 2.42) | 3.10 (2.78, 3.43) |
| 25 | Southern Latin America | 7292 (6402, 8213) | 131.25 (114.78, 148.15) | 13462 (11380, 15578) | 117.07 (99.03, 135.48) | -0.14 (-0.31, 0.03) | -0.05 (-0.44, 0.33) | 137584 (120938, 155059) | 2369.43 (2078.69, 2672.60) | 240394 (205088, 278052) | 2118.67 (1808.01, 2451.21) | -0.15 (-0.30, 0.01) | -0.11 (-5.59, 5.38) | 8407 (7367, 9504) | 147.41 (128.78, 166.81) | 19097 (16135, 22137) | 167.31 (141.41, 193.98) | 0.60 (0.42, 0.77) | 0.96 (0.45, 1.46) | 27238 (23846, 30837) | 464.20 (405.81, 526.67) | 75764 (64843, 87315) | 669.25 (572.60, 771.66) | 1.44 (1.28, 1.60) | 2.02 (1.42, 2.62) |
| 26 | Southern Sub-Saharan Africa | 1486 (1258, 1892) | 53.24 (44.93, 67.87) | 4171 (3680, 4675) | 69.84 (61.41, 78.30) | 0.94 (0.67, 1.21) | 1.40 (1.20, 1.60) | 28067 (23829, 35775) | 930.13 (787.97, 1185.75) | 83110 (73352, 93273) | 1266.49 (1115.93, 1420.84) | 1.11 (0.82, 1.40) | 1.57 (-1.59, 4.72) | 1518 (1289, 1930) | 52.55 (44.44, 66.84) | 4731 (4158, 5310) | 75.56 (66.25, 84.78) | 1.25 (1.01, 1.48) | 1.81 (1.62, 2.00) | 4130 (3526, 5227) | 135.09 (115.06, 170.92) | 13814 (12219, 15514) | 204.68 (181.12, 229.45) | 1.48 (1.38, 1.59) | 2.38 (2.24, 2.52) |
| 27 | Tropical Latin America | 5632 (5168, 6035) | 59.07 (53.59, 63.55) | 21459 (18950, 23439) | 68.64 (60.43, 75.03) | 0.57 (0.48, 0.65) | 0.85 (0.74, 0.96) | 111271 (103190, 118837) | 1073.46 (988.44, 1149.09) | 414424 (372720, 449378) | 1294.44 (1161.42, 1404.67) | 0.65 (0.56, 0.74) | 0.97 (-1.06, 3.00) | 6327 (5824, 6781) | 63.49 (57.91, 68.23) | 29485 (26248, 32176) | 92.98 (82.55, 101.55) | 1.26 (1.15, 1.37) | 1.79 (1.63, 1.94) | 19404 (17987, 20889) | 182.95 (168.84, 197.52) | 115247 (103799, 125757) | 357.92 (321.83, 390.73) | 2.22 (2.11, 2.33) | 2.96 (2.18, 3.74) |
| 28 | Western Europe | 118976 (109442, 126006) | 152.65 (139.94, 161.85) | 142103 (121431, 155416) | 101.74 (88.43, 110.60) | -1.37 (-1.41, -1.32) | -1.36 (-2.34, -0.37) | 2112179 (1969790, 2224645) | 2729.33 (2544.06, 2875.08) | 2321433 (2045063, 2518905) | 1821.07 (1626.92, 1965.88) | -1.37 (-1.43, -1.32) | -1.37 (-15.86, 13.13) | 199335 (183794, 211095) | 254.93 (234.96, 270.05) | 315496 (276453, 343169) | 244.92 (217.51, 265.14) | -0.16 (-0.30, -0.02) | -0.25 (-0.70, 0.19) | 922488 (855761, 988727) | 1186.34 (1101.22, 1272.37) | 1736012 (1563806, 1871317) | 1405.37 (1275.10, 1510.28) | 0.62 (0.42, 0.81) | 0.85 (-0.32, 2.03) |
| 29 | Western Sub-Saharan Africa | 2968 (2522, 3466) | 33.09 (28.10, 38.49) | 7134 (6015, 8259) | 38.99 (33.13, 44.87) | 0.72 (0.65, 0.79) | 0.88 (0.70, 1.07) | 60229 (51129, 70666) | 612.81 (520.42, 716.27) | 141260 (117810, 164859) | 697.76 (586.33, 810.01) | 0.60 (0.53, 0.66) | 0.74 (-2.47, 3.95) | 2912 (2470, 3401) | 31.28 (26.51, 36.39) | 7399 (6208, 8605) | 38.66 (32.68, 44.70) | 0.87 (0.79, 0.94) | 1.05 (0.90, 1.21) | 7039 (6012, 8222) | 70.78 (60.50, 82.59) | 19180 (16091, 22381) | 92.40 (78.14, 107.17) | 1.04 (0.93, 1.15) | 1.30 (1.13, 1.48) |
| Liver cancer |  |  |  |  |  |  |  |  |  |  |  |  |  |  |  |  |  |  |  |  |  |  |  |  |  |
| 1 | Global | 135915 (123735, 150355) | 28.68 (26.01, 31.77) | 332455 (296105, 368772) | 31.12 (27.64, 34.52) | 0.18 (0.06, 0.31) | 0.47 (0.34, 0.59) | 2925891 (2672345, 3229636) | 590.70 (538.50, 652.61) | 6538211 (5884148, 7257231) | 599.59 (538.71, 665.49) | -0.06 (-0.19, 0.07) | 0.21 (-2.95, 3.36) | 130055 (118733, 143308) | 27.05 (24.62, 29.85) | 341395 (304410, 378506) | 31.78 (28.26, 35.23) | 0.40 (0.27, 0.54) | 0.74 (0.62, 0.87) | 136181 (125309, 149340) | 27.74 (25.46, 30.44) | 417259 (372090, 463352) | 38.63 (34.35, 42.92) | 0.98 (0.78, 1.19) | 1.48 (1.36, 1.60) |
| 2 | Male | 84699 (76950, 94153) | 40.03 (36.34, 44.51) | 209239 (185475, 240873) | 43.43 (38.42, 49.88) | 0.12 (-0.02, 0.27) | 0.43 (0.25, 0.61) | 1894039 (1720100, 2106174) | 836.53 (759.59, 930.38) | 4268658 (3788436, 4935063) | 845.10 (749.39, 975.51) | -0.13 (-0.27, 0.02) | 0.16 (-4.82, 5.14) | 82281 (74882, 91144) | 38.09 (34.64, 42.19) | 220031 (195027, 252177) | 45.06 (39.87, 51.54) | 0.38 (0.23, 0.52) | 0.73 (0.56, 0.90) | 88164 (80677, 97086) | 39.48 (36.11, 43.45) | 279816 (248149, 319004) | 56.64 (50.10, 64.53) | 1.06 (0.86, 1.25) | 1.57 (1.39, 1.76) |
| 3 | Female | 51216 (44653, 59270) | 19.57 (17.00, 22.67) | 123216 (106227, 138065) | 20.93 (18.06, 23.45) | 0.24 (0.14, 0.33) | 0.48 (0.39, 0.56) | 1031852 (903671, 1193314) | 384.11 (335.79, 444.41) | 2269553 (1997669, 2533768) | 386.46 (340.31, 431.40) | 0.02 (-0.07, 0.10) | 0.22 (-1.52, 1.95) | 47774 (41825, 55006) | 18.09 (15.79, 20.84) | 121364 (104427, 136376) | 20.64 (17.77, 23.19) | 0.39 (0.28, 0.50) | 0.40 (0.38, 0.42) | 48018 (42380, 54651) | 17.96 (15.82, 20.45) | 137443 (117076, 156165) | 23.41 (19.94, 26.59) | 0.80 (0.59, 1.02) | 1.07 (1.03, 1.11) |
| 4 | High SDI | 39524 (36379, 42258) | 27.30 (25.11, 29.21) | 100963 (88823, 109085) | 34.71 (30.84, 37.38) | 0.42 (0.11, 0.74) | 0.99 (0.85, 1.13) | 814839 (756299, 870741) | 566.22 (525.43, 605.15) | 1791858 (1618803, 1921598) | 647.67 (589.17, 693.05) | 0.00 (-0.31, 0.32) | 0.56 (-2.43, 3.56) | 41188 (37957, 43935) | 28.48 (26.23, 30.39) | 116679 (102968, 126465) | 40.81 (36.32, 44.10) | 0.68 (0.35, 1.01) | 1.21 (1.04, 1.38) | 50699 (46896, 54295) | 35.14 (32.49, 37.64) | 179499 (156944, 198245) | 63.79 (56.21, 70.21) | 1.52 (1.06, 1.98) | 2.04 (1.90, 2.18) |
| 5 | High-middle SDI | 33833 (30071, 38062) | 27.68 (24.55, 31.13) | 72801 (61872, 84773) | 28.66 (24.34, 33.35) | 0.09 (-0.04, 0.22) | 0.34 (0.17, 0.51) | 732170 (650828, 825796) | 570.75 (506.94, 643.48) | 1446523 (1232194, 1691008) | 561.56 (478.26, 656.22) | -0.09 (-0.22, 0.05) | 0.22 (-5.88, 6.32) | 31490 (27973, 35370) | 25.34 (22.47, 28.46) | 72670 (61519, 85135) | 28.45 (24.08, 33.31) | 0.38 (0.24, 0.52) | 0.74 (0.54, 0.93) | 30893 (27538, 34654) | 24.24 (21.58, 27.18) | 81569 (68829, 96361) | 31.72 (26.77, 37.46) | 0.93 (0.78, 1.08) | 1.18 (1.14, 1.22) |
| 6 | Middle SDI | 39343 (34304, 45053) | 34.43 (29.92, 39.48) | 103713 (88892, 122129) | 32.61 (27.87, 38.40) | -0.02 (-0.14, 0.10) | 0.01 (-0.09, 0.10) | 868328 (758067, 995435) | 703.17 (613.02, 806.07) | 2123483 (1824464, 2502605) | 637.62 (546.99, 751.40) | -0.18 (-0.30, -0.06) | -0.24 (-2.10, 1.61) | 36243 (31538, 41545) | 30.91 (26.83, 35.46) | 102101 (87230, 120847) | 31.59 (26.93, 37.39) | 0.25 (0.13, 0.37) | 0.40 (0.31, 0.49) | 34883 (30358, 40061) | 28.56 (24.82, 32.80) | 109037 (92427, 130018) | 32.97 (27.91, 39.30) | 0.68 (0.53, 0.83) | 1.07 (0.97, 1.16) |
| 7 | Low-middle SDI | 14196 (11718, 18116) | 21.33 (17.49, 27.44) | 38641 (34148, 43806) | 23.31 (20.58, 26.38) | 0.26 (0.20, 0.32) | 0.34 (0.32, 0.37) | 312857 (259450, 396433) | 437.91 (361.65, 558.05) | 829421 (732769, 943237) | 475.05 (419.74, 539.54) | 0.23 (0.17, 0.28) | 0.34 (-0.09, 0.76) | 12937 (10676, 16481) | 19.00 (15.59, 24.39) | 35204 (31118, 39958) | 20.88 (18.44, 23.66) | 0.32 (0.26, 0.37) | 0.38 (0.37, 0.39) | 12134 (10043, 15390) | 17.19 (14.16, 21.93) | 33452 (29558, 38069) | 19.32 (17.07, 21.95) | 0.39 (0.34, 0.45) | 0.49 (0.48, 0.50) |
| 8 | Low SDI | 8911 (6529, 12389) | 37.49 (27.31, 52.66) | 16134 (13178, 20030) | 30.78 (25.14, 38.10) | -0.86 (-0.95, -0.78) | -0.77 (-1.00, -0.53) | 195451 (143483, 270081) | 751.58 (549.96, 1045.35) | 342879 (279713, 428165) | 603.74 (492.75, 751.79) | -0.94 (-1.02, -0.85) | -0.85 (-5.47, 3.77) | 8099 (5928, 11221) | 33.10 (24.10, 46.28) | 14551 (11889, 18019) | 27.03 (22.09, 33.39) | -0.88 (-0.97, -0.80) | -0.79 (-0.98, -0.61) | 7478 (5480, 10331) | 29.19 (21.31, 40.62) | 13503 (11036, 16755) | 24.07 (19.68, 29.79) | -0.85 (-0.92, -0.77) | -0.75 (-0.91, -0.60) |
| 9 | Andean Latin America | 361 (293, 447) | 15.71 (12.75, 19.52) | 1200 (885, 1593) | 16.94 (12.49, 22.46) | 0.08 (-0.19, 0.35) | -0.00 (-0.16, 0.15) | 7247 (5897, 8957) | 306.07 (248.95, 378.77) | 22985 (16938, 30583) | 320.22 (236.01, 425.89) | 0.01 (-0.26, 0.27) | -0.11 (-2.93, 2.72) | 320 (260, 396) | 13.80 (11.21, 17.12) | 1064 (787, 1414) | 14.96 (11.07, 19.86) | 0.15 (-0.10, 0.39) | 0.05 (-0.03, 0.14) | 294 (239, 364) | 12.49 (10.16, 15.47) | 987 (730, 1315) | 13.78 (10.19, 18.36) | 0.23 (-0.02, 0.47) | 0.17 (0.09, 0.24) |
| 10 | Australasia | 325 (277, 375) | 10.47 (8.91, 12.09) | 2104 (1736, 2475) | 28.99 (24.00, 34.06) | 3.13 (2.96, 3.30) | 5.17 (5.14, 5.21) | 6671 (5705, 7700) | 213.49 (182.33, 246.47) | 39381 (32837, 46150) | 563.01 (470.51, 659.35) | 2.96 (2.81, 3.11) | 4.93 (4.34, 5.53) | 315 (269, 364) | 10.11 (8.61, 11.68) | 2228 (1842, 2629) | 31.17 (25.84, 36.74) | 3.52 (3.34, 3.71) | 5.15 (5.11, 5.19) | 333 (286, 388) | 10.66 (9.14, 12.43) | 2949 (2408, 3567) | 42.12 (34.42, 50.97) | 4.58 (4.29, 4.86) | 7.31 (7.28, 7.34) |
| 11 | Caribbean | 392 (338, 458) | 12.45 (10.74, 14.58) | 741 (617, 876) | 11.01 (9.16, 13.00) | -0.75 (-1.06, -0.45) | -1.11 (-1.15, -1.06) | 7647 (6605, 8967) | 237.50 (205.04, 278.51) | 14352 (11945, 17011) | 213.98 (178.10, 253.67) | -0.67 (-0.97, -0.37) | -0.97 (-1.79, -0.15) | 349 (301, 408) | 10.98 (9.46, 12.86) | 666 (556, 786) | 9.91 (8.27, 11.70) | -0.71 (-1.00, -0.42) | -0.97 (-0.99, -0.95) | 321 (277, 376) | 9.99 (8.62, 11.68) | 634 (528, 747) | 9.46 (7.88, 11.13) | -0.54 (-0.83, -0.25) | -0.68 (-0.70, -0.65) |
| 12 | Central Asia | 2299 (2030, 2600) | 41.62 (36.64, 47.31) | 3828 (3227, 4524) | 41.70 (35.08, 49.32) | -0.08 (-0.19, 0.04) | -0.26 (-0.33, -0.19) | 50985 (45212, 57235) | 887.08 (784.55, 1000.20) | 83864 (70888, 99039) | 845.51 (713.40, 999.09) | -0.26 (-0.36, -0.16) | -0.56 (-1.83, 0.71) | 2098 (1856, 2366) | 37.56 (33.13, 42.57) | 3496 (2954, 4121) | 37.28 (31.43, 44.00) | -0.02 (-0.14, 0.09) | -0.21 (-0.25, -0.16) | 1989 (1765, 2234) | 34.91 (30.89, 39.37) | 3336 (2823, 3929) | 34.21 (28.90, 40.31) | -0.05 (-0.15, 0.05) | -0.23 (-0.28, -0.19) |
| 13 | Central Europe | 3507 (3183, 3865) | 18.55 (16.80, 20.48) | 5637 (5001, 6325) | 18.51 (16.43, 20.78) | -0.05 (-0.22, 0.12) | 0.22 (0.19, 0.25) | 72960 (66404, 80353) | 370.55 (336.77, 408.38) | 111185 (99080, 124910) | 370.40 (330.19, 416.29) | -0.04 (-0.21, 0.13) | 0.23 (-0.31, 0.78) | 3168 (2877, 3491) | 16.53 (14.98, 18.24) | 5139 (4560, 5760) | 16.93 (15.03, 18.98) | 0.02 (-0.11, 0.15) | 0.03 (0.00, 0.07) | 2963 (2694, 3263) | 15.13 (13.73, 16.67) | 4995 (4444, 5602) | 16.57 (14.75, 18.59) | 0.28 (0.16, 0.40) | 0.41 (0.38, 0.44) |
| 14 | Central Latin America | 1762 (1653, 1862) | 19.24 (17.98, 20.36) | 5871 (5239, 6486) | 19.59 (17.47, 21.63) | 0.12 (-0.18, 0.41) | -0.10 (-0.18, -0.01) | 35496 (33464, 37420) | 370.95 (348.98, 391.43) | 113853 (101959, 125910) | 371.11 (332.25, 410.29) | 0.05 (-0.26, 0.36) | -0.20 (-1.90, 1.49) | 1569 (1472, 1657) | 16.89 (15.79, 17.86) | 5226 (4665, 5778) | 17.32 (15.45, 19.14) | 0.23 (-0.04, 0.50) | 0.02 (-0.01, 0.05) | 1449 (1364, 1529) | 15.26 (14.32, 16.11) | 4878 (4362, 5395) | 15.98 (14.29, 17.67) | 0.31 (0.05, 0.58) | 0.14 (0.11, 0.16) |
| 15 | Central Sub-Saharan Africa | 971 (442, 2039) | 42.44 (18.77, 91.97) | 1599 (728, 3543) | 31.05 (13.80, 70.18) | -1.34 (-1.46, -1.21) | -2.11 (-2.15, -2.06) | 22237 (10181, 46330) | 864.12 (389.66, 1834.07) | 35446 (16333, 77838) | 611.37 (277.33, 1358.55) | -1.43 (-1.54, -1.31) | -2.25 (-3.24, -1.26) | 892 (407, 1870) | 37.55 (16.71, 80.81) | 1453 (667, 3171) | 27.19 (12.23, 60.41) | -1.36 (-1.49, -1.24) | -2.15 (-2.19, -2.11) | 833 (382, 1735) | 33.03 (14.87, 70.19) | 1371 (633, 2980) | 24.15 (10.97, 53.25) | -1.31 (-1.43, -1.19) | -2.06 (-2.10, -2.03) |
| 16 | East Asia | 45571 (38477, 53499) | 46.95 (39.55, 55.16) | 110050 (89239, 134947) | 40.92 (33.18, 50.14) | -0.22 (-0.38, -0.06) | -0.92 (-1.00, -0.83) | 1018287 (858835, 1197331) | 953.91 (803.90, 1121.48) | 2222446 (1795389, 2736668) | 794.51 (641.89, 977.89) | -0.42 (-0.58, -0.26) | -1.30 (-2.90, 0.31) | 42407 (35787, 49833) | 42.28 (35.60, 49.70) | 113540 (91752, 139365) | 41.57 (33.59, 50.99) | 0.19 (0.05, 0.33) | -0.17 (-0.23, -0.11) | 41350 (34868, 48671) | 39.18 (32.99, 46.10) | 129851 (104215, 159807) | 46.62 (37.43, 57.34) | 0.84 (0.70, 0.98) | 1.00 (0.93, 1.07) |
| 17 | Eastern Europe | 3403 (3159, 3646) | 9.47 (8.77, 10.15) | 6356 (5781, 6914) | 13.41 (12.19, 14.58) | 1.26 (1.02, 1.50) | 1.69 (1.65, 1.72) | 73873 (68880, 78920) | 197.70 (184.06, 211.35) | 128806 (117709, 140228) | 267.11 (244.11, 290.65) | 1.10 (0.85, 1.35) | 1.37 (0.62, 2.11) | 3121 (2898, 3347) | 8.58 (7.95, 9.21) | 5782 (5260, 6279) | 12.15 (11.05, 13.18) | 1.22 (1.02, 1.43) | 1.65 (1.62, 1.67) | 2982 (2772, 3195) | 8.04 (7.46, 8.62) | 5544 (5051, 6020) | 11.55 (10.53, 12.54) | 1.31 (1.10, 1.51) | 1.78 (1.76, 1.81) |
| 18 | Eastern Sub-Saharan Africa | 2817 (2150, 3744) | 36.20 (27.47, 48.36) | 5595 (4264, 7455) | 33.12 (25.26, 44.03) | -0.62 (-0.79, -0.45) | -0.47 (-0.58, -0.37) | 61750 (47201, 81880) | 723.56 (551.51, 962.24) | 119205 (90559, 159376) | 648.52 (493.45, 865.01) | -0.69 (-0.86, -0.51) | -0.54 (-2.50, 1.43) | 2560 (1953, 3388) | 31.92 (24.22, 42.43) | 5047 (3845, 6717) | 29.07 (22.16, 38.61) | -0.65 (-0.82, -0.48) | -0.51 (-0.60, -0.42) | 2349 (1792, 3106) | 27.96 (21.25, 37.10) | 4666 (3557, 6206) | 25.74 (19.64, 34.15) | -0.60 (-0.76, -0.43) | -0.46 (-0.53, -0.39) |
| 19 | High-income Asia Pacific | 20394 (18171, 22640) | 81.04 (71.93, 90.03) | 40320 (33549, 45700) | 58.40 (49.34, 66.26) | -1.73 (-2.20, -1.25) | -1.02 (-1.80, -0.23) | 438343 (393503, 486264) | 1698.15 (1521.34, 1884.58) | 647089 (548416, 734939) | 1051.17 (898.56, 1198.16) | -2.32 (-2.79, -1.83) | -1.58 (-19.10, 15.93) | 22495 (20158, 24790) | 88.62 (79.14, 97.72) | 50164 (41597, 57573) | 75.50 (63.40, 86.73) | -1.37 (-1.81, -0.93) | -0.83 (-1.59, -0.06) | 30761 (27619, 33872) | 119.89 (107.36, 132.15) | 89339 (71602, 105949) | 139.20 (113.20, 164.99) | -0.26 (-0.78, 0.26) | -0.05 (-0.87, 0.78) |
| 20 | High-income North America | 5494 (5091, 5763) | 11.63 (10.78, 12.20) | 23290 (21102, 24729) | 26.14 (23.73, 27.74) | 2.62 (2.53, 2.71) | 4.09 (4.06, 4.12) | 106592 (100222, 111236) | 229.07 (215.53, 238.98) | 463040 (427690, 488205) | 525.90 (486.28, 554.18) | 2.74 (2.63, 2.84) | 4.21 (3.53, 4.90) | 5818 (5403, 6101) | 12.37 (11.49, 12.97) | 27356 (24838, 29050) | 30.89 (28.08, 32.79) | 2.90 (2.81, 2.99) | 4.35 (4.32, 4.38) | 6463 (6043, 6778) | 13.85 (12.96, 14.52) | 36604 (33423, 38982) | 41.66 (38.07, 44.35) | 3.62 (3.49, 3.76) | 5.03 (4.98, 5.07) |
| 21 | North Africa and Middle East | 5301 (3799, 8005) | 29.16 (20.75, 44.50) | 15610 (12621, 19181) | 31.41 (25.48, 38.36) | 0.31 (0.17, 0.45) | 0.55 (0.50, 0.59) | 115313 (83145, 172371) | 585.20 (419.93, 881.24) | 335435 (270055, 416018) | 632.47 (510.77, 780.02) | 0.36 (0.23, 0.48) | 0.59 (-0.45, 1.63) | 4819 (3462, 7244) | 25.84 (18.45, 39.24) | 14400 (11635, 17803) | 28.38 (22.99, 34.87) | 0.42 (0.28, 0.55) | 0.62 (0.58, 0.67) | 4535 (3270, 6766) | 23.33 (16.74, 35.10) | 14059 (11349, 17437) | 26.81 (21.70, 33.05) | 0.57 (0.44, 0.71) | 0.85 (0.80, 0.89) |
| 22 | Oceania | 70 (42, 139) | 23.38 (14.33, 45.79) | 133 (86, 243) | 17.90 (11.72, 32.07) | -1.06 (-1.18, -0.95) | -1.40 (-1.45, -1.35) | 1613 (966, 3217) | 479.36 (290.52, 947.35) | 2998 (1924, 5536) | 364.81 (236.13, 664.97) | -1.07 (-1.19, -0.95) | -1.42 (-2.35, -0.49) | 65 (39, 129) | 20.83 (12.75, 41.04) | 123 (80, 222) | 16.06 (10.53, 28.50) | -1.05 (-1.17, -0.93) | -1.37 (-1.41, -1.32) | 62 (37, 124) | 18.83 (11.47, 37.27) | 120 (78, 217) | 14.85 (9.73, 26.47) | -0.97 (-1.09, -0.85) | -1.26 (-1.29, -1.22) |
| 23 | South Asia | 7322 (6259, 8413) | 11.65 (9.90, 13.43) | 27948 (24634, 31647) | 16.18 (14.24, 18.32) | 1.10 (1.00, 1.20) | 1.51 (1.48, 1.54) | 165484 (142186, 189554) | 245.69 (210.25, 282.04) | 599042 (528592, 678592) | 329.59 (290.59, 373.25) | 0.95 (0.87, 1.03) | 1.34 (0.62, 2.06) | 6718 (5746, 7711) | 10.46 (8.90, 12.05) | 25431 (22401, 28793) | 14.48 (12.73, 16.39) | 1.14 (1.06, 1.22) | 1.46 (1.44, 1.48) | 6363 (5455, 7292) | 9.56 (8.16, 10.97) | 24177 (21309, 27404) | 13.41 (11.80, 15.19) | 1.17 (1.09, 1.24) | 1.50 (1.48, 1.53) |
| 24 | Southeast Asia | 10355 (8554, 12528) | 37.19 (30.64, 45.00) | 26503 (19970, 35114) | 35.60 (26.64, 47.04) | -0.24 (-0.33, -0.15) | -0.33 (-0.38, -0.29) | 229453 (189862, 278165) | 767.48 (634.41, 929.16) | 570305 (432365, 757985) | 709.29 (535.54, 940.73) | -0.37 (-0.46, -0.29) | -0.34 (-2.08, 1.41) | 9480 (7814, 11469) | 33.29 (27.37, 40.28) | 24817 (18698, 32938) | 32.52 (24.36, 43.09) | -0.19 (-0.28, -0.10) | -0.24 (-0.27, -0.21) | 9097 (7496, 11001) | 30.79 (25.34, 37.22) | 25075 (18936, 33266) | 31.59 (23.76, 41.81) | -0.03 (-0.11, 0.06) | -0.01 (-0.04, 0.02) |
| 25 | Southern Latin America | 294 (250, 344) | 5.04 (4.28, 5.92) | 1309 (1126, 1486) | 11.49 (9.89, 13.04) | 3.32 (3.07, 3.56) | 3.85 (3.74, 3.97) | 6031 (5129, 7065) | 101.00 (85.84, 118.36) | 25667 (22210, 29042) | 228.04 (197.41, 257.91) | 3.27 (3.02, 3.52) | 3.80 (1.41, 6.19) | 265 (226, 311) | 4.51 (3.83, 5.30) | 1204 (1035, 1368) | 10.60 (9.12, 12.05) | 3.39 (3.18, 3.60) | 4.02 (3.96, 4.09) | 249 (212, 292) | 4.19 (3.56, 4.90) | 1183 (1020, 1345) | 10.48 (9.04, 11.92) | 3.61 (3.40, 3.82) | 4.32 (4.27, 4.37) |
| 26 | Southern Sub-Saharan Africa | 860 (512, 1367) | 28.97 (17.37, 45.89) | 2620 (2171, 3162) | 42.28 (35.03, 50.95) | 0.49 (-0.10, 1.08) | 1.22 (0.96, 1.49) | 17553 (10344, 28093) | 556.41 (329.51, 887.75) | 54013 (44674, 65311) | 800.44 (662.47, 966.63) | 0.45 (-0.14, 1.05) | 1.19 (-3.76, 6.14) | 766 (454, 1218) | 25.33 (15.09, 40.12) | 2346 (1944, 2833) | 36.81 (30.51, 44.37) | 0.48 (-0.07, 1.04) | 1.15 (0.98, 1.33) | 703 (415, 1122) | 22.54 (13.36, 35.85) | 2138 (1773, 2580) | 32.10 (26.64, 38.69) | 0.43 (-0.09, 0.96) | 1.09 (0.95, 1.23) |
| 27 | Tropical Latin America | 1011 (934, 1080) | 10.06 (9.23, 10.80) | 3588 (3215, 3883) | 11.35 (10.15, 12.30) | 1.01 (0.76, 1.26) | 1.10 (0.92, 1.28) | 20827 (19415, 22185) | 194.23 (180.13, 207.31) | 72029 (65460, 77620) | 223.11 (202.35, 240.57) | 1.09 (0.84, 1.33) | 1.24 (-2.53, 5.00) | 906 (837, 967) | 8.83 (8.09, 9.45) | 3219 (2893, 3481) | 10.12 (9.07, 10.96) | 1.00 (0.76, 1.24) | 1.14 (1.02, 1.26) | 841 (780, 896) | 7.92 (7.31, 8.46) | 3051 (2763, 3295) | 9.49 (8.58, 10.26) | 1.15 (0.91, 1.39) | 1.34 (1.23, 1.46) |
| 28 | Western Europe | 17013 (15683, 18162) | 21.92 (20.20, 23.41) | 37967 (33408, 41491) | 29.27 (26.07, 31.86) | 0.88 (0.76, 1.00) | 1.32 (1.28, 1.36) | 331135 (307900, 352298) | 434.23 (404.02, 461.91) | 664394 (597180, 720658) | 552.72 (501.40, 597.63) | 0.71 (0.59, 0.83) | 1.02 (0.23, 1.80) | 16155 (14890, 17247) | 20.90 (19.28, 22.31) | 39550 (34906, 43212) | 31.42 (28.04, 34.21) | 1.30 (1.17, 1.43) | 1.94 (1.91, 1.97) | 16954 (15694, 18130) | 22.10 (20.48, 23.63) | 53869 (47416, 59599) | 44.42 (39.43, 49.02) | 2.40 (2.16, 2.64) | 3.48 (3.45, 3.51) |
| 29 | Western Sub-Saharan Africa | 6395 (4194, 9569) | 66.25 (43.46, 99.41) | 10186 (8441, 12162) | 51.87 (43.17, 61.63) | -1.02 (-1.12, -0.92) | -0.91 (-1.28, -0.55) | 136397 (89432, 203587) | 1324.36 (868.57, 1980.22) | 212677 (175305, 255483) | 1001.96 (829.10, 1198.72) | -1.12 (-1.22, -1.02) | -1.01 (-9.47, 7.44) | 5769 (3780, 8615) | 58.52 (38.34, 87.62) | 9145 (7576, 10911) | 45.44 (37.79, 53.98) | -1.06 (-1.16, -0.96) | -0.96 (-1.28, -0.65) | 5292 (3468, 7892) | 51.92 (34.03, 77.61) | 8434 (6974, 10093) | 40.27 (33.42, 47.99) | -1.06 (-1.15, -0.96) | -0.96 (-1.25, -0.66) |
| Pancreatic cancer |  |  |  |  |  |  |  |  |  |  |  |  |  |  |  |  |  |  |  |  |  |  |  |  |  |
| 1 | Global | 160121 (149585, 168193) | 35.70 (33.03, 37.62) | 411533 (365890, 446353) | 38.91 (34.44, 42.24) | 0.34 (0.31, 0.37) | 0.43 (0.38, 0.47) | 3157368 (2975127, 3308708) | 661.37 (619.76, 694.28) | 7643080 (6912849, 8256300) | 705.73 (636.52, 762.75) | 0.27 (0.23, 0.30) | 0.33 (-0.54, 1.20) | 152251 (142395, 159736) | 33.50 (31.05, 35.25) | 403853 (358257, 437883) | 38.05 (33.61, 41.30) | 0.49 (0.46, 0.53) | 0.63 (0.57, 0.68) | 112307 (105377, 117626) | 24.18 (22.51, 25.39) | 319701 (283635, 346991) | 29.96 (26.46, 32.56) | 0.84 (0.79, 0.90) | 1.34 (1.33, 1.36) |
| 2 | Male | 77403 (72916, 81427) | 39.55 (37.05, 41.62) | 209020 (189060, 227798) | 44.19 (39.80, 48.13) | 0.46 (0.42, 0.50) | 0.54 (0.46, 0.62) | 1611674 (1519652, 1698074) | 750.92 (706.23, 790.91) | 4073115 (3698320, 4446974) | 817.11 (740.20, 891.62) | 0.37 (0.33, 0.41) | 0.44 (-1.03, 1.90) | 74146 (69882, 77949) | 37.16 (34.84, 39.09) | 206100 (186283, 224635) | 43.22 (38.89, 47.08) | 0.60 (0.56, 0.65) | 0.73 (0.63, 0.83) | 55098 (52073, 57863) | 26.69 (25.12, 28.05) | 162562 (146946, 177885) | 33.62 (30.25, 36.81) | 0.93 (0.86, 0.99) | 1.50 (1.48, 1.51) |
| 3 | Female | 82718 (75559, 88216) | 32.45 (29.41, 34.70) | 202514 (172726, 222607) | 34.36 (29.33, 37.76) | 0.21 (0.18, 0.24) | 0.29 (0.26, 0.31) | 1545695 (1429453, 1645167) | 584.57 (538.15, 623.08) | 3569966 (3122569, 3895498) | 607.29 (531.42, 662.56) | 0.13 (0.10, 0.17) | 0.18 (-0.30, 0.66) | 78105 (71301, 83208) | 30.38 (27.54, 32.45) | 197753 (167834, 217191) | 33.57 (28.51, 36.86) | 0.36 (0.34, 0.39) | 0.50 (0.47, 0.54) | 57209 (52430, 60872) | 21.97 (20.02, 23.43) | 157139 (133870, 174109) | 26.71 (22.76, 29.58) | 0.75 (0.70, 0.81) | 1.18 (1.17, 1.20) |
| 4 | High SDI | 80686 (75109, 84312) | 55.66 (51.67, 58.24) | 183121 (159959, 197853) | 62.31 (55.02, 67.05) | 0.48 (0.43, 0.52) | 0.58 (0.41, 0.75) | 1505069 (1421773, 1562749) | 1038.26 (979.90, 1078.50) | 3162376 (2838463, 3383108) | 1130.73 (1023.00, 1205.97) | 0.39 (0.35, 0.44) | 0.55 (0.05, 1.04) | 78994 (73533, 82489) | 54.40 (50.53, 56.86) | 189260 (165005, 204509) | 64.85 (57.14, 69.80) | 0.70 (0.66, 0.75) | 1.10 (1.07, 1.13) | 61229 (57209, 63994) | 42.15 (39.33, 44.08) | 167745 (146213, 183492) | 58.34 (51.36, 63.52) | 1.29 (1.19, 1.38) | 1.98 (1.94, 2.02) |
| 5 | High-middle SDI | 49619 (46353, 52572) | 41.89 (38.88, 44.48) | 120706 (106124, 134358) | 47.74 (41.90, 53.12) | 0.47 (0.44, 0.51) | 0.61 (0.50, 0.71) | 1017937 (955582, 1077271) | 810.52 (758.33, 858.75) | 2306482 (2044046, 2570535) | 898.12 (795.15, 1000.46) | 0.36 (0.32, 0.40) | 0.46 (-1.60, 2.52) | 46015 (42993, 48728) | 38.24 (35.52, 40.57) | 114919 (100793, 128233) | 45.29 (39.65, 50.52) | 0.61 (0.57, 0.65) | 0.77 (0.66, 0.87) | 32300 (30234, 34173) | 26.21 (24.41, 27.77) | 82779 (72700, 92509) | 32.44 (28.44, 36.24) | 0.78 (0.73, 0.84) | 1.26 (1.24, 1.29) |
| 6 | Middle SDI | 21783 (19771, 23984) | 20.03 (18.15, 22.01) | 78471 (68147, 88725) | 24.91 (21.56, 28.18) | 0.69 (0.65, 0.72) | 1.08 (1.06, 1.09) | 461061 (418134, 508566) | 386.15 (350.18, 425.11) | 1566617 (1366895, 1768875) | 473.94 (412.78, 535.27) | 0.65 (0.61, 0.69) | 1.00 (0.68, 1.31) | 19905 (18057, 21936) | 17.80 (16.13, 19.57) | 72971 (63294, 82651) | 22.88 (19.78, 25.92) | 0.80 (0.76, 0.84) | 1.24 (1.22, 1.25) | 13734 (12452, 15152) | 11.80 (10.69, 12.99) | 50767 (44096, 57529) | 15.59 (13.51, 17.67) | 0.90 (0.86, 0.95) | 1.38 (1.37, 1.39) |
| 7 | Low-middle SDI | 5757 (4808, 6765) | 8.95 (7.47, 10.48) | 23187 (21111, 25288) | 14.35 (13.00, 15.65) | 1.62 (1.58, 1.65) | 2.19 (2.17, 2.20) | 123787 (103356, 145948) | 176.92 (147.73, 208.05) | 481187 (439391, 525527) | 280.31 (255.38, 306.07) | 1.55 (1.52, 1.58) | 2.70 (2.60, 2.81) | 5252 (4385, 6172) | 7.96 (6.64, 9.33) | 21195 (19304, 23108) | 12.88 (11.68, 14.05) | 1.64 (1.61, 1.67) | 2.30 (2.29, 2.31) | 3613 (3018, 4251) | 5.28 (4.41, 6.19) | 14621 (13327, 15956) | 8.66 (7.86, 9.45) | 1.68 (1.65, 1.71) | 2.39 (2.38, 2.40) |
| 8 | Low SDI | 2040 (1576, 2483) | 8.56 (6.59, 10.47) | 5582 (4625, 6701) | 10.70 (8.85, 12.88) | 0.73 (0.58, 0.88) | 1.20 (1.19, 1.21) | 44833 (34670, 54574) | 172.07 (132.84, 209.80) | 117618 (97401, 141331) | 208.08 (172.21, 250.24) | 0.59 (0.45, 0.74) | 0.99 (0.88, 1.11) | 1869 (1442, 2273) | 7.63 (5.87, 9.31) | 5075 (4207, 6066) | 9.49 (7.85, 11.37) | 0.71 (0.56, 0.86) | 1.17 (1.16, 1.18) | 1283 (990, 1560) | 5.03 (3.88, 6.14) | 3476 (2881, 4157) | 6.28 (5.20, 7.52) | 0.71 (0.56, 0.86) | 1.17 (1.17, 1.17) |
| 9 | Andean Latin America | 695 (560, 849) | 30.51 (24.60, 37.28) | 2566 (1940, 3255) | 36.25 (27.42, 45.97) | 0.56 (0.45, 0.66) | 1.13 (1.08, 1.17) | 13718 (11008, 16798) | 582.25 (467.55, 712.63) | 48756 (36716, 62157) | 679.69 (512.07, 866.15) | 0.47 (0.36, 0.57) | 1.02 (0.27, 1.77) | 620 (499, 756) | 26.95 (21.71, 32.86) | 2313 (1744, 2951) | 32.57 (24.56, 41.53) | 0.61 (0.50, 0.71) | 1.25 (1.21, 1.29) | 416 (334, 508) | 17.81 (14.33, 21.75) | 1571 (1183, 2010) | 21.99 (16.56, 28.11) | 0.68 (0.57, 0.78) | 1.41 (1.38, 1.43) |
| 10 | Australasia | 1481 (1332, 1628) | 48.51 (43.43, 53.46) | 3820 (3220, 4363) | 51.32 (43.48, 58.50) | 0.27 (0.19, 0.35) | 0.36 (0.23, 0.50) | 28182 (25562, 30867) | 907.37 (821.00, 995.09) | 65629 (56419, 74312) | 915.72 (790.44, 1035.25) | 0.11 (0.04, 0.18) | 0.15 (-2.06, 2.35) | 1510 (1357, 1666) | 49.28 (44.09, 54.49) | 4150 (3490, 4764) | 56.04 (47.35, 64.20) | 0.53 (0.45, 0.60) | 0.72 (0.67, 0.76) | 1242 (1106, 1385) | 40.29 (35.78, 45.01) | 4060 (3343, 4821) | 55.40 (45.87, 65.62) | 1.29 (1.18, 1.41) | 1.88 (1.84, 1.92) |
| 11 | Caribbean | 1029 (925, 1139) | 33.02 (29.62, 36.60) | 2259 (1923, 2614) | 33.45 (28.50, 38.70) | 0.56 (0.39, 0.72) | 0.45 (-0.98, 1.88) | 19269 (17350, 21326) | 601.76 (541.17, 666.41) | 42191 (35987, 48899) | 628.24 (535.95, 728.09) | 0.63 (0.47, 0.78) | 0.54 (-23.61, 24.68) | 922 (827, 1020) | 29.31 (26.25, 32.46) | 2041 (1742, 2359) | 30.30 (25.87, 35.01) | 0.62 (0.45, 0.79) | 0.52 (-0.75, 1.79) | 614 (551, 679) | 19.32 (17.34, 21.39) | 1393 (1189, 1608) | 20.72 (17.70, 23.93) | 0.74 (0.58, 0.91) | 0.67 (-0.20, 1.55) |
| 12 | Central Asia | 987 (857, 1171) | 18.03 (15.58, 21.53) | 2500 (2190, 2820) | 27.57 (24.14, 31.06) | 1.66 (1.46, 1.85) | 2.48 (2.42, 2.53) | 21342 (18703, 24990) | 373.58 (326.36, 439.54) | 53859 (47195, 60871) | 549.97 (482.13, 620.60) | 1.47 (1.29, 1.65) | 2.18 (1.00, 3.36) | 906 (790, 1071) | 16.36 (14.21, 19.45) | 2311 (2023, 2608) | 25.00 (21.88, 28.17) | 1.64 (1.45, 1.83) | 2.46 (2.41, 2.51) | 635 (556, 746) | 11.22 (9.80, 13.26) | 1639 (1434, 1850) | 17.06 (14.93, 19.23) | 1.61 (1.43, 1.80) | 2.40 (2.37, 2.43) |
| 13 | Central Europe | 9706 (9148, 10180) | 51.75 (48.54, 54.40) | 18847 (17007, 20523) | 61.61 (55.61, 67.10) | 0.57 (0.53, 0.61) | 0.87 (0.84, 0.91) | 197398 (187037, 206331) | 1007.52 (951.95, 1054.66) | 361736 (328579, 393082) | 1197.97 (1088.47, 1301.80) | 0.58 (0.54, 0.63) | 0.89 (0.20, 1.59) | 8852 (8346, 9277) | 46.58 (43.74, 48.91) | 17364 (15657, 18907) | 56.88 (51.31, 61.94) | 0.66 (0.61, 0.71) | 1.01 (0.98, 1.04) | 6084 (5747, 6371) | 31.42 (29.58, 32.96) | 12207 (11022, 13308) | 40.25 (36.35, 43.87) | 0.84 (0.78, 0.90) | 1.28 (1.26, 1.30) |
| 14 | Central Latin America | 2683 (2540, 2807) | 29.73 (28.04, 31.16) | 9114 (8112, 10171) | 30.33 (26.98, 33.84) | -0.12 (-0.23, -0.01) | 0.12 (-0.03, 0.28) | 53030 (50445, 55316) | 558.57 (530.32, 583.38) | 178711 (159813, 199636) | 581.03 (519.30, 648.94) | -0.08 (-0.20, 0.04) | 0.17 (-2.37, 2.71) | 2407 (2281, 2517) | 26.27 (24.81, 27.51) | 8282 (7372, 9255) | 27.41 (24.37, 30.62) | -0.06 (-0.17, 0.06) | 0.20 (0.06, 0.34) | 1625 (1543, 1697) | 17.36 (16.44, 18.16) | 5710 (5094, 6384) | 18.69 (16.66, 20.90) | 0.05 (-0.07, 0.17) | 0.32 (0.24, 0.40) |
| 15 | Central Sub-Saharan Africa | 318 (243, 410) | 14.29 (10.78, 18.91) | 771 (513, 1108) | 14.97 (9.84, 22.09) | 0.08 (-0.15, 0.31) | 0.19 (0.18, 0.20) | 7203 (5487, 9244) | 285.00 (216.34, 370.75) | 17045 (11404, 24237) | 294.74 (195.76, 425.84) | 0.04 (-0.19, 0.27) | 0.12 (-0.10, 0.34) | 294 (223, 377) | 12.68 (9.55, 16.61) | 707 (475, 1001) | 13.27 (8.81, 19.23) | 0.08 (-0.15, 0.31) | 0.19 (0.18, 0.20) | 204 (155, 262) | 8.32 (6.28, 10.86) | 495 (333, 697) | 8.80 (5.86, 12.64) | 0.12 (-0.12, 0.35) | 0.25 (0.24, 0.25) |
| 16 | East Asia | 25113 (21409, 29336) | 26.97 (23.00, 31.50) | 93256 (74169, 113425) | 34.76 (27.65, 42.20) | 0.80 (0.70, 0.89) | 1.18 (1.12, 1.24) | 540742 (459953, 631235) | 522.24 (444.58, 609.60) | 1832400 (1451056, 2238263) | 655.71 (519.45, 799.70) | 0.73 (0.65, 0.80) | 1.08 (-0.07, 2.23) | 23138 (19704, 27036) | 24.07 (20.51, 28.12) | 89235 (70772, 109064) | 32.98 (26.16, 40.22) | 1.03 (0.94, 1.11) | 1.52 (1.47, 1.57) | 16067 (13668, 18770) | 15.97 (13.59, 18.65) | 63035 (49877, 77272) | 22.91 (18.14, 28.03) | 1.20 (1.12, 1.27) | 1.88 (1.86, 1.90) |
| 17 | Eastern Europe | 14529 (13696, 15455) | 40.54 (38.12, 43.15) | 23897 (21757, 25958) | 50.32 (45.81, 54.65) | 0.66 (0.46, 0.86) | 0.83 (0.64, 1.02) | 311492 (294316, 331371) | 836.48 (789.40, 889.86) | 482806 (441259, 524750) | 999.96 (914.01, 1086.61) | 0.50 (0.31, 0.70) | 0.62 (-3.63, 4.88) | 13521 (12738, 14392) | 37.33 (35.10, 39.75) | 22390 (20377, 24312) | 46.96 (42.73, 50.98) | 0.71 (0.50, 0.92) | 0.89 (0.70, 1.07) | 9512 (8962, 10140) | 25.80 (24.27, 27.51) | 15750 (14347, 17129) | 32.74 (29.82, 35.60) | 0.74 (0.53, 0.96) | 0.94 (0.81, 1.06) |
| 18 | Eastern Sub-Saharan Africa | 849 (648, 1060) | 10.95 (8.36, 13.69) | 2290 (1860, 2918) | 13.64 (11.07, 17.30) | 0.64 (0.55, 0.73) | 1.13 (1.12, 1.14) | 18485 (14107, 23077) | 217.40 (165.99, 271.50) | 48417 (39234, 61965) | 264.70 (214.68, 337.45) | 0.54 (0.46, 0.63) | 1.00 (0.90, 1.10) | 776 (592, 969) | 9.72 (7.43, 12.14) | 2082 (1692, 2647) | 12.08 (9.82, 15.30) | 0.63 (0.54, 0.72) | 1.13 (1.13, 1.14) | 529 (404, 661) | 6.37 (4.87, 7.97) | 1429 (1160, 1823) | 7.99 (6.49, 10.15) | 0.66 (0.57, 0.75) | 1.16 (1.16, 1.16) |
| 19 | High-income Asia Pacific | 13994 (12912, 14763) | 57.69 (52.79, 61.06) | 45794 (37629, 50967) | 64.98 (54.90, 71.55) | 0.55 (0.44, 0.65) | 0.71 (0.57, 0.85) | 267023 (249685, 280387) | 1060.91 (987.32, 1116.08) | 719800 (612980, 790351) | 1140.66 (993.00, 1241.88) | 0.42 (0.31, 0.52) | 0.53 (-3.07, 4.13) | 14554 (13371, 15411) | 59.73 (54.45, 63.44) | 50301 (41240, 56202) | 71.97 (60.58, 79.58) | 0.81 (0.70, 0.92) | 1.06 (0.87, 1.24) | 12805 (11703, 13834) | 52.05 (47.26, 56.44) | 50036 (40071, 59177) | 72.75 (59.80, 84.79) | 1.44 (1.32, 1.56) | 1.65 (1.09, 2.21) |
| 20 | High-income North America | 27006 (24777, 28287) | 56.97 (52.26, 59.68) | 54892 (48880, 58307) | 61.03 (54.50, 64.75) | 0.27 (0.23, 0.30) | 0.37 (0.35, 0.39) | 503337 (470373, 523523) | 1075.65 (1006.10, 1118.54) | 1002778 (916099, 1055889) | 1128.84 (1033.23, 1187.76) | 0.19 (0.16, 0.22) | 0.26 (-0.10, 0.62) | 27382 (25099, 28675) | 57.84 (53.03, 60.57) | 58218 (51730, 61859) | 64.94 (57.84, 68.93) | 0.42 (0.38, 0.47) | 0.62 (0.60, 0.64) | 21373 (19751, 22390) | 45.40 (41.99, 47.55) | 49329 (44064, 52560) | 55.41 (49.59, 59.00) | 0.74 (0.65, 0.83) | 1.09 (1.06, 1.13) |
| 21 | North Africa and Middle East | 3278 (2583, 4036) | 18.76 (14.63, 23.17) | 14348 (12163, 16667) | 30.11 (25.41, 35.03) | 1.84 (1.65, 2.02) | 2.49 (2.42, 2.57) | 69137 (54946, 84845) | 359.94 (284.09, 442.64) | 291632 (248254, 338696) | 567.84 (481.89, 659.95) | 1.72 (1.56, 1.88) | 2.33 (0.87, 3.79) | 3016 (2380, 3692) | 16.80 (13.13, 20.63) | 13635 (11548, 15843) | 28.11 (23.70, 32.70) | 1.96 (1.78, 2.14) | 2.67 (2.60, 2.74) | 2077 (1645, 2540) | 11.09 (8.71, 13.60) | 9541 (8093, 11080) | 19.00 (16.05, 22.09) | 2.02 (1.85, 2.20) | 2.75 (2.71, 2.80) |
| 22 | Oceania | 34 (26, 44) | 12.27 (9.58, 16.15) | 106 (83, 139) | 15.15 (11.88, 19.75) | 0.79 (0.75, 0.83) | 1.33 (1.32, 1.33) | 737 (567, 970) | 234.75 (182.15, 309.04) | 2270 (1770, 2972) | 291.27 (227.72, 380.68) | 0.79 (0.75, 0.83) | 1.35 (1.26, 1.44) | 31 (24, 40) | 10.82 (8.46, 14.21) | 97 (76, 126) | 13.48 (10.57, 17.49) | 0.82 (0.77, 0.86) | 1.37 (1.37, 1.38) | 21 (16, 28) | 7.04 (5.49, 9.24) | 67 (53, 88) | 8.90 (6.97, 11.54) | 0.86 (0.82, 0.90) | 1.46 (1.46, 1.47) |
| 23 | South Asia | 3857 (2987, 4727) | 6.36 (4.91, 7.79) | 15665 (13818, 17534) | 9.30 (8.19, 10.43) | 1.09 (0.93, 1.25) | 2.08 (2.07, 2.09) | 85274 (66217, 104652) | 129.38 (100.22, 158.64) | 325340 (287224, 363886) | 181.92 (160.48, 203.66) | 0.93 (0.76, 1.10) | 1.90 (1.71, 2.10) | 3543 (2743, 4343) | 5.70 (4.39, 6.98) | 14300 (12611, 15999) | 8.34 (7.34, 9.34) | 1.08 (0.92, 1.25) | 2.09 (2.08, 2.10) | 2460 (1909, 3016) | 3.81 (2.95, 4.66) | 9861 (8696, 11026) | 5.60 (4.93, 6.27) | 1.09 (0.93, 1.26) | 2.13 (2.12, 2.13) |
| 24 | Southeast Asia | 3727 (3156, 4359) | 13.77 (11.66, 16.12) | 15655 (13153, 18678) | 21.40 (17.89, 25.56) | 1.39 (1.33, 1.45) | 2.10 (2.09, 2.11) | 80144 (67709, 93772) | 273.92 (231.62, 320.57) | 327846 (276734, 391168) | 415.39 (349.57, 495.73) | 1.31 (1.26, 1.37) | 1.97 (1.79, 2.16) | 3400 (2873, 3973) | 12.28 (10.37, 14.35) | 14437 (12173, 17201) | 19.34 (16.23, 23.07) | 1.44 (1.38, 1.50) | 2.18 (2.17, 2.19) | 2356 (1990, 2751) | 8.21 (6.94, 9.59) | 10179 (8612, 12120) | 13.17 (11.10, 15.69) | 1.50 (1.44, 1.56) | 2.28 (2.27, 2.28) |
| 25 | Southern Latin America | 3504 (3068, 3974) | 61.39 (53.60, 69.72) | 6774 (5924, 7604) | 59.20 (51.83, 66.44) | 0.13 (0.01, 0.24) | 0.17 (0.07, 0.27) | 68326 (60101, 77303) | 1158.15 (1017.14, 1311.36) | 125362 (110721, 140092) | 1109.90 (980.97, 1240.18) | 0.07 (-0.04, 0.18) | 0.09 (-1.66, 1.84) | 3166 (2778, 3590) | 54.89 (48.05, 62.31) | 6159 (5395, 6908) | 53.99 (47.34, 60.55) | 0.18 (0.07, 0.29) | 0.25 (0.16, 0.34) | 2139 (1879, 2425) | 36.62 (32.11, 41.54) | 4212 (3705, 4721) | 37.16 (32.71, 41.64) | 0.27 (0.16, 0.39) | 0.39 (0.33, 0.45) |
| 26 | Southern Sub-Saharan Africa | 702 (568, 907) | 24.17 (19.50, 31.22) | 2338 (2022, 2664) | 37.74 (32.51, 43.00) | 1.45 (1.15, 1.74) | 2.07 (1.99, 2.15) | 13918 (11307, 17992) | 447.49 (362.75, 578.18) | 47870 (41494, 54573) | 710.49 (614.73, 810.00) | 1.52 (1.21, 1.83) | 2.16 (0.58, 3.74) | 627 (509, 810) | 21.18 (17.12, 27.34) | 2122 (1834, 2419) | 33.43 (28.80, 38.11) | 1.49 (1.19, 1.78) | 2.12 (2.05, 2.19) | 421 (342, 545) | 13.81 (11.19, 17.86) | 1444 (1249, 1646) | 21.94 (18.93, 25.01) | 1.52 (1.23, 1.80) | 2.16 (2.11, 2.20) |
| 27 | Tropical Latin America | 3161 (2926, 3342) | 32.03 (29.35, 34.02) | 12285 (10997, 13206) | 39.14 (34.91, 42.12) | 0.75 (0.69, 0.81) | 1.26 (1.23, 1.30) | 64123 (59895, 67564) | 604.47 (561.19, 638.57) | 238024 (216974, 254238) | 741.41 (674.12, 792.66) | 0.72 (0.66, 0.78) | 1.27 (0.70, 1.83) | 2856 (2648, 3015) | 28.32 (26.02, 30.02) | 11097 (9964, 11914) | 35.16 (31.46, 37.79) | 0.79 (0.73, 0.85) | 1.34 (1.31, 1.37) | 1941 (1806, 2046) | 18.67 (17.24, 19.75) | 7596 (6870, 8137) | 23.82 (21.48, 25.54) | 0.87 (0.81, 0.93) | 1.47 (1.45, 1.49) |
| 28 | Western Europe | 42822 (39530, 45352) | 54.94 (50.64, 58.26) | 81731 (71281, 89028) | 61.71 (54.63, 66.83) | 0.57 (0.51, 0.63) | 0.71 (0.51, 0.92) | 780934 (731014, 822420) | 1015.72 (950.91, 1069.97) | 1375949 (1230254, 1484083) | 1125.10 (1018.15, 1207.76) | 0.53 (0.47, 0.59) | 0.69 (0.08, 1.30) | 40146 (37138, 42477) | 51.51 (47.61, 54.55) | 80230 (69925, 87458) | 61.52 (54.41, 66.68) | 0.79 (0.73, 0.85) | 1.16 (1.13, 1.19) | 29391 (27254, 31122) | 37.96 (35.20, 40.21) | 68525 (59637, 75604) | 54.49 (48.05, 59.78) | 1.48 (1.37, 1.60) | 2.23 (2.19, 2.27) |
| 29 | Western Sub-Saharan Africa | 647 (542, 749) | 6.83 (5.73, 7.91) | 2627 (2191, 3102) | 13.42 (11.24, 15.81) | 2.49 (2.39, 2.58) | 2.98 (2.95, 3.01) | 13554 (11338, 15729) | 133.31 (111.60, 154.58) | 54659 (45371, 64806) | 258.17 (214.92, 305.27) | 2.43 (2.34, 2.52) | 2.93 (2.25, 3.61) | 585 (490, 678) | 6.05 (5.06, 7.00) | 2381 (1976, 2805) | 11.89 (9.90, 13.97) | 2.49 (2.40, 2.58) | 2.99 (2.96, 3.02) | 395 (331, 459) | 3.96 (3.32, 4.59) | 1624 (1344, 1917) | 7.83 (6.50, 9.22) | 2.50 (2.41, 2.59) | 3.02 (3.00, 3.04) |
| Gallbladder and biliary tract cancer |  |  |  |  |  |  |  |  |  |  |  |  |  |  |  |  |  |  |  |  |  |  |  |  |  |
| 1 | Global | 77689 (69116, 86208) | 17.58 (15.57, 19.48) | 141955 (116087, 161769) | 13.55 (11.07, 15.44) | -0.92 (-0.95, -0.89) | -1.56 (-1.56, -1.55) | 1493528 (1329769, 1667537) | 316.69 (281.38, 352.94) | 2554711 (2091149, 2917769) | 237.51 (194.37, 271.15) | -1.02 (-1.05, -0.98) | -1.72 (-1.81, -1.63) | 82677 (74102, 90734) | 18.47 (16.47, 20.26) | 174216 (143068, 197555) | 16.55 (13.57, 18.78) | -0.41 (-0.44, -0.37) | -0.58 (-0.60, -0.57) | 95187 (86111, 102756) | 20.79 (18.71, 22.45) | 241947 (200600, 273107) | 22.80 (18.88, 25.74) | 0.30 (0.25, 0.35) | 0.42 (0.39, 0.46) |
| 2 | Male | 27691 (23599, 32378) | 14.84 (12.72, 17.19) | 62167 (46820, 72844) | 13.65 (10.37, 15.93) | -0.33 (-0.35, -0.31) | -0.39 (-0.41, -0.37) | 551996 (466854, 651224) | 266.99 (226.93, 312.66) | 1142501 (847461, 1348444) | 235.57 (175.95, 277.12) | -0.47 (-0.49, -0.44) | -0.40 (-4.74, 3.95) | 29908 (25971, 34104) | 15.68 (13.65, 17.74) | 79775 (61582, 92706) | 17.28 (13.44, 20.01) | 0.30 (0.28, 0.32) | 0.42 (0.41, 0.44) | 35674 (31728, 39646) | 18.12 (16.14, 20.02) | 118738 (93909, 137543) | 25.20 (20.04, 29.11) | 1.13 (1.08, 1.18) | 1.56 (1.52, 1.61) |
| 3 | Female | 49998 (43779, 56854) | 19.56 (17.05, 22.21) | 79788 (62027, 93802) | 13.53 (10.53, 15.91) | -1.28 (-1.32, -1.24) | -2.15 (-2.16, -2.15) | 941532 (826638, 1079053) | 355.52 (311.47, 407.03) | 1412210 (1101702, 1663118) | 240.31 (187.59, 283.08) | -1.37 (-1.42, -1.33) | -2.30 (-2.41, -2.18) | 52768 (46376, 59236) | 20.49 (17.93, 22.99) | 94441 (73807, 110149) | 16.02 (12.53, 18.69) | -0.88 (-0.92, -0.84) | -1.47 (-1.48, -1.46) | 59513 (52711, 65694) | 22.79 (20.11, 25.17) | 123209 (97848, 142749) | 20.94 (16.64, 24.25) | -0.33 (-0.38, -0.28) | -0.46 (-0.50, -0.43) |
| 4 | High SDI | 34649 (31405, 36696) | 23.86 (21.56, 25.30) | 48731 (40327, 54598) | 15.86 (13.26, 17.70) | -1.42 (-1.46, -1.38) | -2.24 (-2.25, -2.23) | 623516 (571647, 657613) | 428.57 (392.45, 452.26) | 756942 (641210, 839375) | 260.74 (222.40, 288.26) | -1.73 (-1.78, -1.69) | -2.75 (-2.98, -2.51) | 41191 (37403, 43612) | 28.30 (25.64, 29.99) | 70840 (59226, 79450) | 23.50 (19.85, 26.22) | -0.65 (-0.69, -0.62) | -0.95 (-0.98, -0.93) | 53565 (48973, 56491) | 36.64 (33.46, 38.66) | 115062 (97987, 127938) | 39.22 (33.70, 43.43) | 0.24 (0.17, 0.30) | 0.23 (0.20, 0.26) |
| 5 | High-middle SDI | 20782 (18005, 22470) | 18.12 (15.67, 19.62) | 32977 (25726, 38182) | 13.16 (10.27, 15.23) | -1.17 (-1.24, -1.10) | -1.40 (-1.45, -1.35) | 406747 (351616, 439807) | 331.78 (286.61, 359.09) | 603672 (468839, 701294) | 236.62 (183.88, 274.79) | -1.22 (-1.28, -1.16) | -1.49 (-2.50, -0.47) | 20981 (18344, 22595) | 17.93 (15.64, 19.35) | 42308 (32610, 49506) | 16.78 (12.95, 19.63) | -0.31 (-0.36, -0.26) | -0.54 (-0.55, -0.53) | 22276 (19673, 23957) | 18.50 (16.31, 19.93) | 60181 (45825, 70991) | 23.68 (18.05, 27.91) | 0.78 (0.70, 0.86) | 1.08 (1.03, 1.12) |
| 6 | Middle SDI | 14229 (12238, 18353) | 13.53 (11.60, 17.38) | 36029 (29083, 45761) | 11.63 (9.38, 14.78) | -0.59 (-0.64, -0.54) | -0.96 (-0.97, -0.96) | 292448 (251414, 377499) | 251.15 (215.65, 323.59) | 701106 (564576, 886568) | 214.68 (172.90, 271.65) | -0.61 (-0.67, -0.56) | -0.99 (-1.09, -0.90) | 13168 (11302, 16959) | 12.12 (10.36, 15.55) | 38724 (31085, 49837) | 12.28 (9.85, 15.81) | -0.03 (-0.11, 0.05) | -0.02 (-0.04, -0.01) | 12557 (10788, 16137) | 11.07 (9.49, 14.19) | 45438 (36363, 59320) | 14.08 (11.27, 18.36) | 0.74 (0.61, 0.87) | 1.09 (1.08, 1.11) |
| 7 | Low-middle SDI | 6336 (5281, 9045) | 10.06 (8.36, 14.32) | 19507 (15284, 24430) | 12.31 (9.66, 15.46) | 0.75 (0.69, 0.81) | 1.16 (1.16, 1.17) | 134160 (111898, 191870) | 194.38 (161.90, 277.59) | 395865 (308535, 493458) | 233.61 (182.38, 291.93) | 0.68 (0.62, 0.74) | 1.03 (0.92, 1.14) | 5781 (4823, 8212) | 8.91 (7.42, 12.65) | 18011 (14117, 22557) | 11.12 (8.72, 13.98) | 0.80 (0.75, 0.85) | 1.30 (1.30, 1.31) | 5353 (4473, 7574) | 7.95 (6.64, 11.24) | 17165 (13490, 21479) | 10.32 (8.11, 12.95) | 0.92 (0.88, 0.97) | 1.58 (1.58, 1.59) |
| 8 | Low SDI | 1572 (1233, 2195) | 6.74 (5.28, 9.47) | 4587 (3130, 5751) | 8.98 (6.12, 11.27) | 1.11 (1.02, 1.21) | 1.74 (1.73, 1.75) | 34341 (26880, 47950) | 133.33 (104.35, 186.62) | 94877 (64683, 119057) | 170.38 (116.12, 213.79) | 0.94 (0.88, 1.01) | 1.47 (1.37, 1.57) | 1439 (1128, 2005) | 5.98 (4.68, 8.37) | 4201 (2859, 5271) | 8.00 (5.44, 10.04) | 1.11 (1.03, 1.20) | 1.78 (1.77, 1.78) | 1318 (1034, 1836) | 5.26 (4.12, 7.35) | 3938 (2679, 4936) | 7.24 (4.92, 9.07) | 1.20 (1.13, 1.28) | 1.58 (1.56, 1.61) |
| 9 | Andean Latin America | 773 (592, 948) | 33.94 (26.05, 41.68) | 1860 (1361, 2514) | 26.26 (19.23, 35.47) | -1.01 (-1.15, -0.86) | -1.53 (-1.61, -1.46) | 15272 (11645, 18736) | 647.96 (494.66, 795.12) | 35489 (25900, 48368) | 494.49 (360.98, 673.49) | -1.09 (-1.24, -0.94) | -1.67 (-3.18, -0.15) | 695 (531, 854) | 30.19 (23.10, 37.15) | 1778 (1304, 2411) | 25.01 (18.34, 33.90) | -0.79 (-0.96, -0.63) | -1.19 (-1.26, -1.12) | 647 (495, 795) | 27.73 (21.27, 34.09) | 1802 (1326, 2459) | 25.23 (18.56, 34.40) | -0.50 (-0.68, -0.33) | -0.72 (-0.78, -0.65) |
| 10 | Australasia | 355 (320, 390) | 11.77 (10.53, 12.92) | 562 (473, 631) | 7.38 (6.25, 8.28) | -1.70 (-1.83, -1.56) | -2.61 (-2.64, -2.59) | 6557 (5955, 7156) | 211.82 (191.82, 231.46) | 9279 (7996, 10371) | 127.36 (110.30, 142.09) | -1.88 (-2.02, -1.73) | -3.02 (-3.32, -2.72) | 651 (583, 718) | 21.16 (18.88, 23.39) | 1558 (1268, 1834) | 20.91 (17.11, 24.57) | -0.05 (-0.24, 0.14) | -0.21 (-0.26, -0.15) | 1251 (1120, 1387) | 40.05 (35.76, 44.42) | 3965 (3217, 4695) | 54.24 (44.17, 64.11) | 1.07 (0.70, 1.43) | 1.29 (1.16, 1.43) |
| 11 | Caribbean | 328 (282, 374) | 10.57 (9.08, 12.08) | 408 (342, 481) | 6.05 (5.08, 7.12) | -2.01 (-2.13, -1.89) | -3.14 (-3.15, -3.12) | 6295 (5395, 7205) | 196.91 (168.68, 225.43) | 7754 (6472, 9168) | 115.55 (96.45, 136.65) | -1.94 (-2.08, -1.81) | -3.05 (-3.35, -2.74) | 308 (266, 352) | 9.83 (8.47, 11.23) | 401 (338, 470) | 5.96 (5.02, 6.99) | -1.84 (-1.96, -1.72) | -2.87 (-2.89, -2.86) | 301 (261, 343) | 9.47 (8.21, 10.80) | 419 (355, 490) | 6.25 (5.29, 7.30) | -1.59 (-1.71, -1.46) | -2.52 (-2.54, -2.51) |
| 12 | Central Asia | 368 (323, 431) | 6.83 (5.98, 8.02) | 493 (435, 561) | 5.55 (4.89, 6.30) | -1.08 (-1.53, -0.63) | -1.55 (-1.59, -1.51) | 7699 (6783, 9022) | 137.47 (120.86, 161.21) | 10376 (9147, 11814) | 108.38 (95.58, 123.27) | -1.20 (-1.62, -0.77) | -1.75 (-2.55, -0.96) | 343 (300, 402) | 6.30 (5.51, 7.39) | 468 (413, 532) | 5.17 (4.55, 5.87) | -1.05 (-1.51, -0.59) | -1.50 (-1.54, -1.47) | 327 (287, 384) | 5.92 (5.19, 6.95) | 459 (405, 522) | 4.92 (4.34, 5.60) | -1.00 (-1.48, -0.52) | -1.40 (-1.44, -1.37) |
| 13 | Central Europe | 5618 (5219, 5933) | 30.74 (28.42, 32.54) | 5356 (4804, 5876) | 17.42 (15.62, 19.11) | -2.09 (-2.17, -2.01) | -3.48 (-3.50, -3.45) | 108377 (100970, 114178) | 563.80 (523.91, 594.95) | 97271 (87930, 106531) | 319.89 (289.27, 350.26) | -2.05 (-2.13, -1.98) | -3.32 (-3.71, -2.93) | 5308 (4923, 5621) | 28.55 (26.37, 30.30) | 5619 (5013, 6197) | 18.30 (16.33, 20.18) | -1.68 (-1.76, -1.60) | -2.84 (-2.87, -2.82) | 5144 (4771, 5464) | 27.05 (25.02, 28.77) | 6401 (5687, 7134) | 20.90 (18.58, 23.29) | -1.03 (-1.10, -0.97) | -1.76 (-1.78, -1.74) |
| 14 | Central Latin America | 2605 (2459, 2733) | 29.01 (27.26, 30.50) | 4083 (3597, 4583) | 13.63 (12.00, 15.29) | -2.73 (-2.88, -2.58) | -4.33 (-4.35, -4.31) | 51138 (48661, 53523) | 540.25 (512.61, 566.16) | 78967 (69869, 88741) | 257.36 (227.66, 289.12) | -2.73 (-2.90, -2.57) | -4.34 (-4.71, -3.96) | 2369 (2238, 2485) | 25.94 (24.41, 27.25) | 3922 (3452, 4406) | 13.01 (11.44, 14.61) | -2.55 (-2.71, -2.39) | -4.05 (-4.07, -4.03) | 2236 (2118, 2341) | 23.96 (22.62, 25.12) | 4001 (3526, 4495) | 13.16 (11.59, 14.79) | -2.28 (-2.46, -2.11) | -3.62 (-3.64, -3.60) |
| 15 | Central Sub-Saharan Africa | 45 (30, 68) | 2.09 (1.39, 3.19) | 113 (73, 168) | 2.27 (1.45, 3.47) | 0.41 (0.29, 0.52) | 0.48 (0.48, 0.49) | 1000 (661, 1536) | 40.54 (26.92, 62.01) | 2439 (1599, 3609) | 43.38 (28.14, 64.99) | 0.35 (0.24, 0.46) | 0.40 (0.33, 0.47) | 41 (27, 63) | 1.84 (1.22, 2.80) | 103 (67, 153) | 2.01 (1.29, 3.03) | 0.42 (0.30, 0.54) | 0.50 (0.49, 0.50) | 38 (25, 58) | 1.60 (1.06, 2.44) | 97 (63, 143) | 1.79 (1.15, 2.68) | 0.50 (0.37, 0.63) | 0.60 (0.59, 0.60) |
| 16 | East Asia | 12783 (9800, 16529) | 14.57 (11.04, 18.71) | 31684 (22206, 41135) | 12.12 (8.46, 15.69) | -0.65 (-0.78, -0.52) | -0.91 (-0.94, -0.88) | 263326 (202449, 342339) | 265.78 (202.96, 343.85) | 594960 (419043, 777110) | 216.73 (152.21, 282.39) | -0.70 (-0.82, -0.58) | -1.02 (-1.54, -0.49) | 12054 (9236, 15529) | 13.19 (9.98, 16.90) | 40766 (28024, 52644) | 15.29 (10.48, 19.70) | 0.52 (0.42, 0.61) | 0.78 (0.76, 0.81) | 11687 (8943, 15044) | 12.10 (9.17, 15.49) | 57625 (38804, 74679) | 21.06 (14.16, 27.22) | 1.98 (1.85, 2.10) | 2.36 (2.28, 2.44) |
| 17 | Eastern Europe | 3350 (3090, 3643) | 9.56 (8.79, 10.40) | 4009 (3612, 4380) | 8.53 (7.68, 9.32) | -0.82 (-1.07, -0.58) | -0.65 (-0.81, -0.49) | 68883 (63703, 75001) | 188.47 (174.03, 205.15) | 76768 (69505, 83959) | 160.64 (145.42, 175.74) | -1.00 (-1.25, -0.75) | -0.87 (-3.93, 2.20) | 3563 (3296, 3865) | 10.04 (9.26, 10.89) | 5362 (4839, 5838) | 11.36 (10.25, 12.37) | 0.01 (-0.24, 0.26) | 0.34 (0.31, 0.38) | 3937 (3652, 4265) | 10.89 (10.08, 11.80) | 7737 (6994, 8409) | 16.27 (14.71, 17.69) | 1.03 (0.70, 1.36) | 2.25 (2.21, 2.30) |
| 18 | Eastern Sub-Saharan Africa | 542 (375, 739) | 7.05 (4.92, 9.56) | 1114 (777, 1487) | 6.85 (4.77, 9.15) | -0.18 (-0.26, -0.11) | -0.22 (-0.22, -0.22) | 11740 (8030, 16090) | 139.00 (95.80, 189.58) | 22838 (15943, 30533) | 128.00 (89.24, 171.06) | -0.38 (-0.45, -0.30) | -0.52 (-0.57, -0.47) | 495 (341, 673) | 6.24 (4.33, 8.45) | 1010 (706, 1346) | 6.03 (4.20, 8.04) | -0.21 (-0.29, -0.13) | -0.26 (-0.26, -0.25) | 447 (307, 606) | 5.43 (3.76, 7.33) | 936 (655, 1249) | 5.38 (3.76, 7.18) | -0.11 (-0.20, -0.03) | -0.10 (-0.10, -0.10) |
| 19 | High-income Asia Pacific | 13745 (12350, 14820) | 57.54 (51.28, 62.20) | 26631 (21293, 30470) | 34.69 (28.38, 39.48) | -1.77 (-1.81, -1.74) | -2.75 (-2.79, -2.72) | 251094 (228079, 270512) | 1008.70 (911.91, 1088.13) | 376934 (310117, 428236) | 552.39 (462.46, 625.93) | -2.09 (-2.13, -2.05) | -3.22 (-3.91, -2.54) | 15303 (13711, 16551) | 63.52 (56.48, 68.90) | 34777 (27607, 40541) | 46.56 (37.82, 53.93) | -1.06 (-1.11, -1.01) | -1.51 (-1.61, -1.42) | 18182 (16344, 19712) | 74.25 (66.34, 80.68) | 48907 (39048, 57633) | 68.67 (55.76, 80.43) | -0.21 (-0.30, -0.12) | -0.27 (-0.54, -0.01) |
| 20 | High-income North America | 4437 (4011, 4676) | 9.32 (8.42, 9.83) | 5262 (4627, 5639) | 5.82 (5.13, 6.23) | -1.55 (-1.66, -1.43) | -2.39 (-2.40, -2.38) | 78790 (72870, 82480) | 166.95 (154.50, 174.77) | 94074 (84866, 99968) | 105.51 (95.39, 112.03) | -1.52 (-1.64, -1.40) | -2.36 (-2.49, -2.23) | 7633 (6909, 8072) | 16.03 (14.51, 16.96) | 11855 (10463, 12750) | 13.18 (11.67, 14.17) | -0.68 (-0.73, -0.62) | -1.04 (-1.05, -1.02) | 13683 (12491, 14469) | 28.75 (26.26, 30.40) | 26492 (23636, 28420) | 29.70 (26.54, 31.85) | 0.06 (-0.03, 0.16) | 0.24 (0.12, 0.37) |
| 21 | North Africa and Middle East | 1604 (1264, 2182) | 9.49 (7.43, 13.01) | 4117 (3008, 5153) | 8.94 (6.54, 11.19) | 0.06 (-0.05, 0.18) | 0.07 (0.02, 0.13) | 32684 (25793, 44101) | 175.06 (137.75, 237.68) | 80341 (58429, 100487) | 160.48 (117.04, 200.88) | -0.08 (-0.18, 0.03) | -0.08 (-1.04, 0.88) | 1477 (1166, 2002) | 8.48 (6.66, 11.57) | 4131 (3008, 5171) | 8.76 (6.39, 10.97) | 0.35 (0.23, 0.48) | 0.44 (0.39, 0.48) | 1400 (1107, 1880) | 7.72 (6.08, 10.43) | 4394 (3203, 5484) | 9.01 (6.58, 11.26) | 0.73 (0.60, 0.86) | 0.92 (0.88, 0.97) |
| 22 | Oceania | 11 (7, 15) | 4.13 (2.55, 5.52) | 24 (16, 32) | 3.41 (2.31, 4.58) | -0.64 (-0.67, -0.61) | -0.96 (-0.96, -0.96) | 247 (148, 333) | 78.81 (47.92, 105.71) | 507 (343, 686) | 65.20 (44.10, 87.85) | -0.63 (-0.66, -0.60) | -0.93 (-0.97, -0.88) | 10 (6, 14) | 3.67 (2.25, 4.93) | 22 (15, 29) | 3.06 (2.07, 4.09) | -0.60 (-0.64, -0.57) | -0.90 (-0.90, -0.90) | 10 (6, 13) | 3.26 (2.00, 4.39) | 21 (14, 28) | 2.80 (1.89, 3.74) | -0.51 (-0.54, -0.48) | -0.75 (-0.75, -0.75) |
| 23 | South Asia | 6333 (5069, 9172) | 10.82 (8.65, 15.72) | 24589 (17333, 29409) | 14.87 (10.51, 17.85) | 1.10 (1.01, 1.18) | 1.76 (1.75, 1.77) | 137325 (109842, 198564) | 212.75 (170.06, 308.16) | 503931 (353806, 601333) | 284.74 (200.35, 340.44) | 0.99 (0.92, 1.06) | 1.59 (1.44, 1.75) | 5804 (4655, 8335) | 9.61 (7.69, 13.85) | 22833 (16119, 27273) | 13.50 (9.55, 16.18) | 1.15 (1.08, 1.22) | 1.95 (1.94, 1.95) | 5376 (4314, 7695) | 8.54 (6.84, 12.26) | 21909 (15466, 26147) | 12.60 (8.91, 15.07) | 1.31 (1.25, 1.37) | 2.32 (2.31, 2.32) |
| 24 | Southeast Asia | 2902 (2152, 3820) | 11.09 (8.20, 14.65) | 8580 (6093, 11387) | 12.20 (8.64, 16.18) | 0.11 (0.03, 0.19) | 0.42 (0.41, 0.42) | 60286 (44768, 79054) | 211.46 (156.85, 278.02) | 171381 (122057, 228029) | 224.28 (159.41, 298.06) | 0.00 (-0.07, 0.07) | 0.18 (0.06, 0.30) | 2684 (1985, 3547) | 9.98 (7.35, 13.23) | 8761 (6188, 11644) | 12.15 (8.55, 16.14) | 0.46 (0.38, 0.54) | 1.07 (1.06, 1.07) | 2572 (1895, 3385) | 9.24 (6.79, 12.19) | 9656 (6601, 13004) | 12.96 (8.86, 17.42) | 0.96 (0.87, 1.05) | 1.94 (1.94, 1.95) |
| 25 | Southern Latin America | 3190 (2853, 3517) | 56.47 (50.26, 62.44) | 3291 (2873, 3707) | 28.76 (25.14, 32.38) | -2.24 (-2.31, -2.16) | -3.08 (-3.24, -2.92) | 60896 (54870, 66839) | 1038.71 (933.28, 1141.90) | 60785 (53608, 68107) | 538.52 (475.19, 603.15) | -2.18 (-2.25, -2.11) | -2.93 (-5.63, -0.23) | 2991 (2684, 3296) | 52.26 (46.69, 57.70) | 3499 (3036, 3951) | 30.69 (26.66, 34.64) | -1.79 (-1.86, -1.72) | -2.45 (-2.59, -2.30) | 2903 (2609, 3196) | 49.88 (44.69, 55.01) | 4008 (3477, 4560) | 35.34 (30.68, 40.19) | -1.18 (-1.25, -1.11) | -0.96 (-3.34, 1.42) |
| 26 | Southern Sub-Saharan Africa | 129 (91, 176) | 4.51 (3.15, 6.10) | 335 (228, 404) | 5.51 (3.70, 6.64) | 0.76 (0.58, 0.94) | 1.17 (1.15, 1.18) | 2516 (1790, 3437) | 81.71 (57.81, 111.33) | 6725 (4611, 8112) | 101.28 (68.91, 122.09) | 0.82 (0.63, 1.02) | 1.24 (0.94, 1.53) | 116 (82, 158) | 3.96 (2.77, 5.38) | 309 (210, 372) | 4.94 (3.33, 5.95) | 0.83 (0.65, 1.00) | 1.25 (1.24, 1.26) | 108 (76, 147) | 3.57 (2.51, 4.85) | 289 (197, 349) | 4.46 (3.01, 5.37) | 0.84 (0.71, 0.97) | 1.25 (1.24, 1.27) |
| 27 | Tropical Latin America | 2081 (1913, 2214) | 21.40 (19.44, 22.88) | 4499 (3996, 4868) | 14.38 (12.73, 15.58) | -1.39 (-1.52, -1.26) | -2.17 (-2.19, -2.15) | 41514 (38542, 43967) | 395.28 (364.37, 419.90) | 85723 (77710, 92102) | 267.76 (242.08, 287.98) | -1.41 (-1.55, -1.27) | -2.21 (-2.54, -1.87) | 1903 (1753, 2023) | 19.09 (17.39, 20.38) | 4252 (3784, 4593) | 13.50 (11.98, 14.60) | -1.25 (-1.39, -1.11) | -1.93 (-1.95, -1.91) | 1795 (1660, 1905) | 17.45 (16.00, 18.58) | 4255 (3819, 4583) | 13.40 (11.99, 14.46) | -1.01 (-1.16, -0.86) | -1.52 (-1.53, -1.50) |
| 28 | Western Europe | 16462 (14838, 17553) | 20.99 (18.89, 22.40) | 14871 (12685, 16328) | 10.78 (9.35, 11.77) | -2.25 (-2.42, -2.08) | -3.52 (-3.54, -3.50) | 287290 (262669, 304525) | 370.19 (338.50, 392.42) | 236681 (207149, 257421) | 186.52 (165.49, 201.80) | -2.32 (-2.49, -2.15) | -3.62 (-3.99, -3.25) | 18901 (17086, 20247) | 24.07 (21.73, 25.79) | 22723 (19419, 25030) | 16.94 (14.69, 18.55) | -1.19 (-1.32, -1.07) | -1.83 (-1.87, -1.80) | 23121 (20984, 24756) | 29.41 (26.70, 31.49) | 38512 (33290, 42395) | 29.69 (25.99, 32.53) | 0.04 (-0.05, 0.14) | 0.09 (0.00, 0.17) |
| 29 | Western Sub-Saharan Africa | 29 (23, 43) | 0.31 (0.25, 0.46) | 75 (50, 94) | 0.40 (0.26, 0.50) | 1.47 (1.11, 1.83) | 1.58 (1.58, 1.58) | 599 (480, 872) | 5.95 (4.77, 8.72) | 1487 (1015, 1894) | 7.28 (4.88, 9.18) | 1.28 (0.92, 1.65) | 1.33 (1.29, 1.37) | 26 (21, 38) | 0.27 (0.22, 0.41) | 67 (45, 85) | 0.35 (0.23, 0.44) | 1.45 (1.09, 1.81) | 1.55 (1.54, 1.55) | 24 (19, 35) | 0.24 (0.19, 0.35) | 62 (42, 78) | 0.31 (0.21, 0.39) | 1.46 (1.09, 1.83) | 1.56 (1.56, 1.56) |

ALY disability-adjusted life year, ASR age standardized rate, ASIR age-standardized incidence rate, ASDR age-standardized DALYs rate,

ASMR age-standardized death rate, ASPR age-standardized prevalence rate, EAPC estimated annual percentage change, CI confidence interval,

UI uncertainty interval, SDI socio-demographic index

**Explanation of statistical method**

**Das Gupta Decomposition**


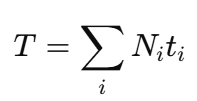


Where:

i represents the age group (or other grouping units),

Ni is the population size of that age group,

ri is the rate for that age group (such as disease rate, death rate, disease burden rate, or DALYs),

T is the total count (such as total deaths, total disease burden, total DALYs).

In two time periods t_0_ and t_1_,
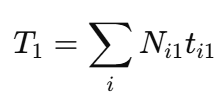


The change in T is:
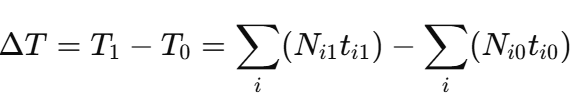


Das Gupta suggests decomposing the total change ΔT into three parts:

1. **Population Growth Effect**:


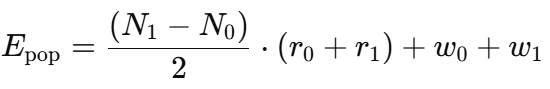


1. **Age Structure Effect**:


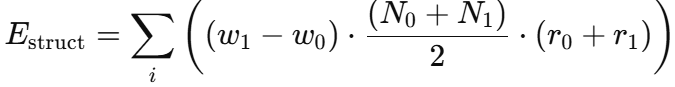


1. **Epidemiological Effect**:


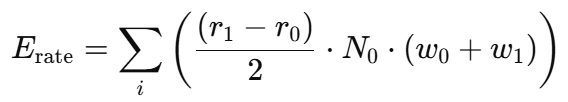


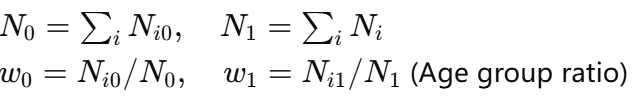


**Final Relationship**:


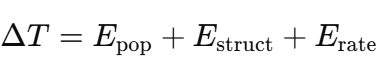


**TableS2 Decomposition analysis of GDSC**

|  | location | sex | Overall Difference | Aging | Population | Epidemiological Change | Aging Percentage | Population Percentage | Epidemiological Change Percentage |
| --- | --- | --- | --- | --- | --- | --- | --- | --- | --- |
| Incidence | Global | Both | 5477177954 | -60083846.68 | 3104757911 | 2432503890 | -1.1 | 56.69 | 44.41 |
|  | High SDI | Both | 361106815.3 | -67463573.05 | 272499380.3 | 156071008 | -18.68 | 75.46 | 43.22 |
|  | High-middle SDI | Both | 378917434.5 | -36236788.02 | 234946118.6 | 180208103.9 | -9.56 | 62 | 47.56 |
|  | Middle SDI | Both | 542078072.5 | 60056008.43 | 242789132.6 | 239232931.5 | 11.08 | 44.79 | 44.13 |
|  | Low-middle SDI | Both | 72064499.93 | 3314921.71 | 32940140.44 | 35809437.78 | 4.6 | 45.71 | 49.69 |
|  | Low SDI | Both | 7662154.37 | -186512.44 | 4570946.34 | 3277720.48 | -2.43 | 59.66 | 42.78 |
| Prevalence | Global | Both | 18632176018 | -248597162.4 | 9132183386 | 9748589794 | -1.33 | 49.01 | 52.32 |
|  | High SDI | Both | 1552516452 | -273068102.2 | 1036729868 | 788854687.1 | -17.59 | 66.78 | 50.81 |
|  | High-middle SDI | Both | 1375043443 | -113631350.7 | 670155078.6 | 818519715.2 | -8.26 | 48.74 | 59.53 |
|  | Middle SDI | Both | 1544400765 | 136648195.7 | 575342102.6 | 832410466.5 | 8.85 | 37.25 | 53.9 |
|  | Low-middle SDI | Both | 139996428.3 | 5373121.79 | 59161988.36 | 75461318.12 | 3.84 | 42.26 | 53.9 |
|  | Low SDI | Both | 12868703.22 | -343717.15 | 7173009.68 | 6039410.69 | -2.67 | 55.74 | 46.93 |
| DALYs | Global | Both | 78809485699 | -1950802988 | 53056941335 | 27703347352 | -2.48 | 67.32 | 35.15 |
|  | High SDI | Both | 3661815074 | -910288153.4 | 3475625372 | 1096477855 | -24.86 | 94.92 | 29.94 |
|  | High-middle SDI | Both | 4963209639 | -707024321.4 | 4087275480 | 1582958480 | -14.25 | 82.35 | 31.89 |
|  | Middle SDI | Both | 8886950103 | 1060041183 | 4713104181 | 3113804739 | 11.93 | 53.03 | 35.04 |
|  | Low-middle SDI | Both | 1618902132 | 68274412.2 | 775339741.8 | 775287977.9 | 4.22 | 47.89 | 47.89 |
|  | Low SDI | Both | 183273579.8 | -5451340.51 | 113200867.6 | 75524052.74 | -2.97 | 61.77 | 41.21 |
| Death | Global | Both | 3557499819 | -31765251.63 | 2299857083 | 1289407987 | -0.89 | 64.65 | 36.24 |
|  | High SDI | Both | 184942459.8 | -37924884.12 | 163502377 | 59364966.95 | -20.51 | 88.41 | 32.1 |
|  | High-middle SDI | Both | 227812670.6 | -25367633.6 | 176566179.5 | 76614124.7 | -11.14 | 77.5 | 33.63 |
|  | Middle SDI | Both | 390144914.9 | 50345952.44 | 199226923.1 | 140572039.3 | 12.9 | 51.06 | 36.03 |
|  | Low-middle SDI | Both | 67926124.71 | 3375946.87 | 31922079.64 | 32628098.2 | 4.97 | 47 | 48.03 |
|  | Low SDI | Both | 7636986.95 | -172668.87 | 4628699.57 | 3180956.26 | -2.26 | 60.61 | 41.65 |

**TableS3 Frontier of GDSC-related DALYs and effective difference by country (TOP15)**

| Effective difference rank | Location | Age-standardized DALYs | SDI | Frontier DALYs | Effective difference |
| --- | --- | --- | --- | --- | --- |
| Total GDSC |  |  |  |  |  |
| 1 | Mongolia | 8297.29 | 0.62 | 1372.47 | 6924.82 |
| 2 | Cabo Verde | 8203.68 | 0.53 | 1372.97 | 6830.71 |
| 3 | Greenland | 7929.60 | 0.83 | 1372.59 | 6557.01 |
| 4 | Zimbabwe | 7864.86 | 0.47 | 1373.11 | 6491.76 |
| 5 | Bolivia (Plurinational State of) | 7826.61 | 0.60 | 1372.65 | 6453.96 |
| 6 | Eswatini | 7518.26 | 0.59 | 1373.59 | 6144.67 |
| 7 | Uruguay | 7489.10 | 0.72 | 1373.17 | 6115.93 |
| 8 | China | 7364.86 | 0.72 | 1372.53 | 5992.33 |
| 9 | Lesotho | 7141.67 | 0.51 | 1373.05 | 5768.62 |
| 10 | Monaco | 6916.31 | 0.91 | 1372.75 | 5543.56 |
| 11 | Afghanistan | 7321.29 | 0.34 | 1970.22 | 5351.07 |
| 12 | Slovakia | 6642.71 | 0.81 | 1373.17 | 5269.55 |
| 13 | North Macedonia | 6636.97 | 0.75 | 1372.54 | 5264.43 |
| 14 | Japan | 6586.35 | 0.87 | 1373.05 | 5213.31 |
| 15 | Bulgaria | 6535.66 | 0.77 | 1373.43 | 5162.23 |
| Esophageal cancer |  |  |  |  |  |
| 1 | Malawi | 3163.31 | 0.38 | 102.36 | 3060.95 |
| 2 | Mongolia | 2217.51 | 0.62 | 84.85 | 2132.66 |
| 3 | Zambia | 1962.16 | 0.51 | 86.81 | 1875.35 |
| 4 | Zimbabwe | 1944.18 | 0.47 | 89.28 | 1854.90 |
| 5 | Cabo Verde | 1848.53 | 0.53 | 85.48 | 1763.04 |
| 6 | Uganda | 1852.00 | 0.42 | 97.27 | 1754.73 |
| 7 | South Sudan | 1909.83 | 0.28 | 155.46 | 1754.37 |
| 8 | Eswatini | 1834.74 | 0.59 | 85.17 | 1749.57 |
| 9 | Lesotho | 1805.39 | 0.51 | 86.52 | 1718.87 |
| 10 | Eritrea | 1776.62 | 0.40 | 99.61 | 1677.01 |
| 11 | China | 1698.83 | 0.72 | 83.63 | 1615.21 |
| 12 | Comoros | 1574.22 | 0.48 | 88.13 | 1486.09 |
| 13 | Djibouti | 1532.34 | 0.49 | 87.45 | 1444.89 |
| 14 | Kenya | 1516.45 | 0.52 | 85.40 | 1431.05 |
| 15 | United Republic of Tanzania | 1428.13 | 0.45 | 96.84 | 1331.29 |
| Gastric cancer |  |  |  |  |  |
| 1 | Mongolia | 4369.56 | 0.62 | 302.75 | 4066.81 |
| 2 | Bolivia (Plurinational State of) | 3831.03 | 0.60 | 302.14 | 3528.90 |
| 3 | Afghanistan | 3786.37 | 0.34 | 395.59 | 3390.78 |
| 4 | Cabo Verde | 2846.39 | 0.53 | 309.21 | 2537.18 |
| 5 | Guatemala | 2639.09 | 0.54 | 308.09 | 2331.00 |
| 6 | China | 2473.92 | 0.72 | 302.14 | 2171.78 |
| 7 | Honduras | 2407.48 | 0.51 | 309.36 | 2098.13 |
| 8 | Democratic People's Republic of Korea | 2351.64 | 0.57 | 302.71 | 2048.93 |
| 9 | Dominica | 2345.68 | 0.75 | 301.84 | 2043.84 |
| 10 | Peru | 2267.51 | 0.66 | 301.26 | 1966.24 |
| 11 | Yemen | 2268.00 | 0.45 | 318.95 | 1949.05 |
| 12 | Kiribati | 2244.81 | 0.53 | 309.24 | 1935.57 |
| 13 | Haiti | 2151.48 | 0.45 | 319.15 | 1832.33 |
| 14 | Nauru | 2109.63 | 0.63 | 302.36 | 1807.27 |
| 15 | Ecuador | 2022.71 | 0.66 | 301.95 | 1720.76 |
| Colon and rectum cancer |  |  |  |  |  |
| 1 | Hungary | 3259.50 | 0.79 | 313.46 | 2946.04 |
| 2 | Uruguay | 3222.27 | 0.72 | 313.58 | 2908.70 |
| 3 | Bulgaria | 3155.05 | 0.77 | 313.65 | 2841.40 |
| 4 | Slovakia | 3135.18 | 0.81 | 313.54 | 2821.64 |
| 5 | Croatia | 2998.75 | 0.80 | 313.59 | 2685.16 |
| 6 | Monaco | 2981.47 | 0.91 | 313.50 | 2667.97 |
| 7 | Poland | 2970.41 | 0.81 | 313.62 | 2656.79 |
| 8 | Greenland | 2822.95 | 0.83 | 313.98 | 2508.97 |
| 9 | Barbados | 2740.34 | 0.75 | 313.76 | 2426.58 |
| 10 | Serbia | 2672.36 | 0.79 | 313.52 | 2358.84 |
| 11 | Romania | 2637.05 | 0.77 | 313.51 | 2323.54 |
| 12 | Netherlands | 2528.06 | 0.89 | 313.74 | 2214.33 |
| 13 | Czechia | 2510.85 | 0.83 | 313.54 | 2197.31 |
| 14 | Taiwan (Province of China) | 2438.21 | 0.87 | 313.62 | 2124.59 |
| 15 | Republic of Moldova | 2382.28 | 0.73 | 313.67 | 2068.61 |
| Liver cancer |  |  |  |  |  |
| 1 | Mongolia | 9484.50 | 0.62 | 39.94 | 9444.56 |
| 2 | Gambia | 3247.07 | 0.41 | 49.99 | 3197.07 |
| 3 | Mali | 3053.39 | 0.27 | 190.63 | 2862.76 |
| 4 | Mozambique | 2868.77 | 0.33 | 190.44 | 2678.33 |
| 5 | Egypt | 2404.73 | 0.61 | 39.85 | 2364.87 |
| 6 | Mauritania | 2193.13 | 0.50 | 50.14 | 2143.00 |
| 7 | Tonga | 2097.13 | 0.63 | 40.41 | 2056.71 |
| 8 | Qatar | 1981.82 | 0.85 | 40.22 | 1941.61 |
| 9 | Eswatini | 1967.98 | 0.59 | 39.75 | 1928.23 |
| 10 | Guinea | 2041.50 | 0.34 | 190.47 | 1851.02 |
| 11 | Guinea-Bissau | 1962.06 | 0.35 | 189.89 | 1772.17 |
| 12 | Zimbabwe | 1742.25 | 0.47 | 50.10 | 1692.15 |
| 13 | Liberia | 1857.83 | 0.35 | 189.99 | 1667.83 |
| 14 | Burkina Faso | 1812.98 | 0.29 | 190.54 | 1622.44 |
| 15 | Lesotho | 1602.09 | 0.51 | 50.00 | 1552.09 |
| Pancreatic cancer |  |  |  |  |  |
| 1 | Greenland | 1874.07 | 0.83 | 21.04 | 1853.03 |
| 2 | Uruguay | 1612.66 | 0.72 | 21.09 | 1591.57 |
| 3 | Monaco | 1584.85 | 0.91 | 21.60 | 1563.25 |
| 4 | United Arab Emirates | 1493.48 | 0.85 | 21.20 | 1472.27 |
| 5 | Czechia | 1471.98 | 0.83 | 21.65 | 1450.33 |
| 6 | Hungary | 1389.10 | 0.79 | 21.12 | 1367.98 |
| 7 | Finland | 1363.65 | 0.86 | 21.77 | 1341.88 |
| 8 | Germany | 1299.92 | 0.90 | 21.46 | 1278.46 |
| 9 | Denmark | 1284.38 | 0.90 | 21.32 | 1263.05 |
| 10 | Bulgaria | 1283.43 | 0.77 | 21.07 | 1262.36 |
| 11 | Latvia | 1277.19 | 0.83 | 21.24 | 1255.96 |
| 12 | Estonia | 1270.23 | 0.84 | 21.27 | 1248.96 |
| 13 | Montenegro | 1265.71 | 0.80 | 21.28 | 1244.43 |
| 14 | Armenia | 1257.40 | 0.70 | 22.14 | 1235.26 |
| 15 | Austria | 1245.72 | 0.85 | 21.10 | 1224.62 |
| GBCT |  |  |  |  |  |
| 1 | Chile | 917.75 | 0.77 | 0.00 | 917.75 |
| 2 | Thailand | 773.57 | 0.68 | 0.00 | 773.56 |
| 3 | Bolivia (Plurinational State of) | 731.40 | 0.60 | 0.00 | 731.39 |
| 4 | Republic of Korea | 689.29 | 0.89 | 0.00 | 689.28 |
| 5 | United Arab Emirates | 529.17 | 0.85 | 0.00 | 529.16 |
| 6 | Japan | 525.26 | 0.87 | 0.00 | 525.25 |
| 7 | Peru | 497.06 | 0.66 | 0.00 | 497.05 |
| 8 | Uruguay | 467.97 | 0.72 | 0.00 | 467.96 |
| 9 | Slovakia | 465.63 | 0.81 | 0.00 | 465.62 |
| 10 | Czechia | 446.95 | 0.83 | 0.00 | 446.94 |
| 11 | Libya | 435.42 | 0.73 | 0.00 | 435.42 |
| 12 | Pakistan | 420.51 | 0.50 | 0.00 | 420.51 |
| 13 | Honduras | 411.06 | 0.51 | 0.00 | 411.05 |
| 14 | Bosnia and Herzegovina | 379.65 | 0.72 | 0.00 | 379.64 |
| 15 | Argentina | 371.63 | 0.72 | 0.00 | 371.62 |


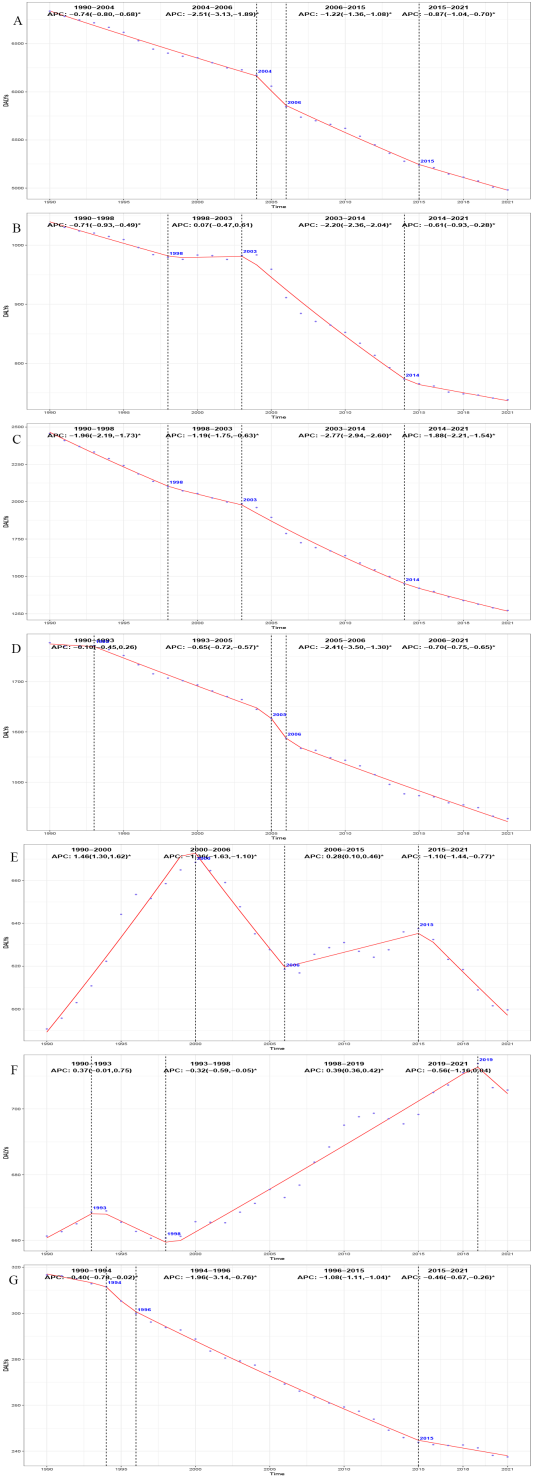


**FigureS1** Temporal trend changes in ASDR for GDSC (A), Geriatric Esophageal Cancer (B), Geriatric Gastric Cancer(C), Geriatric Colorectal Cancer(D), Geriatric Liver Cancer(E), Geriatric Pancreatic Cancer(F) and Geriatric CBCT(G) globally and in various SDI regions from 1990 to 2021 based on the Joinpoint regression model. *p < 0.05; GDSC, Geriatric Digestive System Cancers; SDI, sociodemographic index; ASDR, age-standardized disability-adjusted life years rate.

**
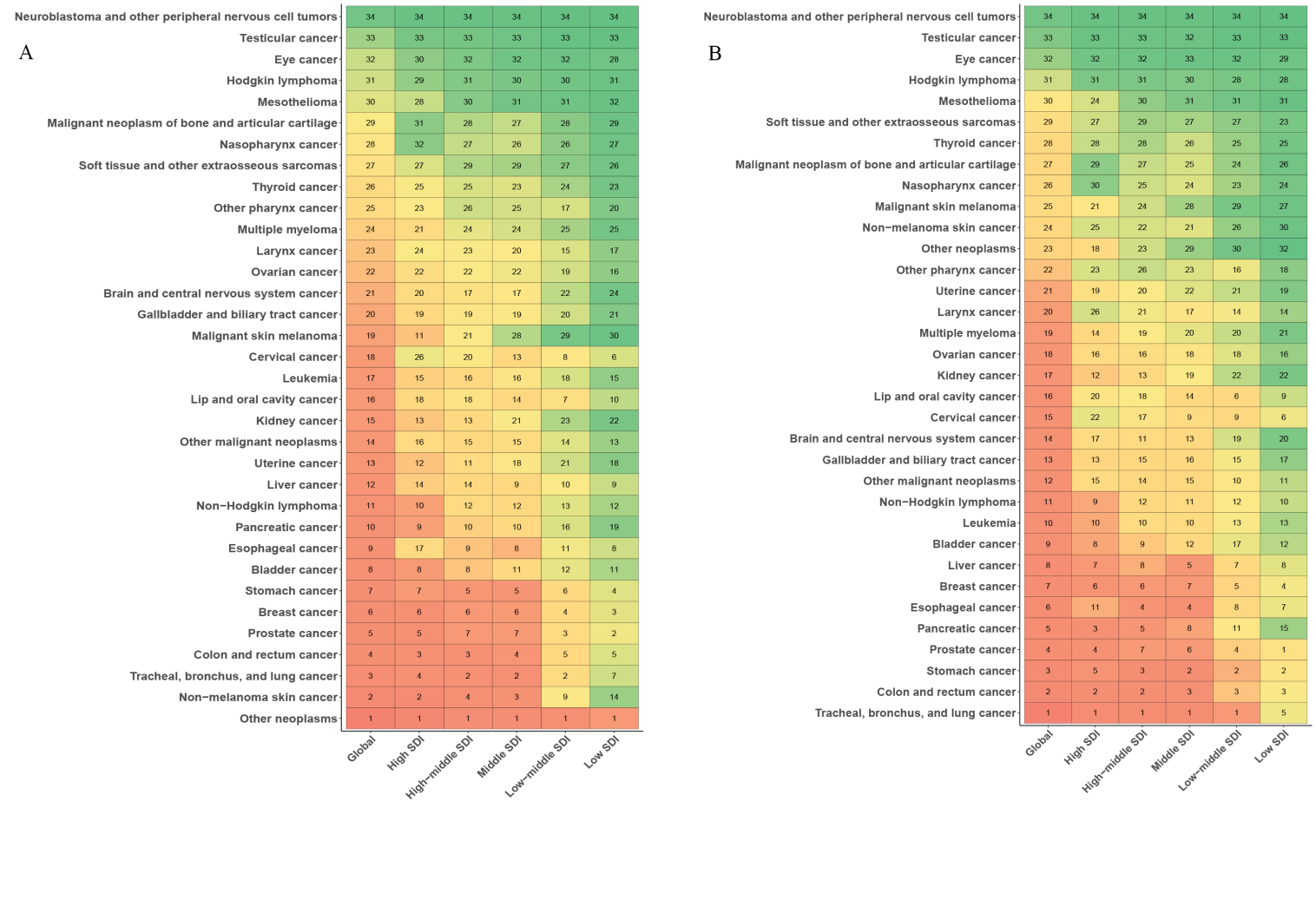
**

**FigureS2** Global ranking of absolute incidence(A) and death(B) due to gastrointestinal cancers compared to other cancers in the elder aged 60 and above in 2021 for both sexes combined.

**
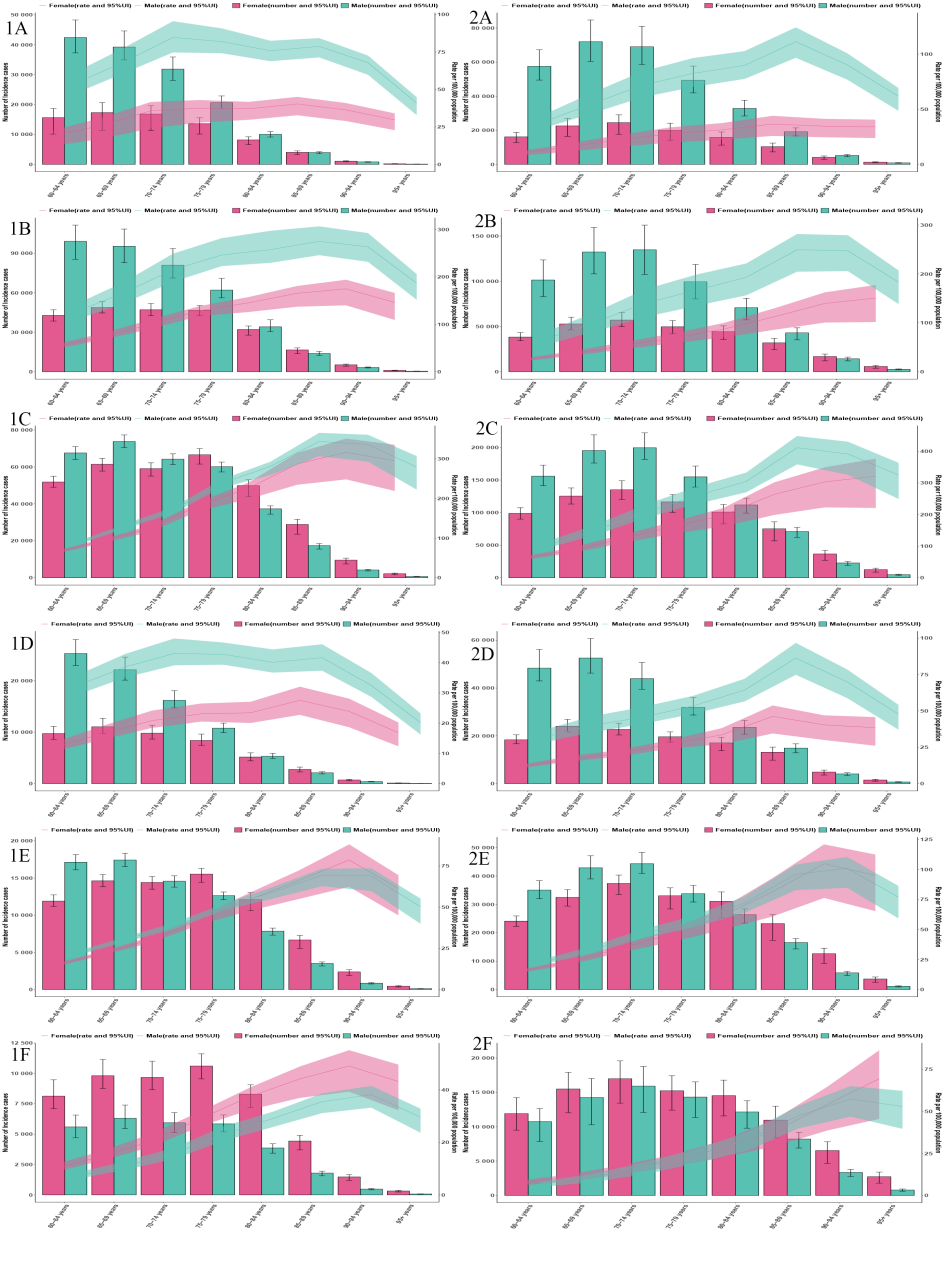

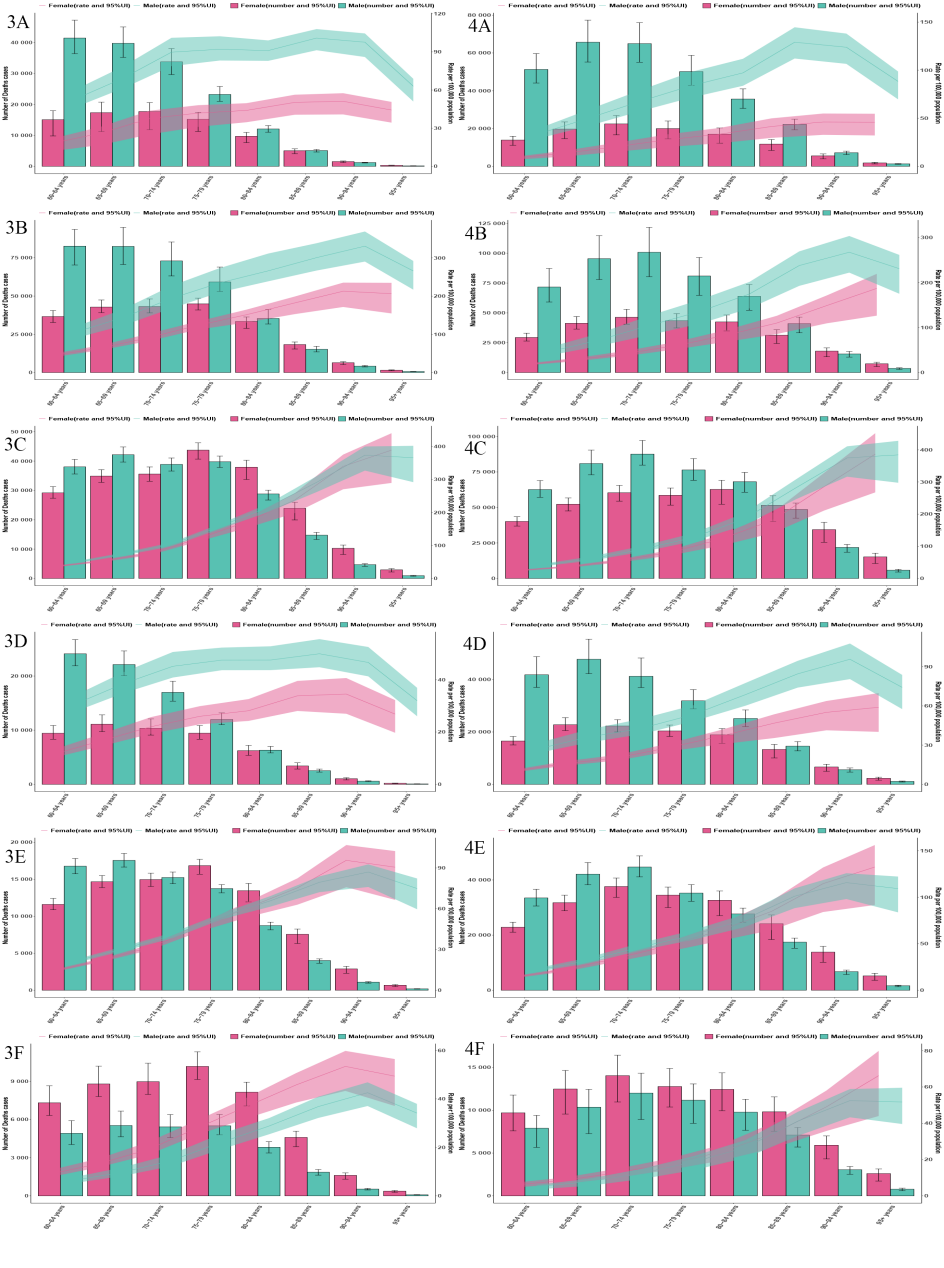
**

**FigureS3 Global counts and incidence, death rates of 6 GDSC by age and sex,1990 and 2021**

Global counts and incidence (1A,2A), death (3A,4A) rates of Geriatric Esophageal Cancer by age and sex,1990 (1A,3A) and 2021(2A,4A)

Global counts and incidence (1B,2B), death (3B,4B) rates of Geriatric Gastric Cancer by age and sex,1990 (1B,3B) and 2021(2B,4B)

Global counts and incidence (1C,2C), death (3C,4C) rates of Geriatric Colorectal Cancer by age and sex,1990 (1C,3C) and 2021(2C,4C)

Global counts and incidence (1D,2D), death (3D,4D) rates of Geriatric Liver Cancer by age and sex,1990 (1D,3D) and 2021(2D,4D)

Global counts and incidence (1E,2E), death (3E,4E) rates of Geriatric Pancreatic Cancer by age and sex,1990 (1E,3E) and 2021(2E,4E)

Global counts and incidence (1F,2F), death (3F,4F) rates of Geriatric CBCT by age and sex,1990 (1F,3F) and 2021(2F,4F)

Error bars indicate the 95% uncertainty intervals (95% UI) for incidence, death. Shading indicates the upper and lower limits of the 95% UI.

**
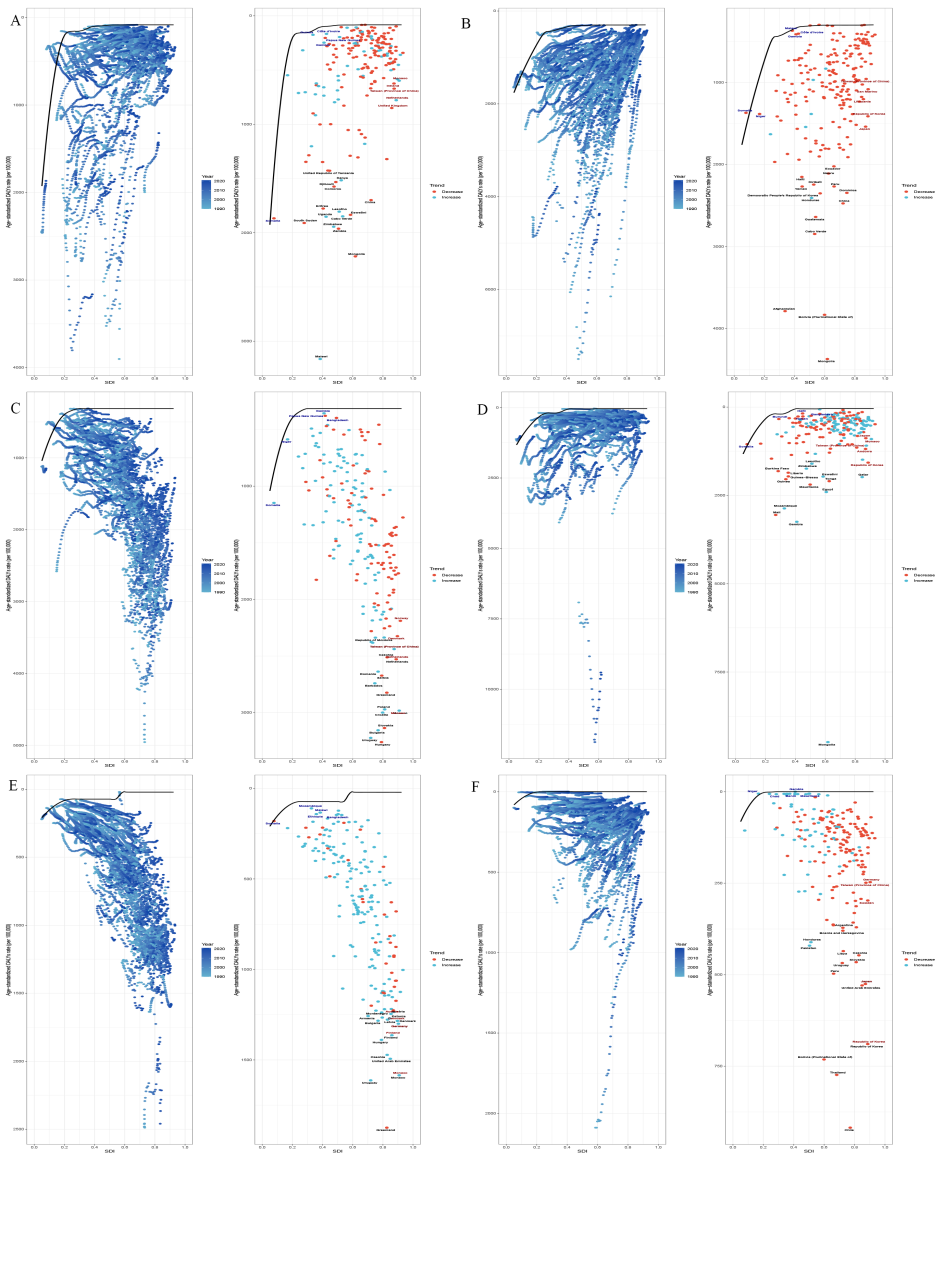
**

**FigureS4 Frontier analysis of SDI and 6 GDSC burden in 2021.**

(A) Geriatric Esophageal Cancer, (B) Geriatric Gastric Cancer, (C) Geriatric Colorectal Cancer, (D) Geriatric Liver Cancer, (E) Geriatric Pancreatic Cancer and (F) Geriatric CBCT
